# Gigobolins A–C, New Ophiobolins with Anticancer Activity from the Phytopathogenic Fungus *Drechslera gigantea*

**DOI:** 10.1021/acs.jnatprod.5c01414

**Published:** 2026-02-27

**Authors:** Marianna Carbone, Sridharan Jayamohan, Amelia Scott, Angela Boari, Ryan J. Rutledge, Nadia Cacciapuoti, Vamika Gautam, Jaquelin Aroujo, Tania Betancourt, Daniel Romo, Gangadhara Reddy Sareddy, Joseph H. Taube, Alexander Kornienko, Antonio Evidente, Maria Letizia Ciavatta

**Affiliations:** † 9327 National Research Council (CNR), Institute of Biomolecular Chemistry, Via Campi Flegrei 34, 80078 Pozzuoli, Italy; ‡ Department of Obstetrics and Gynecology, 14742 University of Texas Health San Antonio, 7703 Floyd Curl Drive, San Antonio, Texas 78229, United States; § Department of Chemistry and Biochemistry, 7174 Texas State University, 601 University Dr., San Marcos, Texas 78666, United States; ∥ National Research Council, Institute of Sciences of Food Production, Via Amendola 122/O, 70125 Bari, Italy; ⊥ Department of Biology, 14643 Baylor University, 101 Bagby Ave., Waco, Texas 76798, United States; # Department of Chemistry and Biochemistry, Baylor University, 101 Bagby Ave., Waco, Texas 76798, United States

## Abstract

Three new sesterterpenoids, gigobolins A to C (**1**–**3**), along with six known ophiobolins, were isolated and structurally characterized from the culture filtrate of the phytopathogenic fungus *Drechslera gigantea.* The structures were elucidated using extensive spectroscopic analysis, including 1D and 2D NMR, and HRESIMS, to disclose A/B ring conformation and reveal rings C and D side chain modifications of the ophiobolin tricyclic core structure. Gigobolins A (**1**) and B (**2**) together with known maydispenoid A (**9**) were evaluated for their antiproliferative activity against aggressive glioblastoma multiforme (GBM) and breast cancer cell lines, with a focus on their efficacy against chemoresistant cancer stem cell (CSC) populations. Notably, gigobolin B (**2**) and maydispenoid A (**9**) demonstrated significant activity against both GBM CSCs and multiple breast cancer cell lines. These results establish*D. gigantea* as a significant source of new anti-CSC agents and further validate the ophiobolin family as a valuable chemical scaffold for future drug development efforts targeting cancer stemness (the inherent ability to self-renew and resist conventional therapies) and therapeutic resistance.

## Graphical abstract

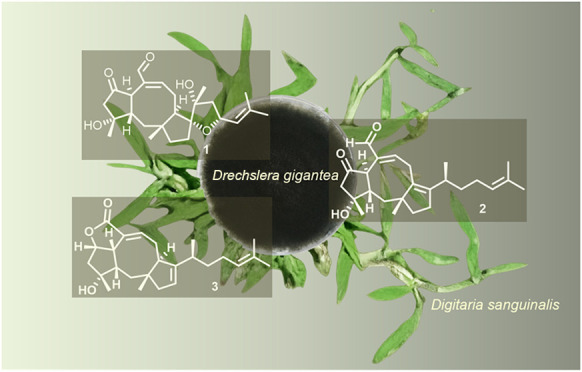

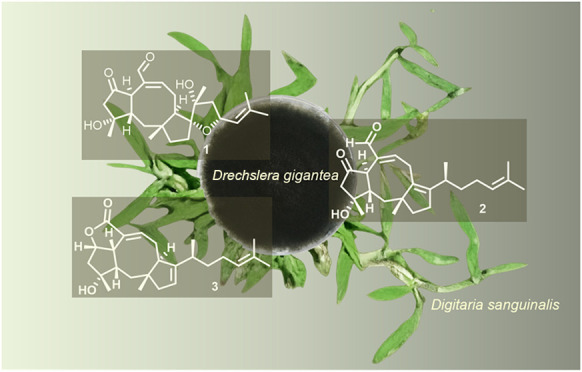

Ophiobolins are a structurally diverse family of sesterterpenoids possessing a unique 5/8/5 tricyclic core carbon skeleton. These natural products were first discovered in 1958 from *Ophiobolus miyabeanus* ^1^ and have since been isolated from a variety of fungi, including *Aspergillus*,^2^ *Emericella*,^3^ *Drechslera*,^4^ *Ulocladium*,^5^ and *Bipolaris*.^6^ The structural complexity of ophiobolins arises from the sesterterpene biosynthetic pathway, combined with various post-modifications such as oxygenations and rearrangement reactions.^7–9^ The absolute configuration of Ophiobolin A (OpA) was confirmed by X-ray crystallography in 1965.^10^

Although initially characterized as mycotoxins with potent phytotoxicity toward several crops, ophiobolins have recently attracted significant attention due to their wide-ranging biological effects. These activities include herbicidal,^4,11^ nematocidal,^12^ antibacterial,^13^ antifungal,^14^ and anti-inflammatory properties.^15^ In addition, these natural products have been investigated extensively due to their promising antiproliferative properties against various cancer cells.^16–19^ Since its discovery, more than 100 members of this family have been isolated,^20^ characterized, and tested^9,21,22^ with OpA being the most bioactive member of the family. Our research focuses on the potential of ophiobolins as promising cancer drug leads, particularly due to their capacity to overcome cancer cell resistance to apoptosis and their ability to preferentially eliminate cancer stem cells (CSCs). For instance, our group recently identified OpA as a potent cytotoxic agent against glioblastoma (GBM) cells. GBM is the most common and aggressive primary malignant brain tumor in adults, with a dismal 5-year survival rate of less than 6%, a statistic that has seen only marginal change over the past three decades.^23^ This critical need for better treatment is underscored by the limited efficacy of frontline chemotherapy, temozolomide, which is largely hampered by drug resistance.^24^ We have previously demonstrated that OpA exhibits promising activity against apoptosis-resistant GBM cells, containing defects in the p53 tumor-suppressor gene and displaying glioma stem cell (GSC) markers, such as nestin and CD44, by inducing a recently discovered mechanism of nonapoptotic cell death known as paraptosis.^25^ Furthermore, our recent work involved the identification of acid-sensitive OpA derivatives that showed good selectivity for inhibiting the proliferation of GSCs at a pH of 6.5 compared to 7.5, indicating their potential for selective targeting of the acidic tumor environment where CSCs reside.^26^

In addition, OpA’s activity against epithelial-mesenchymal transition (EMT) and cancer stemness is highly relevant to breast cancer, which is the most commonly diagnosed cancer worldwide.^27^ EMT promotes invasion, metastasis, and the generation of CSCs that are notoriously resistant to common chemotherapies, particularly for triple-negative breast cancer (TNBC), which lacks targeted therapies, unlike hormone-receptor (HR)-positive breast cancer or human epidermal growth factor receptor (HER2)-positive breast cancer.^28^ Despite the acquired resistance to chemotherapy mediated by EMT, OpA has been shown to exhibit greater potency (∼2×) against mammary epithelial cells induced to undergo EMT (via TWIST1 expression) and against breast cancer cell lines with CSC properties.^29^ Indeed, our group recently showed that a subcytotoxic dose of OpA led to the reversal of EMT, evidenced by the gain of E-cadherin expression, loss of vimentin, and reduced migration and CSC properties.^29^

Recently, we reported the results of our semisynthetic studies, revealing that OpA analogs possessing unsaturated 1,4-ketoaldehyde moiety, were selective in eliminating glioma stem cells.^30^ We have also reported on the structural features of OpA that mediate the anti-EMT/anti-CSC effects in breast cancer.^30^ In an ongoing effort to discover new bioactive ophiobolin congeners with anticancer stem cell activity, we reanalyzed the ophiobolin content of a *Drechslera gigantea* culture filtrate extract. This fungal species, closely related to the *Bipolaris* genus, is a known ophiobolin producer.^4^ Chemical analysis of a *D. gigantea* strain isolated in Florida resulted in the isolation and structural characterization of three new ophiobolins, which we have named gigobolins A–C (**1**–**3**), alongside six known ophiobolin derivatives (**4**–**9**) (Figure 1). Herein, we report the isolation, structure elucidation, and evaluation of gigobolins A (**1**) and B (**2**) (together with the known maydispenoid A, **9**) for their bioactivity against GBM and multiple subtypes of breast cancer cell lines, including specific models for cancer stem cells and EMT, using OpA as the control.

**Figure 1:**
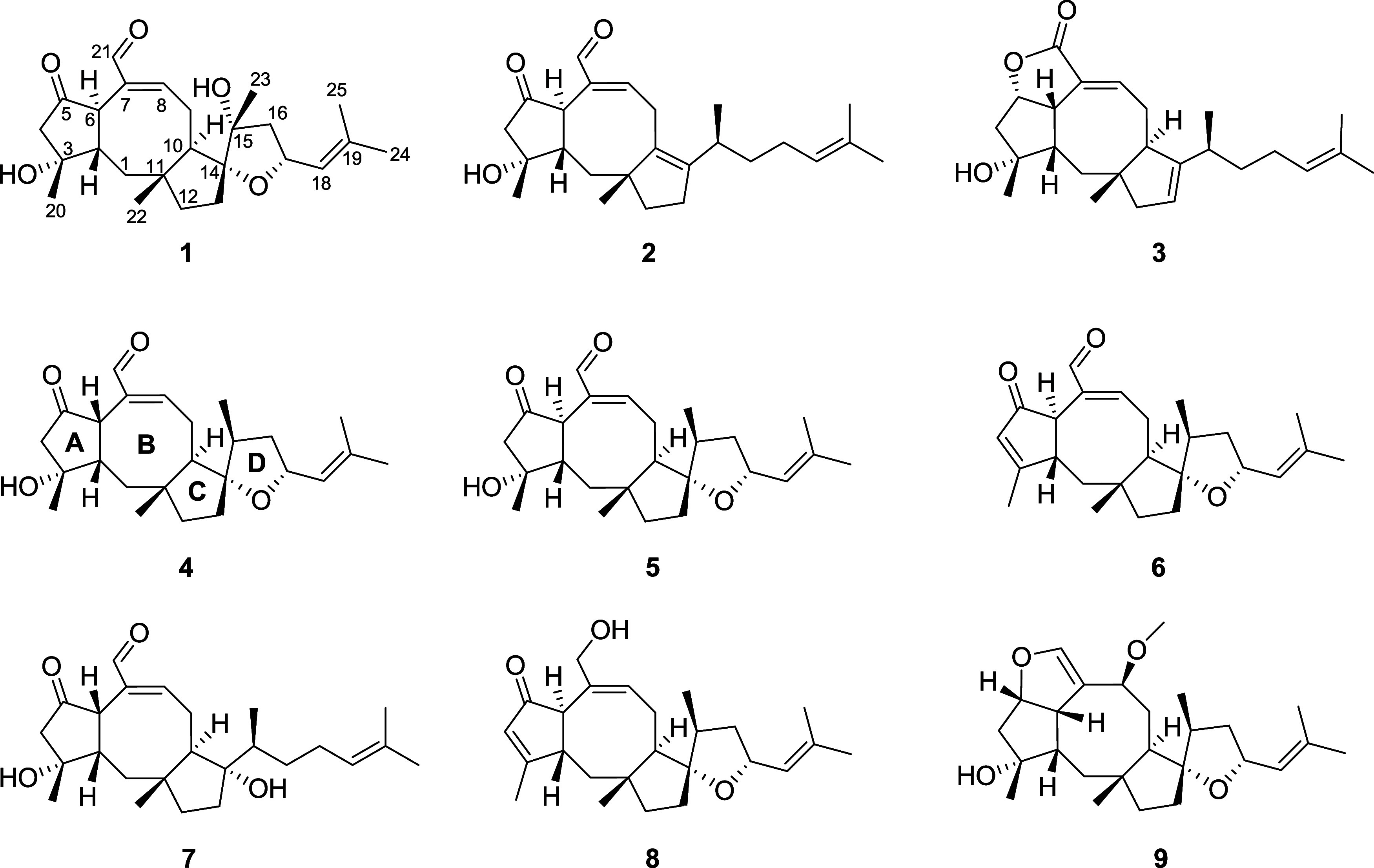
Structures of new gigobolins A–C (**1**–**3**) and known metabolites (**4**–**9**) isolated in this work.

## Results and Discussion

The EtOAc crude extracts from *D. gigantea* culture filtrate were submitted to sequential fractionation steps involving both crystallization and chromatographic methods yielding the new gigobolins A–C (**1**–**3**) along with six known ophiobolins (**4**–**9**) as fully described in the Experimental Section. The six known compounds were identified as OpA (**4**)^22^, 6-*epi*-OpA (**5**),^22^ 3-anhydro-6-*epi*-OpA (**6**),^22^ ophiobolin B (OpB, **7**),^22^ ophiobolin I (OpI, **8**),^22^ and maydispenoid A^31^ (**9**) by comparison of their MS and NMR data with those of the literature. The planar structures of the new gigobolins **1**–**3** were established by analyzing the 2D NMR spectra in combination with HRESIMS, whereas the relative stereochemistry was assigned by the examination of proton and carbon resonance values and correlations in the NOESY spectrum.

Gigobolin A (**1**), as described below, is structurally similar to 6-*epi*-OpA (**5**), specifically sharing the identical ring ABC framework, with the only structural difference being the tertiary alcohol at C-15 in **1**. Compound **1** gave a [M + Na]^+^ ion peak at *m*/*z* 439.2466 in HRESIMS corresponding to the molecular formula C_25_H_36_O_5_Na requiring eight indices of hydrogen deficiency. Both ^1^H and ^13^C NMR spectra of **1** strongly resembled those of the co-occurring 6-*epi*-OpA (**5**). In particular, similar to 6-*epi*-OpA (**5**), the proton spectrum of gigobolin A (**1**) displayed the characteristic signals of an unsaturated aldehydic group (δ_H_ 9.19, s, 1H, H-21), an olefinic proton (δ_H_ 6.84, dd, *J* = 6.6, 2.5 Hz, 1H, H-8), four sp^3^ methines [δ_H_ 4.50 (td, *J* = 8.8, 4.1 Hz, 1H, H-17), δ_H_ 3.34 (d, *J* = 10.6 Hz, 1H, H-6), δ_H_ 2.64 (app dd, *J* = 15.0, 3.5 Hz, 1H, H-10); δ_H_ 2.14 (m, 1H, H-2)], and an isolated methylene group at δ_H_ 3.07 (d, *J* = 16.6 Hz, 1H, H-4α) and at δ_H_ 2.43 (dd, *J* = 16.6, 1.5 Hz, 1H, H-4β). The presence of an additional alcohol group in **5**, as suggested by its molecular formula, was corroborated by the distinct, downfield methyl singlet (δ_H_ 1.37, s, H_3_-23) and the absence of the methyl doublet (δ_H_ 1.02, d, *J* = 6.7 Hz, 3H, H_3_-23) observed on the spiro tetrahydrofuran ring of 6-*epi*-OpA (**5**). Furthermore, the ^13^C NMR spectrum of **1** showed an oxygenated quaternary carbon signal resonating at δ_C_ 80.6 (C-15, C) replacing the methine carbon at δ_C_ 35.8 (C-15, CH) observed in **5**. As expected, this quaternary carbon showed a correlation with H_3_-23 in the HMBC spectrum (Figure 2a).

**Figure 2:**
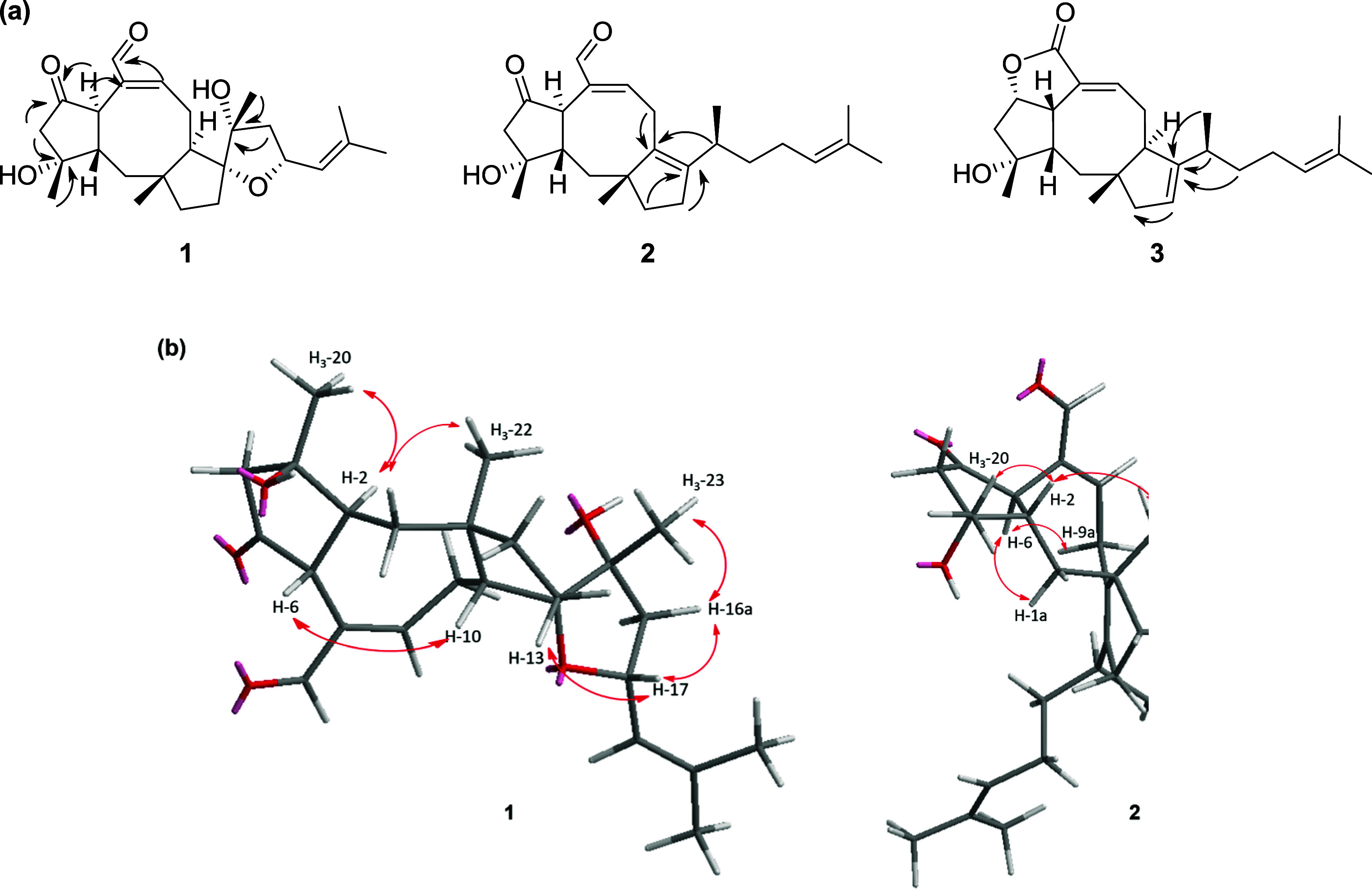
(a) Main HMBC (black arrows) correlations of gigobolins A–C (**1**–**3**). (b) Selected NOE (red arrows) for gigobolins A (**1**) and B (**2**).

The relative configuration of gigobolin A (**1**) was elucidated by analyzing both NOESY correlations and carbon and proton resonances. The *trans* ring junctions of the 5/8/5 carbon framework in **1** was inferred by correlations in the NOESY spectrum between H-6 (δ_H_ 3.34) and H-10 (δ_H_ 2.64), and between Me-22 (δ_H_ 0.89) and H-2 (δ_H_ 2.14). The latter resonance also showed a cross peak with Me-20 thus indicating the α-orientation of the hydroxy group at C-3 (Figure 2b). The proposed C-2/C-6 *trans*-junction was also supported by the resonance value of C-1 (δ_C_ 41.2) that is downfield shifted with respect to the ophiobolins with a H-2/H-6 *cis*-junction (δ_C_ 35.0), and by the proton values of H-2 and H-8 as well as of H-4β (see Table 1). It has been noted that ophiobolins exhibiting a *trans* C-2/C-6 junction with H-6 in the α-configuration (such as **1**) typically show shielded H-2 and H-8 proton values by about 0.2–0.3 parts per million (ppm). This trend diverges from that of the H-6 β-series, where the C-4 β-proton is instead deshielded. The downfield shift of H-9a at δ_H_ 3.75 (usually resonating at ∼δ_H_ 2.80) strongly suggests the α-orientation of the hydroxy group at C-15, whereas the NOESY cross peak correlations of H-16a (δ_H_ 2.31) with both H_3_-23 and H-17, which in turn had a correlation with H_2_-13 (δ_H_ 1.57–1.48), imply the α-orientation of the side chain at C-17 (Figure 2b). Additionally, the absolute configuration of gigobolin A (**1**) is proposed to be the same as that of 6-*epi*-OpA (**5**) by comparison of their ECD curves (Figure 3). Furthermore, the analysis of HSQC and HMBC spectra aided the assignments of all proton and carbon resonances, as reported in Table 1.

**Figure 3:**
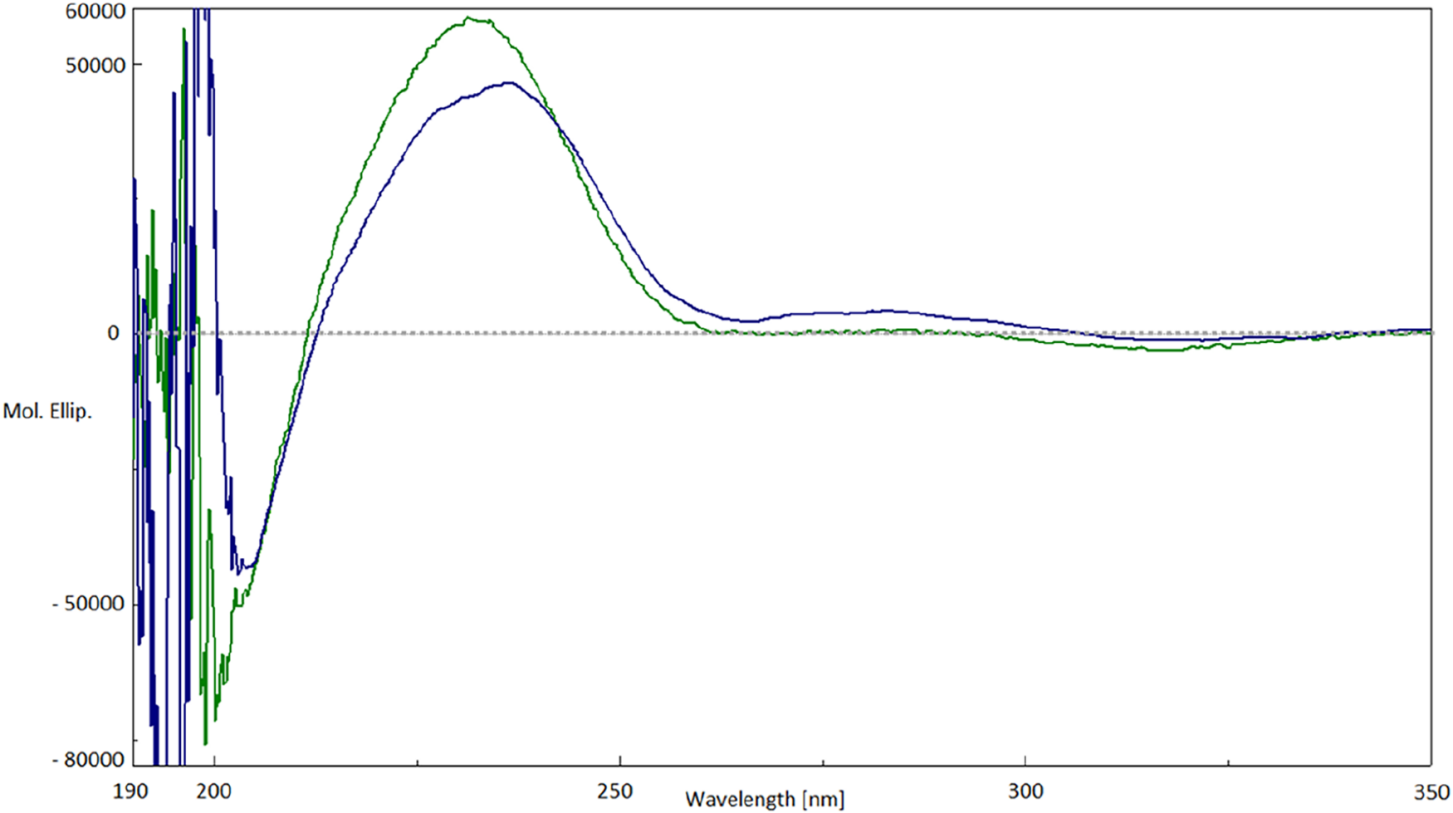
ECD plots of gigobolin A (**1**) (blue line) and 6-*epi*-OpA (**5**) (green line).

**Table 1:** ^1^H and ^13^C NMR Data^a^ ^,^ ^b^ of Gigobolins A and B (1 and 2)

	**1**		**2**	
no#	δ_C_	δ_H_, mult. (J (Hz))	δ_C_	δ_H_, mult. (J (Hz))
1	41.2, CH_2_	1.78–1.68, m	38.8, CH_2_	1.78–1.73, m
2	49.7, CH	2.14, m	48.7, CH	2.15–2.05, m
3	76.7, C		77.1, C	
4	54.9, CH_2_	3.07, d (16.6)	54.7, CH_2_	2.89, d (17.3)
		2.43, dd (16.6, 1.5)		2.48, dd (17.3, 1.5)
5	216.7, C		215.1, C	
6	48.6, CH	3.34, d (10.6)	51.1, CH	3.36–3.31, m
7	141.6, C		144.1, C	
8	158.9, CH	6.84, dd (6.5, 1.9)	153.3, CH	6.80, dd (9.4, 7.1)
9	30.5, CH_2_	3.75, dt (20.0, 2.9, 1.9)	27.5, CH_2_	3.36–3.31, m
		2.33, app dd (15.0, 6.5)		3.11–3.07, m
10	51.7, CH	2.64, dd (15.0, 2.9)	139.1, C	
11	43.3, C		48.9, C	
12	41.5, CH_2_	1.93, ddd (12.3, 11.8, 5.1)	26.5, CH_2_	1.89–1.80, m
		1.45–1.39, m		
13	28.6, CH_2_	1.57–1.48, m	27.5, CH_2_	2.23–2.16, m
				2.14–2.06, m
14	97.8, C		143.9, C	
15	80.6, C		32.8, CH	2.55, dq (7.0)
16	50.5, CH_2_	2.31, dd (13.6, 8.8)	35.7, CH_2_	1.89–1.80, m
		1.81, dd (13.6, 4.1)		1.37, ddd (15.2, 8.4, 6.3)
17	69.2, CH	4.50, ddd (8.8, 8.8, 4.1)	43.6, CH_2_	1.69–1.66, m
				1.63–1.52, m
18	127.2, CH	5.27, d (8.8)	124.6, CH	5.08 ddd (8.6, 7.1, 1.5)
19	135.8, C		131.7, C	
20	26.1, CH_3_	1.43, s	25.6, CH_3_	1.45, s
21	194.4, CH	9.19, s	192.4, CH	9.36, s
22	22.7, CH_3_	0.89, s	23.5, CH_3_	0.79, s
23	21.4, CH_3_	1.37, s	19.9, CH_3_	0.97, d (7.0)
24	18.4, CH_3_	1.66, s	17.9, CH_3_	1.59, overlapped s
25	25.9, CH_3_	1.73, s	25.6, CH_3_	1.68, d (1.5)

a The spectra were recorded in CDCl_3_ at 600 MHz.

b Assignments aided by COSY, *ed*-HSQC, and HMBC (*J* = 7) experiments.

Gigobolin B (**2**) is structurally similar to ophiobolin B (OpB, **7**), with the differences being the *trans* vs *cis* AB-ring junction (C-6) and an apparent dehydration of **7** at C-10/C-14 to give gigobolin B (**2**). The molecular formula C_25_H_36_O_3_Na^+^ of **2**, derived from the sodiated ion peak at *m*/*z* 407.2544 [M + Na]^+^, implies dehydration and an additional hydrogen deficiency index compared to the molecular formula of **7** (C_25_H_38_O_4_). HMBC correlations (Figure 2a), between H-9 and H-15 with C-10 (δ_C_ 139.1), as well as H-13 with C-14 (δ_C_ 143.9) indicate the presence of a tetrasubstituted olefin in the C-ring of the ophiobolins. Furthermore, only three methine correlations are observed in the HSQC spectrum, supporting that the methine at C-10, which is typically present in the ophiobolins, is absent in this new congener.

The C-2/C-6 *trans*-junction of **2** was evident by NOESY correlations between H-2 (δ_H_ 2.15–2.05, m) and both Me-20 (δ_H_ 1.45, s, 3H) and Me-22 (δ_H_ 0.79, s, 3H), and between H-6 (δ_H_ 3.36–3.31, m, 1H) and both H-1 (δ_H_ 1.78–1.73, m, 1H) and H-9b (δ_H_ 3.11–3.07, m, 1H) (Figure 2b). In addition, some significant differences were also observed in the chemical shift of protons H-2 and H-8 which were both shifted upfield with respect to **7** that suggested a 6-*epi* configuration. The configuration at C-15 was not fully established, but by analogy, it was proposed to be the same as that in the co-occurring OpB (**7**). All proton and carbon resonances are reported in Table 1.

The minor ophiobolin in *D. gigantea* extract named gigobolin C (**3**) has a sodiated ion peak of *m*/*z* 407.2552 corresponding to the same molecular formula of C_25_H_36_O_3_Na^+^ as ophiobolin X^2^ (OpX) and 6-*epi*-ophiobolin M^32^ (Figure 4). The resonances of both the carbonyl lactone at C-21 (δ_C_ 172.0) and the methine at C-5 (δ_C_ 81.6; δ_H_ 4.98, dd, *J* = 7.1, 5.7, 1H) were almost superimposable with the same signals in OpX (**10**) (Figure 4 and Table 2). On the other hand, the olefin C-13 (δ_C_ 120.8) and C-14 (δ_C_ 150.3) in **3** also aligned with the reported resonances of carbons C-13 and C-14 for 6-*epi*-OpM (**11**) (Figure 4) as shown in Table 2. HMBC correlations as depicted in Figure 2a also corroborated the configuration of C-13 and C-14. Unfortunately, full stereochemical determination was hampered by degradation that occurred in the NMR tube during experiment acquisitions. However, based on the comparison of NMR resonances of **3** and those of published ophiobiolin family members containing C-21-lactone groups,^2,33^ we propose the relative stereochemistry at C-5, C-6, and C-2 as all *cis*.

**Figure 4:**
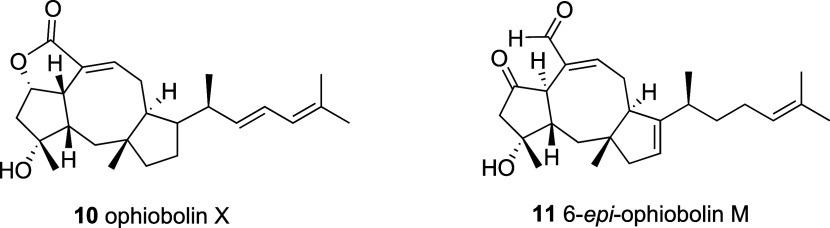
Structure of known ophiobolin X (**10**) and 6-*epi*-ophiobolin M (**11**).

**Table 2:** ^1^H and ^13^C NMR Data of Gigobolin C (**3**),^a^ ^,^ ^b^ Ophiobolin X,^c^ and 6-*epi*-OpM^c^

	**3**		**10**		**11**	
no	δ_C_, mult	δ_H_, mult. (J (Hz)	δ_C_, mult	δ_H_, mult. (J (Hz)	δ_C_, mult	δ_H_, mult. (J (Hz)
1	35.5, CH_2_	2.33–2.29, m	35.1, CH_2_	1.39, m	41.8, CH_2_	1.84, m
		1.32–1.24, m		1.63, dd (14.4, 3.7)		
2	52.7, CH	2.07–2.00, m	51.8, CH	1.96, ddd (13.0, 11.0, 3.7)	49.8, CH	2.10, m
3	80.5, C		80.4, C		76.9, C	
4	48.6, CH_2_	2.27, d (15.1)	46.7, CH_2_	1.85, m	55.1, CH_2_	3.15, d (16.5)
		1.89, dd (15.0, 5.7)		2.26, bd (15.0)		2.44, dd (16.5, 1.5)
5	81.6, CH	4.98, dd (7.1, 5.7)	81.5, CH	4.95, brdd (6.4, 6.4)	217.5, C	
6	44.9, CH	3.68–3.62, m	44.5, CH	3.57, ddd (10.0, 6.4, 2.0)	49.0, CH	3.25, d (10.5)
7	131.1, C		130.3, C		142.2, C	
8	140.6, CH	7.03, dd (8.5, 8.4, 2.4)	140.7, CH	6.91, ddd (8.5, 8.5, 2.0)	158.8, CH	6.90, dd (7.5, 2.5)
9	23.5, CH_2_	2.48, dd (13.1, 8.9)	25.3, CH_2_	2.78, brdd (13.0, 8.5)	32.5, CH_2_	2.91, ddd (20.0, 4.0, 2.5)
		2.08–1.99, m		1.88, m		2.19, m
10	58.3, CH	2.42, app dt (11.4, 3.3)	54.4, CH	1.73, m	51.3, CH	3.18, m
11	46.8, C		43.5, C		44.3, C	
12	46.9, CH_2_	1.97–1.92, m	43.3, CH_2_	1.41, m	51.7, CH_2_	2.36, m
		1.89, dd (15.0, 5.7)		1.43, m		2.15, m
13	120.8, CH	5.30–5.29, m	26.7, CH_2_	1.55, m	120.3, CH	5.28, m
				1.79, m		
14	150.3, C		47.4, CH	2.13, m	149.3, C	
15	32.1, CH	2.15–2.09, m	35.5, CH	2.70, m	31.6, CH	2.08, m
16	34.2, CH_2_	1.78–1.75, m	137.5, CH	5.23, dd (9.1, 9.1)	35.2, CH_2_	1.12, m
		1.32–1.24, m				1.46, m
17	25.4, CH_2_	1.98–1.93, m	122.0, CH	6.01, m	25.1, CH_2_	1.94, m
		1.32–1.24, m				
18	124.5, CH	5.10, app t (7.1, 1.5)	120.2, CH	6.00, m	124.3, CH	5.10, m
19	132.1, C		135.4, C		131.7, C	
20	25.9, CH_3_	1.28, s	25.9, CH_3_	1.26, s	25.9, CH_3_	1.45, s
21	172.0, C		171.7, C		194.0, C	9.23, s
22	19.2, CH_3_	1.60, s	18.3, CH_3_	0.99, s	22.8, CH_3_	0.91, s
23	18.4, CH_3_	1.04, d (6.7)	20.3, CH_3_	0.91, d (6.7)	18.1, CH_3_	1.09, d (6.5)
24	17.8, CH_3_	1.60, s	18.1, CH_3_	1.74, s	17.7, CH_3_	1.60, s
25	26.0, CH_3_	1.69, d (1.5)	26.5, CH_3_	1.81, s	25.7, CH_3_	1.69, d (1.0)

a The spectra were recorded in CDCl_3_ at 600 MHz.

b Assignments aided by COSY, *ed*-HSQC, and HMBC (*J* = 7) experiments.

c NMR values (CDCl_3_) reported from the literature.

We evaluated gigobolins A (**1**) and B (**2**) and maydispenoid A (**9**), which have not been previously tested for anticancer activities, in our models of GBM and breast cancer with a focus on studying the effects of these compounds on diverse breast cancer subtypes, including those with and without epithelial-mesenchymal transition (EMT) features, HMLE versus HMLE-TWIST (Table 3).

**Table 3:** In Vitro Antiproliferative Activities^a^ of OpA (**4**) Gigobolins A (**1**) and B (**2**) as well as Maydispenoid A (**9**) against Glioblastoma and Breast Cancer Cell Lines, Including Cancer Stem cell-enriched cultures.

cell line	cell type	OpA (**4**)	gigobolin A (**1**)	gigobolin B (**2**)	maydispenoid A (**9**)
T98G	GBM	5.97 ± 1.47	780 ± 21^b^	NT^c^	650 ± 130
U87	GBM	1.28 ± 0.72	39.6 ± 10.5	NT	67.1 ± 20.5
GSC 040815	patient-derived GSCs	0.088 ± 0.005	53.30 ± 7.58^d^	8.10 ± 0.231	8.62 ± 1.863
GSC 082209	patient-derived GSCs	0.087 ± 0.006	20.81 ± 4.70	6.50 ± 0.43	6.34 ± 0.05
HMLE	noncancer EMT^–^	0.075 ± 0.008	3.24 ± 0.66	0.95 ± 0.15	1.60 ± 0.19
HMLE-TWIST	noncancer EMT^+^	0.035 ± 0.008	1.99 ± 0.46	0.77 ± 0.10	1.52 ± 0.21
MCF7	HR+ breast cancer, EMT^–^	0.243 ± 0.029	44.60 ± 3.81	29.97 ± 0.94	24.03 ± 0.03
SK-BR3	HER2+ breast cancer, EMT^–^	0.257 ± 0.047	46.00 ± 2.88	49.60 ± 17.81	25.23 ± 0.15
MDA-MB-468	TNBC, EMT^–^	0.188 ± 0.006	34.53 ± 0.58	32.17 ± 0.90	23.50 ± 0.85
MDA-MB-231	TNBC, EMT^+^	0.328 ± 0.038	83.70 ± 0.45	72.63 ± 7.61	78.33 ± 2.33
BT-20	TNBC, EMT^+^	0.631 ± 0.028	48.00 ± 3.15	41.70 ± 2.90	37.03 ± 1.69

a GI_50_ is the compound concentration that reduces by 50% of the growth of indicated cells (as compared to the control value) after having cultured the cells for 48 h with the compound in the table, as determined with the MTT assay. Each experiment was carried out in six replicates as described in the Experimental Section.

b Low confidence in the results—maximal cytotoxicity not reached with highest concentration of drug tested.

c NT = not tested due to insufficient amount of material.

d Low confidence in the results—high variation among replicates. GSCs = glioblastoma stem cells; HR+ = hormone-receptor positive, HER2+ = human epidermal growth factor receptor 2 positive, TNBC = triple-negative breast cancer.

We included in our cell line panels GBM cell lines T98G and U87 in addition to patient-derived glioblastoma stem cell lines, GSC 040815 and GSC 082209, grown as neurospheres that were previously analyzed for stemness markers and characterized for consistency by histology with the primary tumor.^34,35^ In addition, given OpA’s enhanced activity against mammary epithelial cells induced to undergo EMT and thus against breast cancer cell lines with CSC properties, we selected EMT^+^ and EMT^–^ epithelial (HMLE) cell lines as models for breast cancer stem cells, in addition to multiple breast cancer subtypes,^29,30^ as indicated in Table 3. As expected based on our previous studies,^30^ OpA (**4**) displayed pronounced selectivity toward growth inhibition of GSCs over transformed GBM cells registering nanomolar GI_50_ values against patient-derived GSC neurospheres (Table 3). Similarly, although with a lower level of selectivity, OpA (**4**) effectively inhibited the growth of mammary epithelial cells induced to undergo epithelial-mesenchymal transition (EMT) via Twist expression (HMLE-TWIST vs HMLE), despite their acquired resistance to multiple chemotherapeutic agents. It was also highly active against multiple subtypes of breast cancer cells.^29,30^

In general, it is well recognized that variations in the tetrahydrofuran part of the ophiobolin skeleton, i.e., ring D, do not have a pronounced effect on cytotoxic potency in this family of natural products. For example, OpA (**4**), with an intact tetrahydrofuran ring, and OpB (**7**), with an open tetrahydrofuran ring D, are both equally low nanomolar against CLL leukemia cells.^36^ Therefore, the reduced potency of gigobolins A (**1**) and B (**2**) is unlikely due to the hydroxylation at C-15 (in **1**) and/or elimination of ring D (in **2**). The important factor here is the *trans* AB-ring fusion in **1** and **2** compared to *cis* in **4** and **7**, i.e., the configuration at C6. Indeed, the change of this ring fusion from *cis* to *trans*, as is found in 6-*epi*-OpA (**5**), leads to a drop in cytotoxic potency by ∼1 to 2 orders of magnitude.^37^ It is unfortunate that we have run out of material and could not complete the evaluation of gigobolin B (**2**) against GBM cells, but its low micromolar potency against patient-derived GSC cells is an interesting finding for future research toward therapies directed toward addressing cancer stems cells.

Maydispenoid A (**9**) is the only ophiobolin family member in which the C-21-aldehyde is closed into a dihydrofuran ring; thus, this structure represents a new chemotype within the ophiobolin family. Notably, this congener exhibited cytotoxicity toward GSCs with a GI_50_ an order of magnitude lower than that toward transformed GBM. This compound was also active against HMLE cells, although no selectivity toward HMLE-EMT^+^ cells was observed here.

## Conclusion

In summary, our ongoing investigation into the diverse chemical profile of the phytopathogenic fungus *D. gigantea* led to the successful isolation and structural characterization of three new sesterterpenoids, which we named gigobolins A–C (**1**–**3**). Their distinctive structural variations in the C and D rings and the oxygenation patterns compared to known congeners further expand the structural diversity of the ophiobolin family.

The newly identified compounds were evaluated for their therapeutic potential against two highly aggressive and drug-resistant cancer types: glioblastoma (GBM) and triple-negative breast cancer. Gigobolins A (**1**) and B (**2**), along with the known analog maydispenoid A (**9**), demonstrated significant antiproliferative activity, with gigobolin B and maydispenoid A exhibiting promising properties against GBM stem cells (GSCs), the subpopulation responsible for tumor recurrence and chemoresistance. For maydispenoid A, this is the first demonstration of promising anticancer activity of a congener possessing a C-21-dihydrofuran in lieu of the ketoaldehyde moieties.

These findings validate *D. gigantea* species as a useful source of bioactive ophiobolins and reinforce the potential of this sesterterpenoid family as anticancer agents, especially for recalcitrant tumors driven by stemness and drug resistance. Future work will continue to focus on isolation strategies for bioactive ophiobolins, comprehensive structure–activity relationship (SAR) studies, and in-depth mechanistic investigations to elucidate the specific molecular targets and modes of action, paving the way for the clinical development of these new anti-CSC agents.

## Experimental Section

### General Experimental Procedures

UV spectra were measured in MeOH on a Jasco V-530 spectrophotometer in MeOH. CD curves were recorded in MeOH on a JASCO F815 spectropolarimeter (Jasco, Lecco, Italy). 1D and 2D NMR spectra were acquired in CDCl_3_ on a Bruker Avance III HD 400 MHz spectrometer equipped with a CryoProbe Prodigy, and on on a DRX 600 spectrometer equipped with a three-channel inverse (TCI) CryoProbe. Chemical shift values were reported in parts per million (ppm) referring to CHCl_3_ (δ_H_ 7.26 for proton and δ_C_ 77.0 for carbon). High-resolution mass spectra (HRESIMS) were acquired on a Q-Exactive hybrid quadrupole-orbitrap mass spectrometer (Thermo Scientific, San Jose, CA). NMR experiments were recorded at the ICB-NMR Service Centre. Analytical TLC was performed on silica gel (Kieselgel 60, F_254_, 0.25), and the spots were visualized by exposure to UV radiation (253 nm), or iodine vapor, or by spraying first with 10% H_2_SO_4_ in MeOH and then with 12% sulfuric acid and CeSO_4_ in water, followed by heating.

### Fungal Strain Growth

The strain of *D. gigantea* was isolated in Florida from naturally infected large crabgrass (*Digitaria sanguinalis*). It was stored and growth in flasks (1 L) containing a mineral-defined medium detailed as previously reported.^4^

### Extraction and Purification of Gigobolins

The lyophilized fungal culture filtrates (10.9 L) was suspended in distilled water (1.5 L, final pH 4.5) and extracted with ethyl acetate (3 × 500 mL). The organic extracts were combined and evaporated under reduced pressure, giving a brown oily residue (3.0 g). Mycelium was also extracted with ethyl acetate (2 × 500 mL) using ultrasound to facilitate cell disruption. The organic residues were filtered, and the solvent was evaporated to obtain 3.0 g of blackish gum. After TLC comparison, the two extracts were combined and loaded onto a silica gel column, using chloroform/isopropanol (95:5, 8:2) first, and then flushed with methanol. A total of eight fractions (F1–F8) were collected and analyzed by TLC and ^1^H NMR.

Fraction F2 (2.96 g), which appeared to contain mainly OpA (**4**), was repeatedly treated with *n*-hexane/EtOAc at −20 °C giving 1.3 g of a white precipitate. Proton spectrum of this precipitate confirmed the presence of pure OpA. Fraction F3 (1.3 g) was purified via flash chromatography using a gradient of diethyl ether in petroleum ether (7:3, 6:4, 1;1, 4:6, 3:7, 1:9) and finally with MeOH yielding 30 subfractions (F3-1–F3-30). Fractions F3-12/F3-13/F3-14 (430.0 mg) were analyzed by ^1^H NMR showing to contain 6-epi-OpA (**5**). Fractions F3-15/F3-16/F3-17/F3-18 (160.0 mg) contained ophiobolin I (**8**). Fractions F3-20/F3-21/F3-22 (20 mg) analyzed by TLC were shown to contain ophiobolin I and a more polar UV absorbing compound. Purification of this fraction by SPE-RP18-cartridge in MeOH/H_2_O 1:1 gave 17 subfractions that after NMR analysis were identified as the new gigobolin A (**1**, 3.4 mg) and additional amount of ophiobolin I (2.0 mg). Fraction F3-7/F3-8/F3-9 (19.0 mg) contained less polar ophiobolins, which were purified by HPLC on an RP-amide semipreparative column using a gradient of CH_3_CN/H_2_O as the eluent (from 40:60 to 100% in 30 min, flow 2.0 mL/min). Two main peaks were collected and subjected to NMR and were identified as 3-anhydro-6-*epi*-OpA (**6**) and maydispenoid A (**9**). Fraction F3-10 (11.3 mg) was also subjected to HPLC purification using the same semipreparative column and eluting with CH_3_CN/H_2_O (from 70:30 to 100% in 30 min, flow 2.0 mL/min). Two main peaks were collected that after NMR analysis have been identified as gigobolin B (**2**, 1.0 mg) and gigobolin C (**3**, 0.5 mg). The more polar fraction F3-27 (33.0 mg) was subjected to further purification on a silica gel column using a mixture of petroleum ether and diethyl ether to give 2.0 mg of OpB (**7**).

#### Gigobolin A (**1**)

White powder; [α]_D_ = +17.4 (c 0.03, CHCl_3_); UV (MeOH) λ_max_ (log ε) 276 (1.03), 225 (1.84), 203 (1.87) nm; ECD (MeOH) θ_235_ + 45,736, θ_281_ + 3982, θ_318_ – 1265; ^1^H and ^13^C NMR; see Table 1; HR-ESI-MS *m*/*z* 439.2466 (calcd. for C_25_H_36_O_5_Na, 439.2455).

#### Gigobolin B (**2**)

Pale yellow oil; [α]_D_ = +54.8 (c 0.09, CHCl_3_); UV (MeOH) λ_max_ (log ε) 277 (1.01), 224 (1.60), 202 (1.72) nm; ECD (MeOH) θ_228_ + 1680, θ_259_ – 306, θ_296_ + 635; ^1^H and ^13^C NMR; see Table 1; HR-ESI-MS *m*/*z* 407.2544 (calcd. for C_25_H_36_O_3_Na, 407.2557).

#### Gigobolin C (**3**)

White amourphous powder; ^1^H and ^13^C NMR; see Table 2; HR-ESI-MS *m*/*z* 407.2552 (calcd. for C_25_H_36_O_3_Na, 407.2557).

#### Maydispenoid A^31^ (**9**)

[α]_D_ = +54.8 (c 0.04, CH_3_OH), lit.^31^ [α]_D_ = +43.3 (c 0.3, MeOH); ECD (MeOH) θ_211_ + 6631; NMR data for compound **9** in CDCl_3_. ^1^H NMR δ: 6.13 (s, H-21), 5.16 (br d, 8.4 Hz, H-18), 4.41 (ddd, 8.4, 7.2, 1.5, H, H-17), 4.20 (app d, 7.0 Hz, H-5), 3.47 (dd, 10.0, 6.2 Hz, H-8), 3.24 (s, OCH_3_-8), 2.51 (dd, 15.2, 7.0 Hz, H-4a), 2.43 (app d, 3.8 Hz, H-6), 2.18 (dq, 7.0, 7.0 Hz, H-15), 2.12 (ddd, 7.8, 4.2, 3.8 Hz, H-2), 2.00 (app dd, 13.7, 6.2 Hz, H-9a), 1.80 (m, H-13a), 1.75 (m, H-16a), 1.70 (s, H_3_-24), 1.67 (s, H_3_-25), 1.66 (m, H_2_-12), 1.65 (m, H-4b), 1.58 (m, H-9b), 1.56 (m, H-1a), 1.45 (ddd, 12.6, 8.4, 3.0 Hz, H-13b), 1.41 (app d, 10.2 Hz, H-10), 1.33 (ddd, 11.7, 7.8, 3.0 Hz, H-16b), 1.26 (s, H_3_-20), 1.23 (m, H-1b), 0.98 (d, 7.1 Hz, H_3_-23), 0.91 (s, H_3_-22); ^13^C NMR δ: 142.0 (C-21, CH), 134.7 (C-19, C), 126.5 (C-18, CH), 109.6 (C-7, C), 96.6 (C-14, C), 86.3 (C-3, C), 81.9 (C-8, CH), 75.6 (C-5, CH), 70.8 (C-17, CH), 55.3 (C-10, CH), 55.0 (OCH_3_-8), 50.6 (C-4, CH_2_), 43.8 (C-6, CH), 42.6 (C-12, CH_2_), 42.5 (C-16, CH_2_), 38.3 (C-1, CH_2_), 36.9 (C-2, CH), 36.6 (C-15, CH), 29.1 (C-9, CH_2_), 28.9 (C-13, CH_2_), 25.9 (C-24, CH_3_), 21.1 (C-20, CH_3_), 18.8 (C-22, CH_3_), 18.2 (C-25, CH_3_), 16.8 (C-23, CH_3_). HR-ESI-MS *m*/*z* 439.2828 (calcd. for C_26_H_40_O_4_Na, 439.2824).

### Biological Testing

#### Cells

MCF7, MDA-MB-231, BT-20, SK-BR3, MDA-MB-468, T98G, and U87 GBM cells were obtained from ATCC. T98G cells were maintained in Eagle’s Minimum Essential Medium (Mediatech Inc., Corning, cat no. 10-010-CV, Manassas, VA) supplemental with 10% Fetalgro bovine growth serum (FBS, Rocky Mountain Biologicals, LLC, Missoula, MO) and 1% Penn/Strep (Mediatech, Inc., Corning). U87 were maintained in Dulbecco’s Modified Eagle Medium containing Ham’s F-12 50/50 mixture (88%, obtained from Mediatech, Inc., Corning), FBS (10%), Penn/Strep (1%), and nonessential amino acids (1%, obtained from Gibco, cat no. 11140-050, Waltham, MA). MCF7, MDA-MB-231, SK-BR3, and MDA-MB-468 cells were maintained in Dulbecco’s Modified Eagle’s Media (Corning, cat. no. 10-013-CM) supplemented with 10% Fetal bovine serum (ThermoFisherScientific, cat. no. A5256801) and 1% Penicillin–Streptomycin (ThermoFisherScientific, cat # 15140122). BT-20 cells were maintained in Minimum Essential Medium Eagle (MilliporeSigma, cat no. M4655) supplemented with 10% fetal bovine serum (ThermoFisherScientific, cat no. A5256801) and 1% Penicillin–Streptomycin (ThermoFisherScientific, cat # 15140122). HMLE and HMLE-TWIST cells were derived and cultured as previously described.^29^ The patient-derived primary glioblastoma stem cells (GSCs), GSC 040815 and GSC 082209, were developed and cultured as previously described.^33,37^ The cells were maintained as neurospheres in neurobasal medium (Invitrogen, Carlsbad, CA), which was supplemented with B27 serum-free supplement, 1% penicillin–streptomycin, 1% sodium pyruvate, EGF (20 ng/mL), bFGF (20 ng/mL), LIF (10 ng/mL), and heparin (5 μg/mL).

#### Cell Viability Assays

The GBM cells were prepared by trypsinizing each cell line and seeding 1 × 10^4^ cells per well into microtiter 96-well plates. All compounds were dissolved in DMSO and diluted in 1% DMSO in full media at concentrations ranging from 1 mM to 0.1 nM in 10-fold increments prior to cell treatment. The cells were grown for 24 h in their respective media before the media was removed and replaced with 100 μL of media plus the respective drug concentration. After 48 h, the medium was removed and replaced with 100 μL of MTT reagent in phenol red- and serum-free medium (0.5 mg/mL) and incubated further for 2 h. Media was removed, and the resulting formazan crystals were resolubilized in 100 μL of DMSO. A_550_ (peak) and A_700_ (Baseline) were measured using a BioTek Synergy H4 plate reader. The baseline absorbance was subtracted from all peak absorbances to obtain the viability. Cells treated with 1% DMSO were used as a control.

The mammary and breast cancer cells were prepared by trypsinizing each cell line and seeding 2 × 10^3^ cells per well into microtiter 96-well plates. All compounds were dissolved in DMSO and diluted in full media at concentrations ranging from 0.1 mM to 100 nM in 4-fold increments prior to cell treatment. The cells were grown for 24 h in their respective media before the media was removed and replaced with 100 μL of media plus the respective drug concentration or equivalently diluted DMSO in duplicate wells. After 72 h, the medium was removed and replaced with 80 μL of medium plus 20 μL of CellTiterBlue reagent and incubated further for 3 h. Fluorescence was detected using Ex_560_ (excitation) and Em_590_ (emission) wavelengths using a Varioskan LUX Multimode Microplate Reader plate reader (Thermo Scientific). The baseline fluorescence was subtracted from all wells to obtain viability, and treated wells were normalized to the average of the signal from wells with volume-equivalent DMSO.

The effect of compounds on GSC viability was assessed using the CellTiter-Glo 2.0 Cell viability assay (Promega catalog no. G9241, Madison, WI) following the manufacturer’s protocol. GSCs (4 × 10^3^ cells) were treated for 7 days with either a vehicle (DMSO 0.1% v/v), OpA (**4**), or new analogs at specified doses. Luminescence levels were measured by using a Promega Glomax Luminometer.

#### Statistical Analysis

Six replicates were used per condition. IC_50_ values and standard deviations were calculated on GraphPad Prism v9 using (log) inhibitor vs normalized response—variable slope analysis using a four parameter mode.

## Supplementary Material











## Notes

1D and 2D NMR spectra of new gigobolins A–C (**1**–**3**), and of maydispenoid A (**9**), HRESIMS spectra for gigobolins A–C and maydispenoid A (**9**), ECD curves for gigobolin B (**2**) and maydispenoid A (**9**) (PDF)
Gigobolin A_NMR_RAW_DATA (ZIP)
Gigobolin B_NMR_RAW_DATA (ZIP)
Gigobolin C_NMR_RAW_DATA (ZIP)
Maydispenoid A_NMR_RAW_DATA (ZIP)
