# Supplementary material for: Gigobolins A–C, New Ophiobolins with Anticancer Activity from the Phytopathogenic Fungus Drechslera gigantea
Source: J Nat Prod. 2026 Feb 27;89(3):864–72. doi: 10.1021/acs.jnatprod.5c01414 (PMC13036769; doi:10.1021/acs.jnatprod.5c01414)
Supplement: Supplementary file 1 [file np5c01414_si_001.pdf]

Supporting Information

Gigobolins A-C, New Ophiobolins with Anticancer Activity from the Phytopathogenic Fungus *Drechslera gigantea*

Marianna Carbone,[1,a] Sridharan Jayamohan,[1,b] Amelia Scott,[1,c] Angela Boari,[d] Ryan J. Rutledge,[c] Nadia Cacciapuoti,[a] Vamika Gautam,[e] Jaquelin Aroujo,[f] Tania Betancourt,[c] Daniel Romo,[f] Gangadhara Reddy Sareddy,[b] Joseph H. Taube,[e] Alexander Kornienko,[c] Antonio Evidente[a] and Maria Letizia Ciavatta[a,*]

[a]*National Research Council (CNR), Institute of Biomolecular Chemistry, Via Campi Flegrei 34, 80078 Pozzuoli, Italy*
[b]*Department of Obstetrics and Gynecology, 7703 Floyd Curl Drive, University of Texas Health San Antonio, San Antonio, TX 78229, USA*
[c]*Department of Biology, Baylor University, 101 Bagby Ave., Waco, TX 76798, United States*
[d]*Department of Chemistry and Biochemistry, Texas State University, 601 University Dr., TX 78666, United States*
[e]*Institute of Sciences of Food Production, National Research Council, Via Amendola 122/O, 70125 Bari, Italy*
[f]*Department of Chemistry and Biochemistry, Baylor University, 101 Bagby Ave., Waco, TX 76798, United States*

[*] To whom correspondence should be addressed: marialetizia.ciavatta@cnr.it

[1] These authors contributed equally

Table of Contents

S1.	^{1}H NMR spectrum of gigobolin A (**1**) (Bruker 600 MHZ, $CDCl_3$)	pag.1
S2.	^{13}C NMR spectrum of gigobolin A (**1**) (Bruker 600 MHZ, $CDCl_3$)	pag.2
S3.	COSY spectrum of gigobolin A (**1**) (Bruker 600 MHZ, $CDCl_3$)	pag.3
S4.	*ed*-HSQC spectrum of gigobolin A (**1**) (Bruker 400 MHZ, $CDCl_3$)	pag.4
S5.	HMBC spectrum of gigobolin A (**1**) (Bruker 600 MHZ, $CDCl_3$, $J = 7$ Hz)	pag.5
S6.	NOESY spectrum of gigobolin A (**1**) (Bruker 600 MHZ, $CDCl_3$)	pag.6
S7	Upfield region expansion of the NOESY spectrum of gigobolin A (**1**) (Bruker 600 MHZ, $CDCl_3$)	pag.7
S8.	HRESI MS spectrum of gigobolin A (**1**)	pag.8
S9.	^{1}H NMR spectrum of gigobolin B (**2**) (Bruker 600 MHZ, $CDCl_3$)	pag.9
S10.	^{13}C NMR spectrum of gigobolin B (**2**) (Bruker 600 MHZ, $CDCl_3$)	pag.10
S11.	COSY spectrum of gigobolin B (**2**) (Bruker 600 MHZ, $CDCl_3$)	pag.11
S12.	*ed*-HSQC spectrum of gigobolin B (**2**) (Bruker 400 MHZ, $CDCl_3$)	pag.12
S13.	HMBC spectrum of gigobolin B (**2**) (Bruker 600 MHZ, $CDCl_3$, $J = 7$ Hz)	pag.13
S14.	NOESY spectrum of gigobolin B (**2**) (Bruker 600 MHZ, $CDCl_3$)	pag.14
S15.	Upfield region expansion of the NOESY spectrum of gigobolin B (**2**) (Bruker 600 MHZ, $CDCl_3$)	pag.15
S16.	HRESI MS spectrum of gigobolin B (**2**)	pag.16
S17.	^{1}H NMR spectrum of gigobolin C (**3**) (Bruker 600 MHZ, $CDCl_3$)	pag.17
S18.	^{13}C NMR spectrum of gigobolin C (**3**) (Bruker 600 MHZ, $CDCl_3$)	pag.18
S19.	COSY spectrum of gigobolin C (**3**) (Bruker 400 MHZ, $CDCl_3$)	pag.19
S20.	*ed*-HSQC spectrum of gigobolin C (**3**) (Bruker 400 MHZ, $CDCl_3$)	pag.20
S21.	HMBC spectrum of gigobolin C (**3**) (Bruker 600 MHZ, $CDCl_3$, $J = 7$ Hz)	pag.21
S22.	HRESI MS spectrum of gigobolin C (**3**)	pag.22
S23.	^{1}H NMR spectrum of maydispenoid A (**9**) (Bruker 600 MHZ, $CDCl_3$)	pag.23
S24.	^{13}C NMR spectrum of maydispenoid A (**9**) (Bruker 400 MHZ, $CDCl_3$)	pag.24
S25.	COSY spectrum of maydispenoid A (**9**) (Bruker 600 MHZ, $CDCl_3$)	pag.25
S26.	*ed*-HSQC spectrum of maydispenoid A (**9**) (Bruker 400 MHZ, $CDCl_3$)	pag.26
S27.	HMBC spectrum of maydispenoid A (**9**) (Bruker 600 MHZ, $CDCl_3$, $J = 7$ Hz)	pag.27
S28.	NOESY spectrum of maydispenoid A (**9**) (Bruker 600 MHZ, $CDCl_3$)	pag.28
S29.	Upfield region expansion of the NOESY spectrum of maydispenoid A (**9**) (Bruker 600 MHZ, $CDCl_3$)	pag.29
S30.	HRESI MS spectrum of maydispenoid A (**9**)	pag.30
S31.	ECD spectra of gigobolin B (**2**) and maydispenoid A (**9**)	pag.31

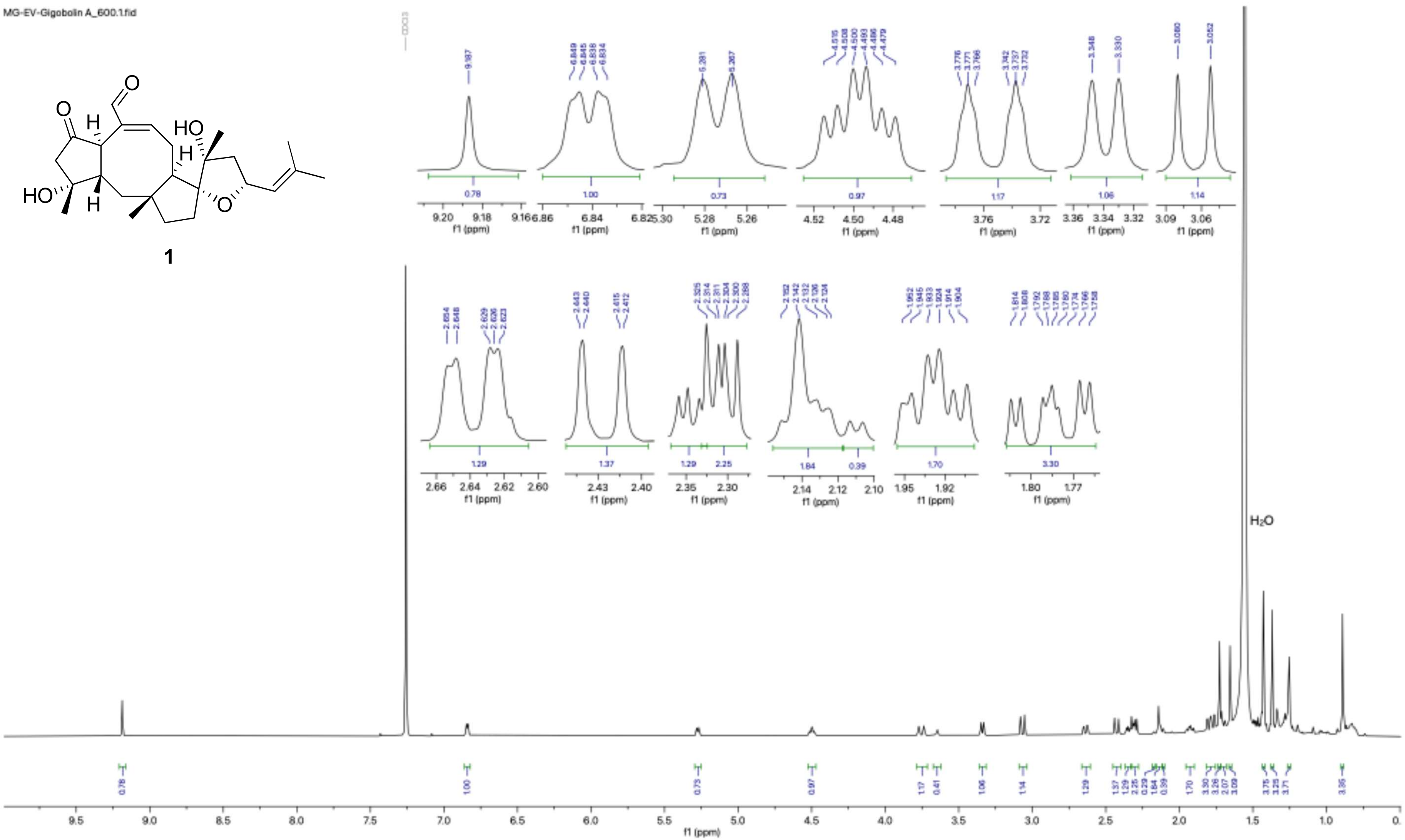

^{1}H NMR spectrum of gigobolin A (**1**) (Bruker 600 MHZ, $CDCl_3$)

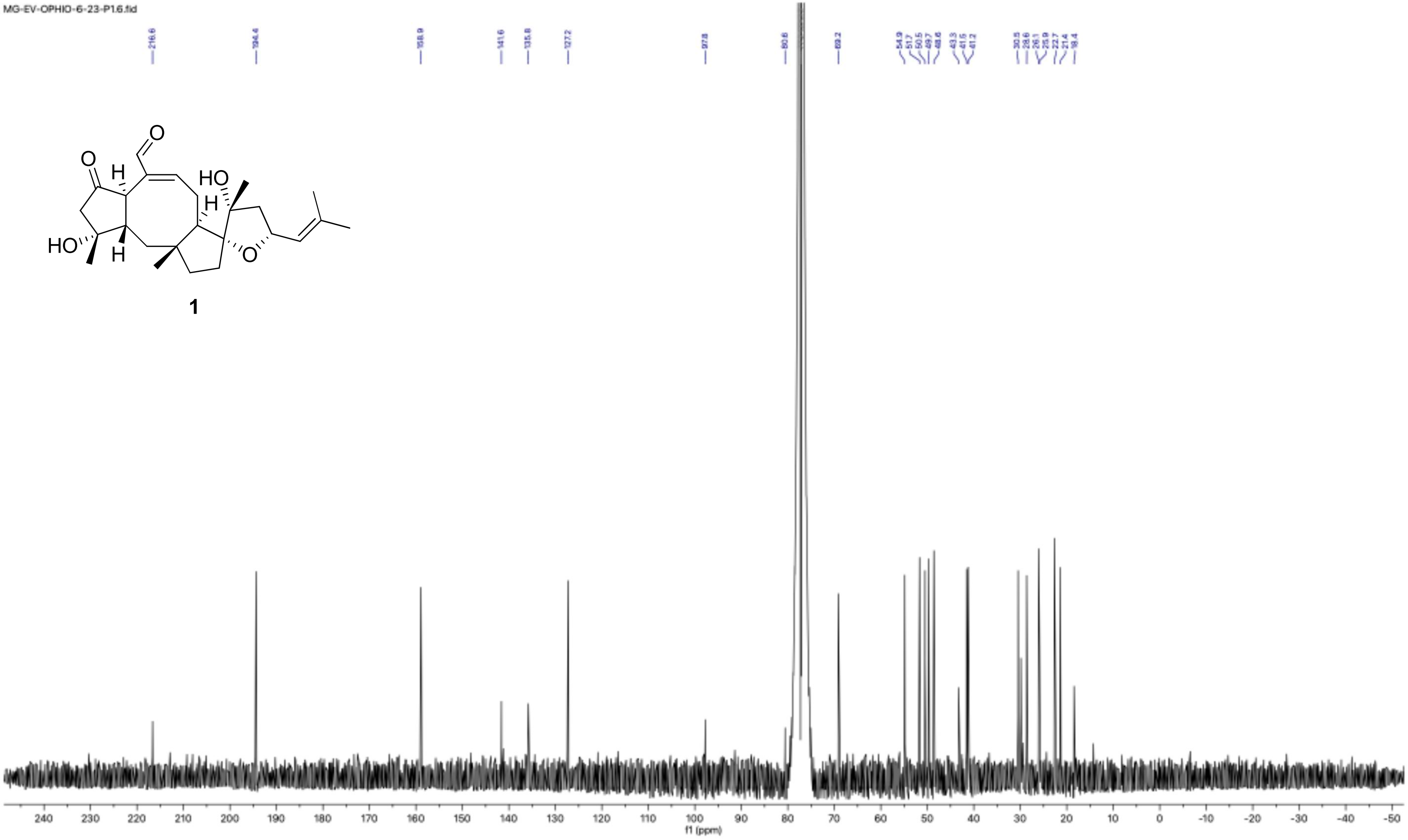

^{13}C NMR spectrum of gigobolin A (**1**) (Bruker 600 MHZ, $CDCl_3$)

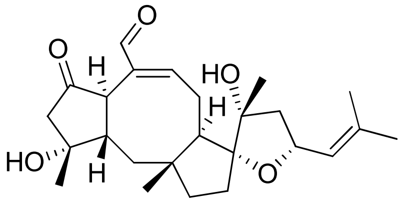

1

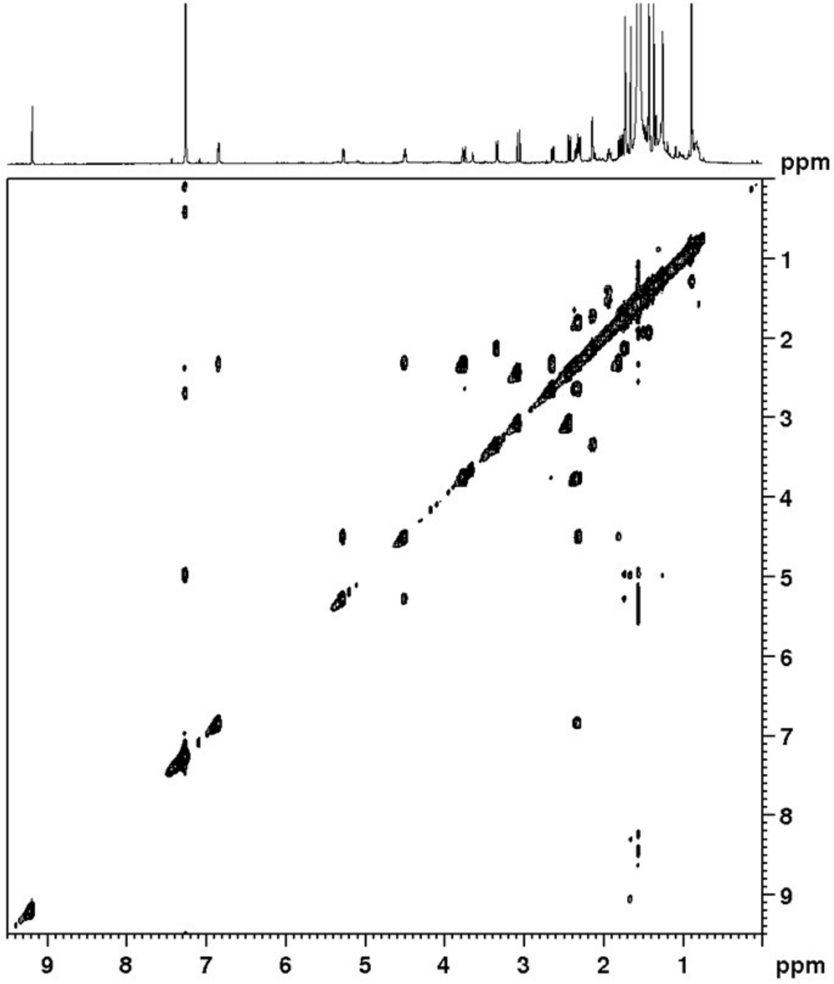

COSY spectrum of gigobolin A (**1**) (Bruker 600 MHZ, $CDCl_3$)

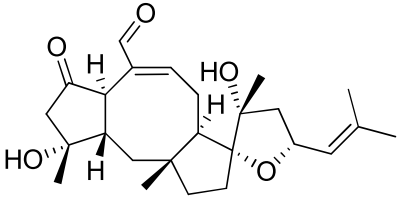

1

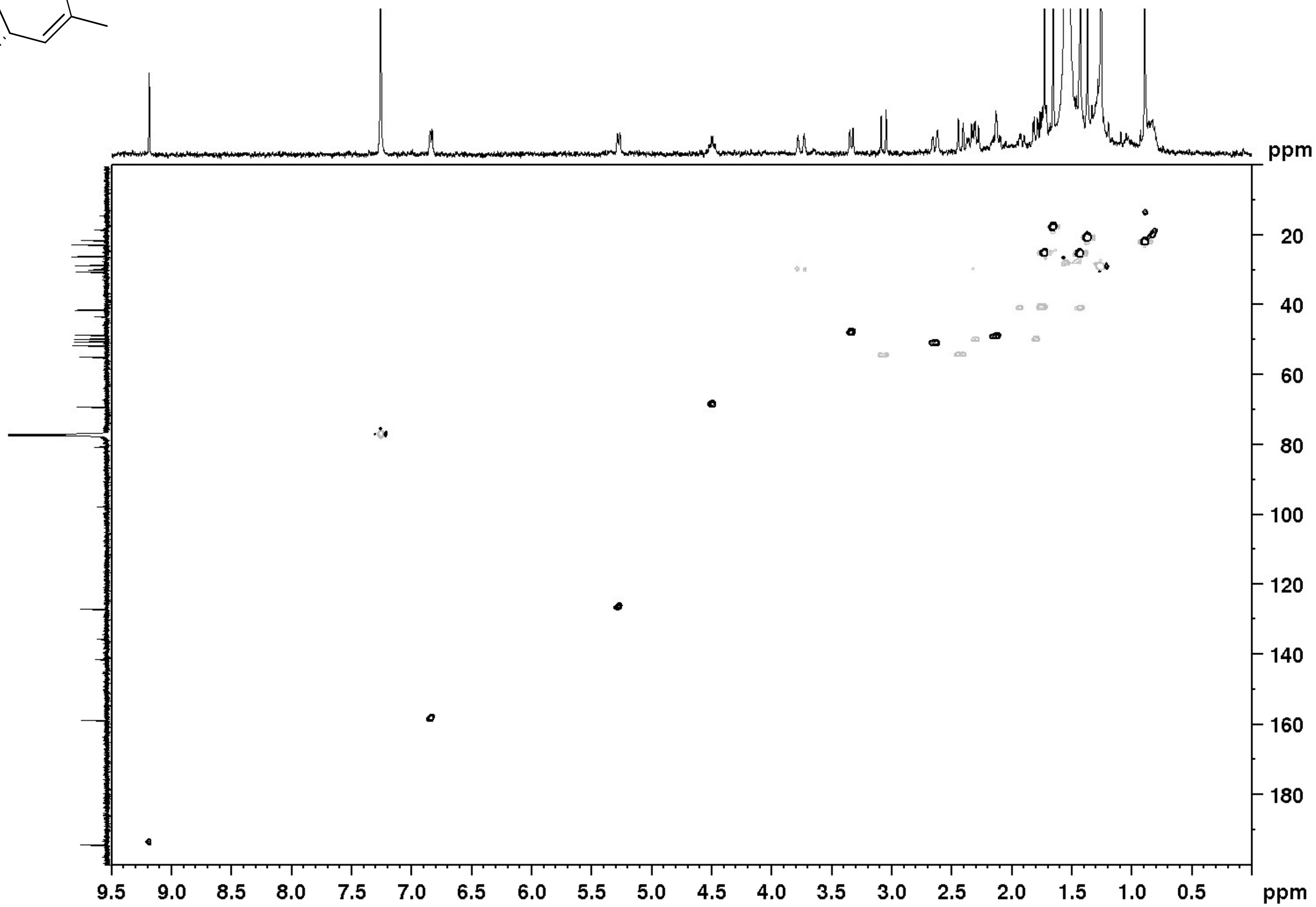

ed-HSQC spectrum of gigobolin A (**1**) (Bruker 400 MHZ, $CDCl_3$)

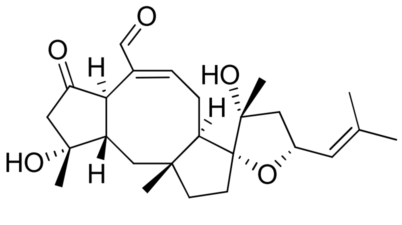

1

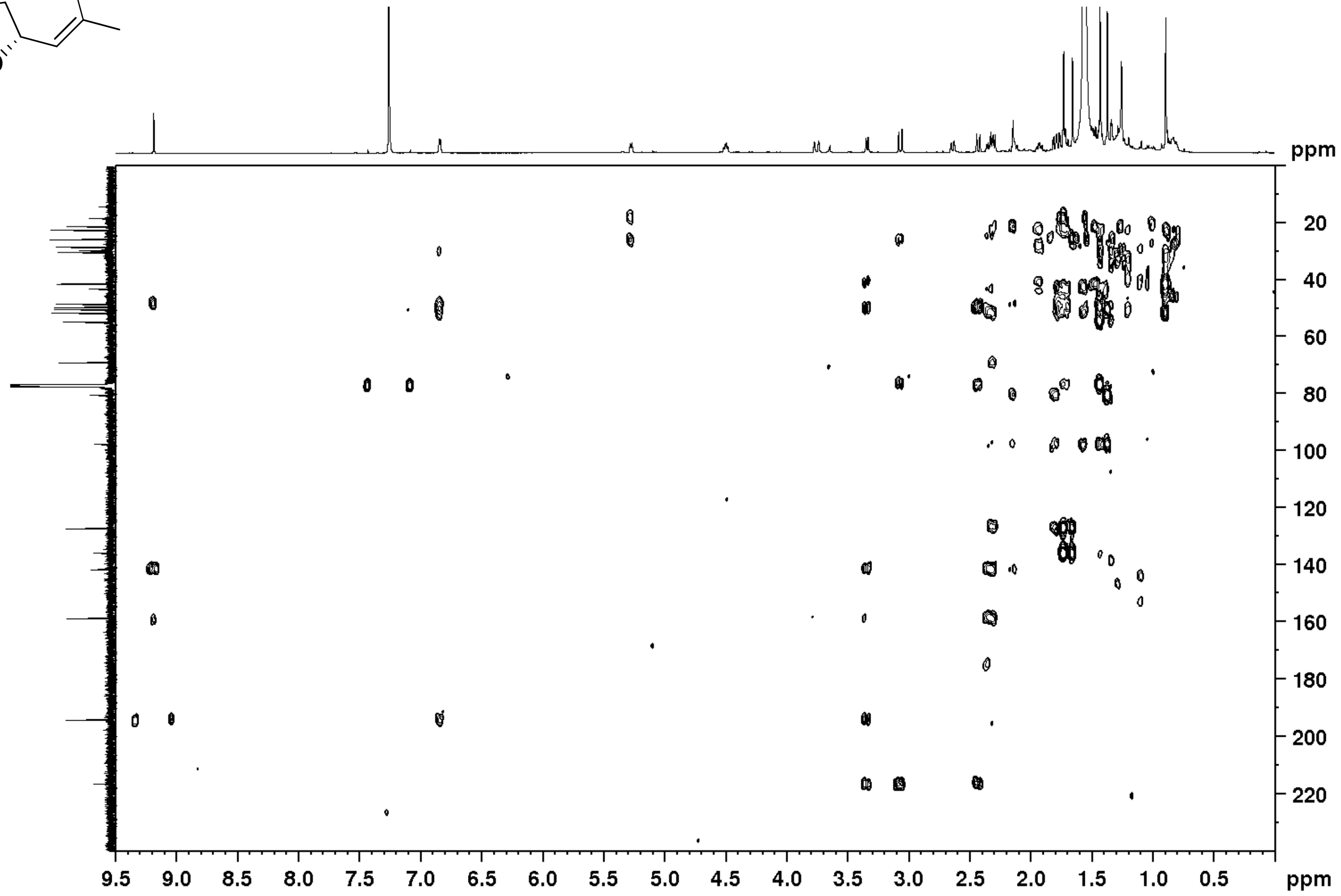

HMBC spectrum of gigobolin A (**1**) (Bruker 600 MHZ, $CDCl_3$, J = 7 Hz)

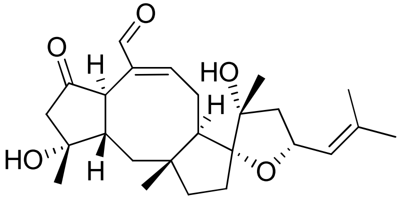

1

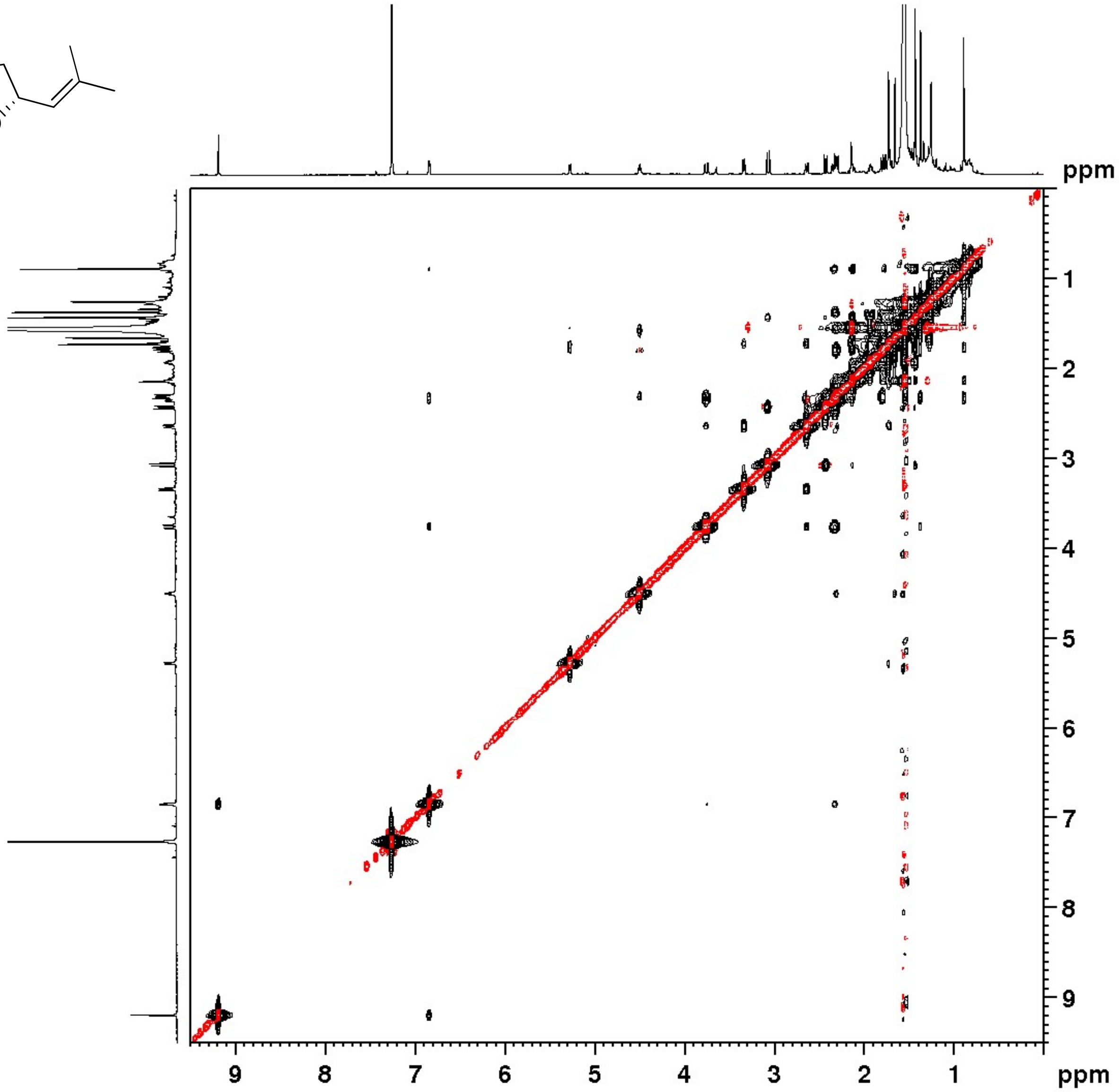

NOESY spectrum of gigobolin A (**1**) (Bruker 600 MHZ, $CDCl_3$)

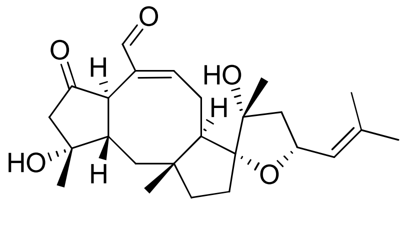

1

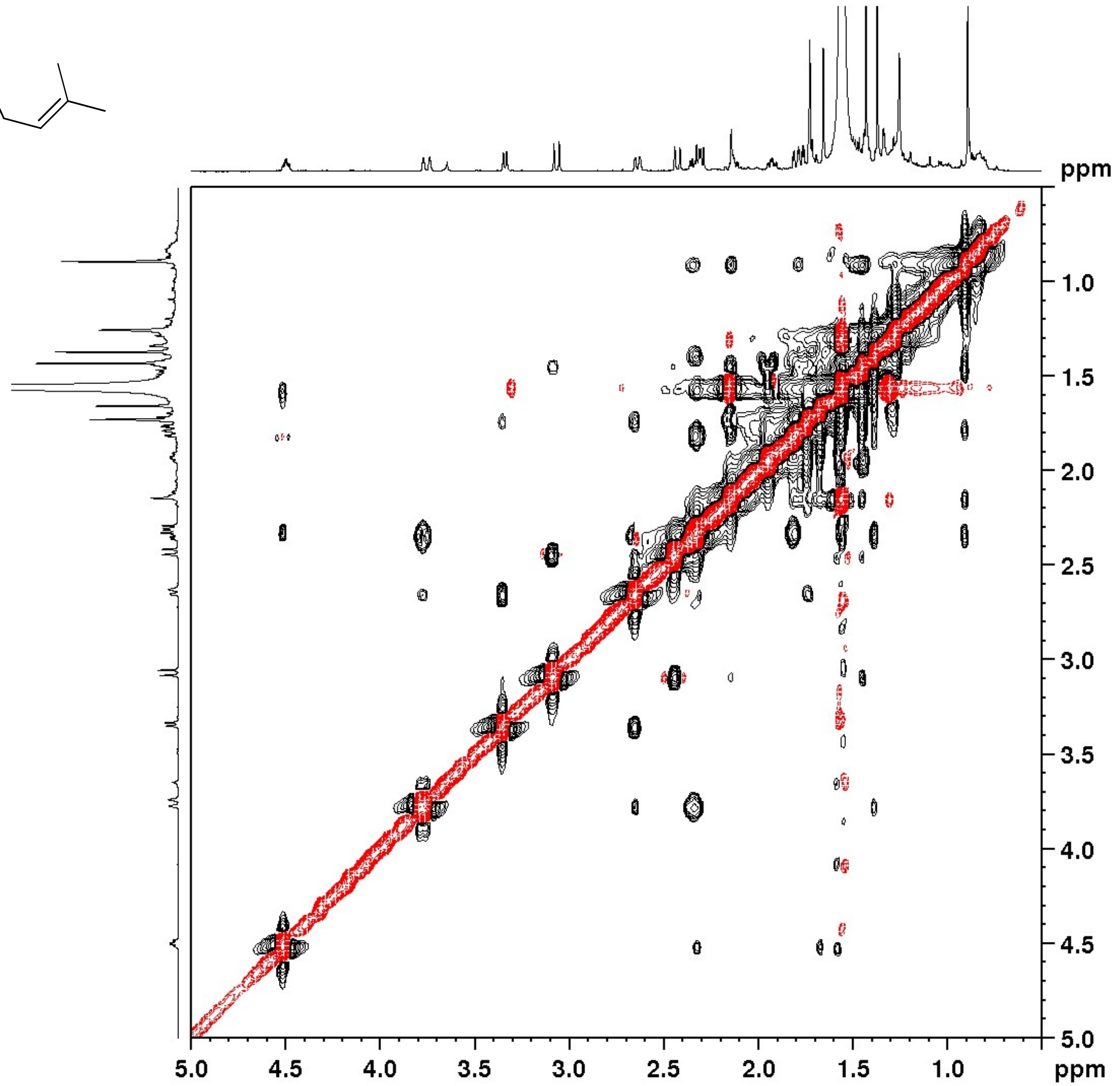

Upfield region expansion of the NOESY spectrum of gigobolin A (**1**) (Bruker 600 MHZ, $CDCl_3$)

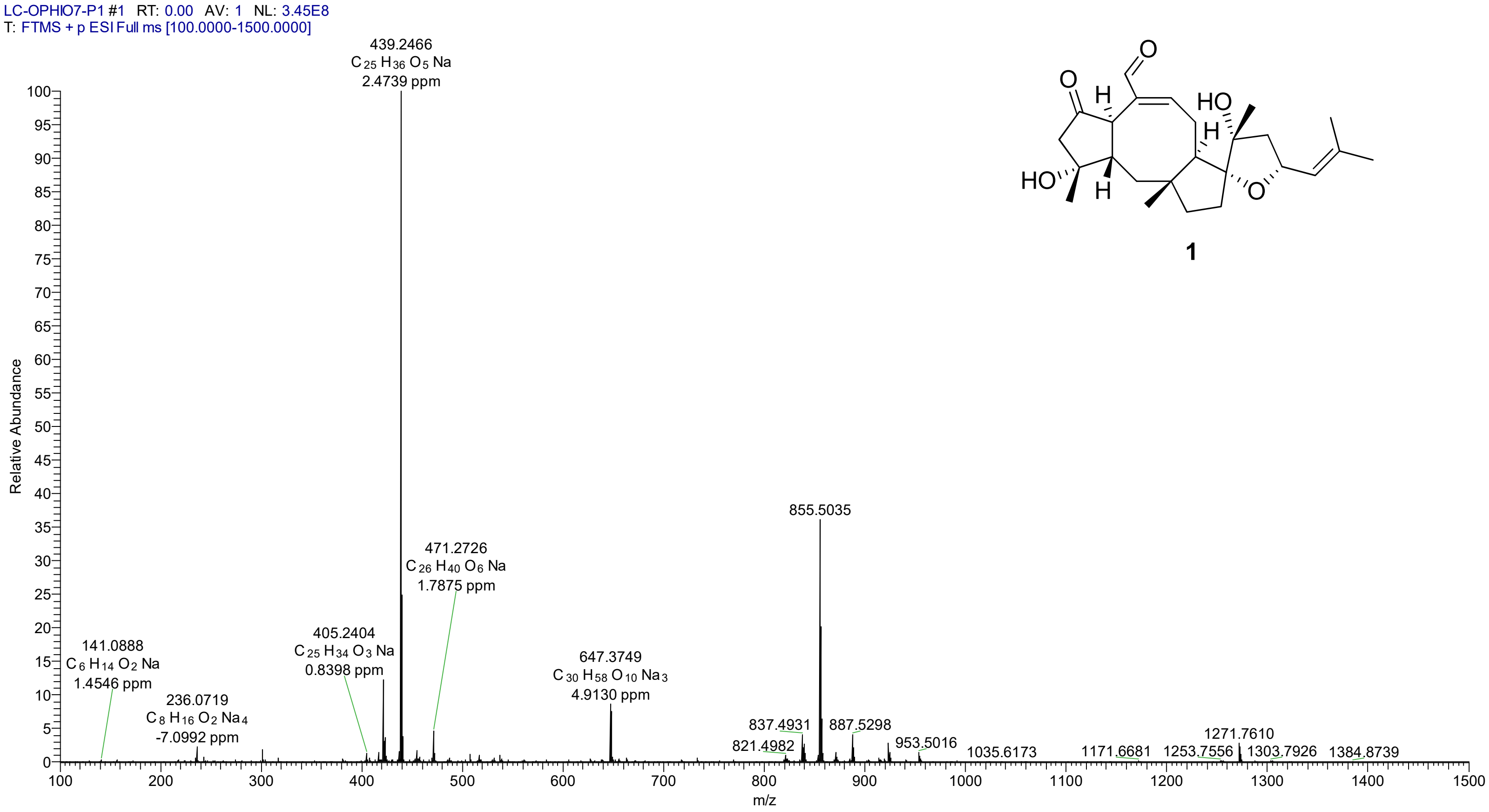

HRESI MS spectrum of gigobolin A (**1**)

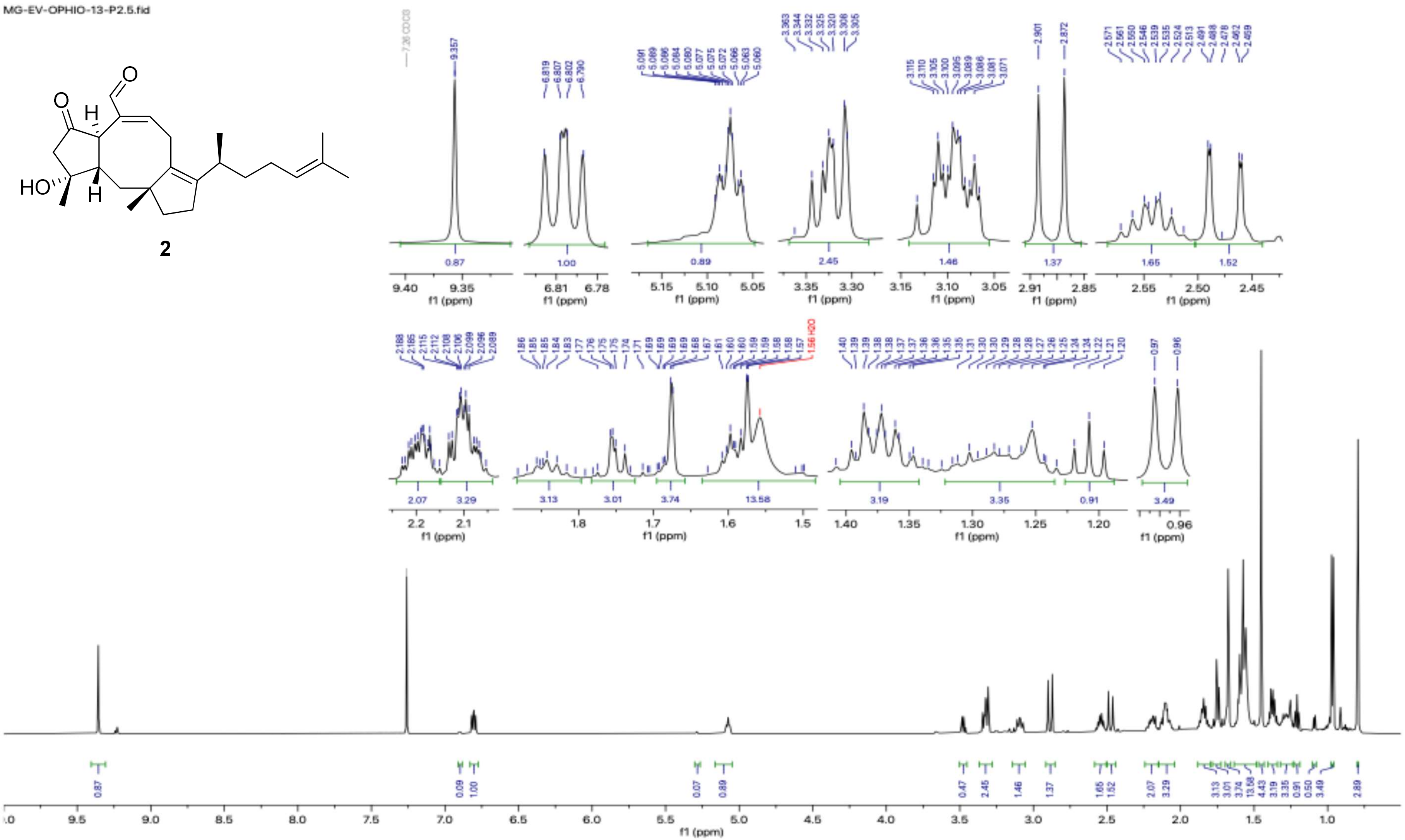

^{1}H NMR spectrum of gigobolin B (**2**) (Bruker 600 MHZ, $CDCl_3$)

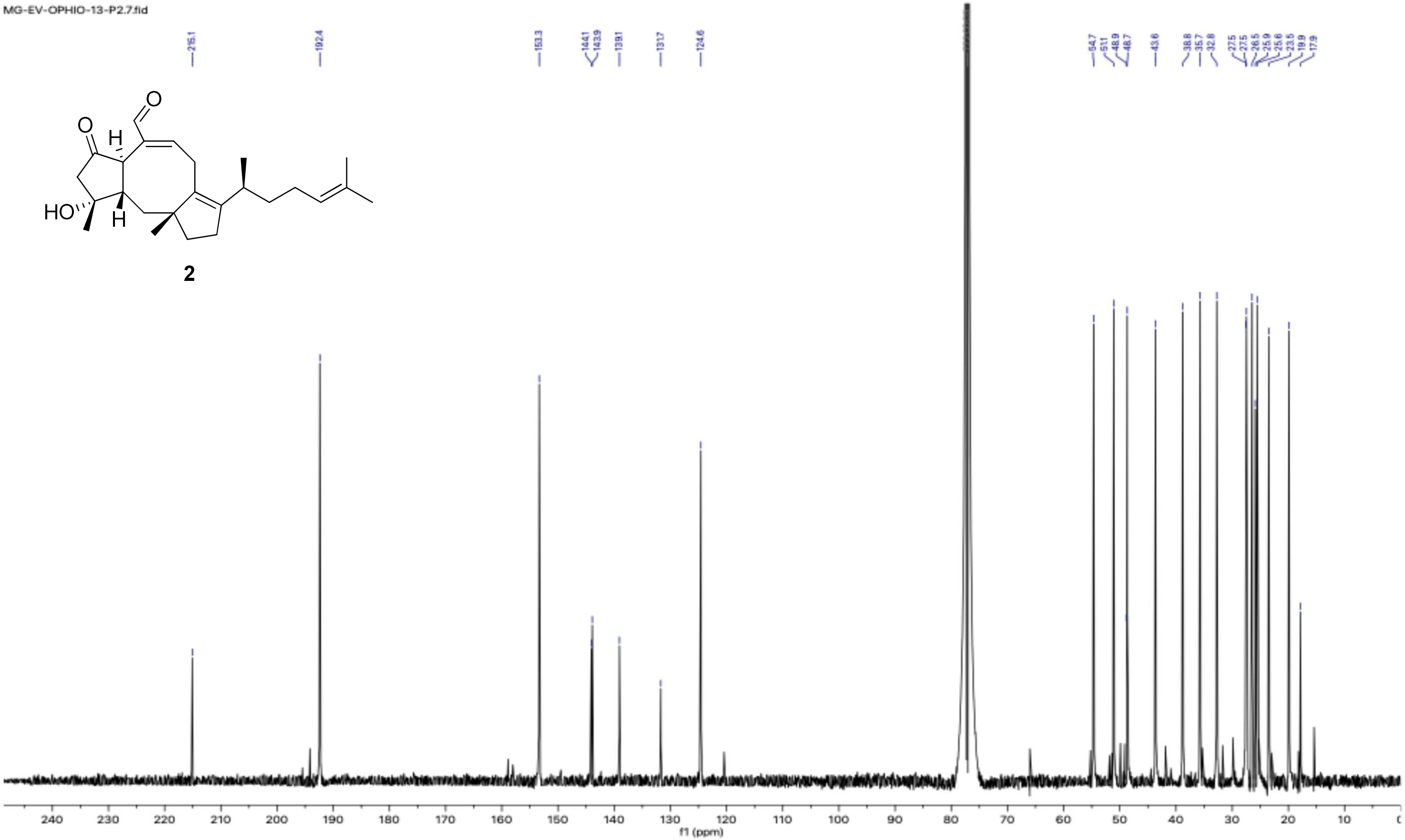

^{13}C NMR spectrum of gigobolin B (**2**) (Bruker 600 MHZ, $CDCl_3$)

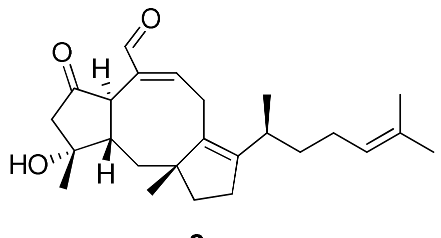

2

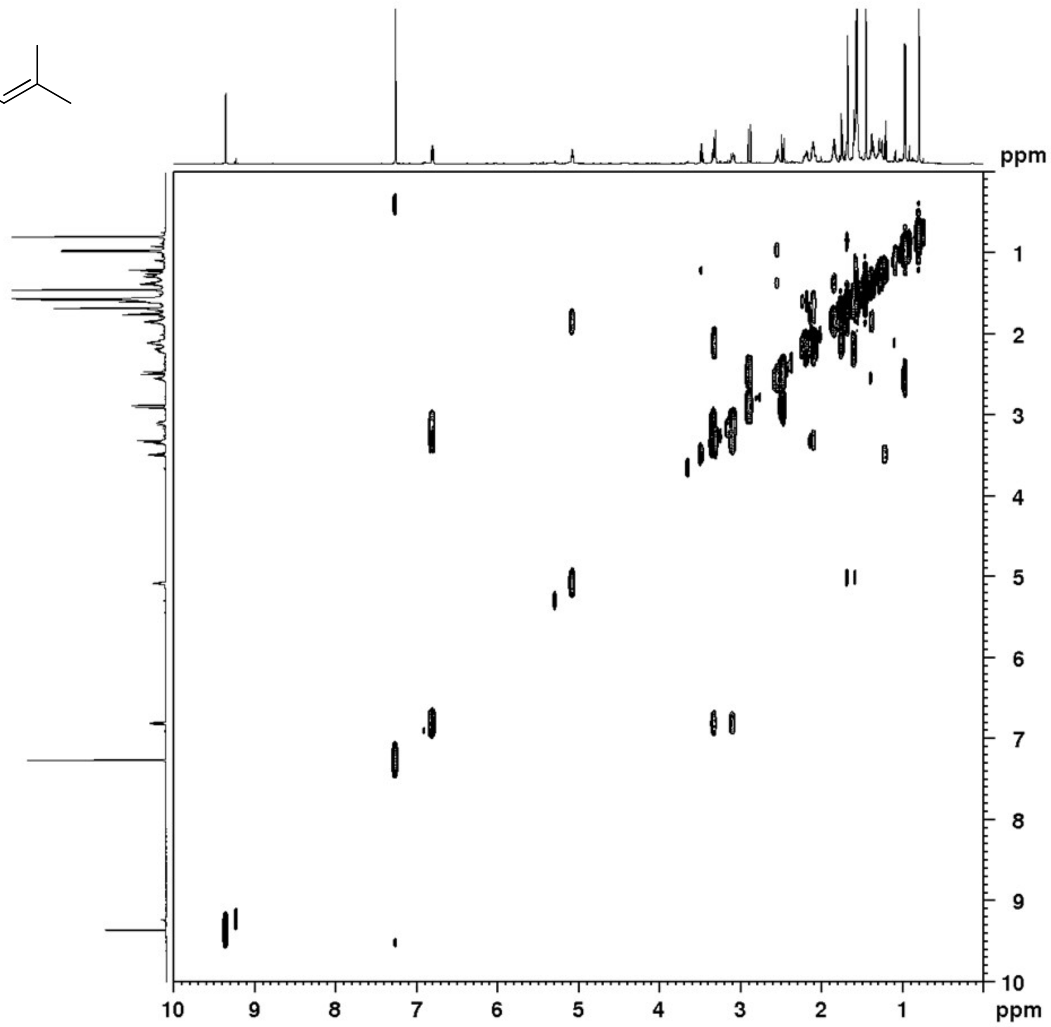

COSY spectrum of gigobolin B (**2**) (Bruker 600 MHZ, $CDCl_3$)

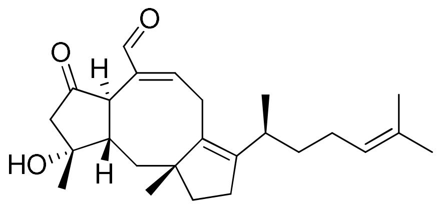

2

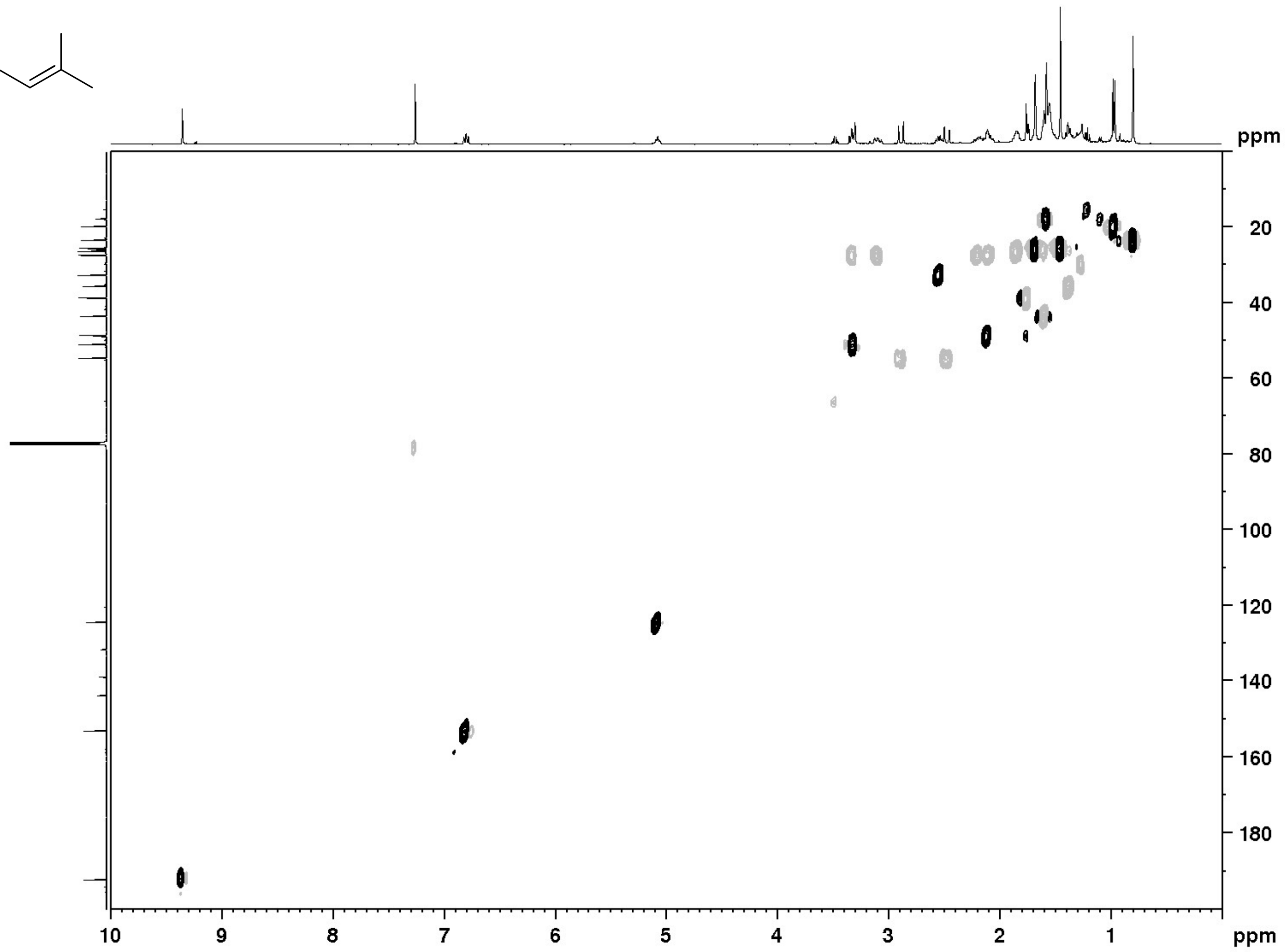

ed-HSQC spectrum of gigobolin B (**2**) (Bruker 400 MHZ, $CDCl_3$)

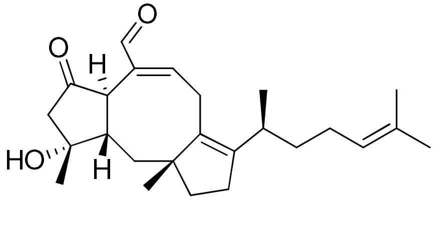

2

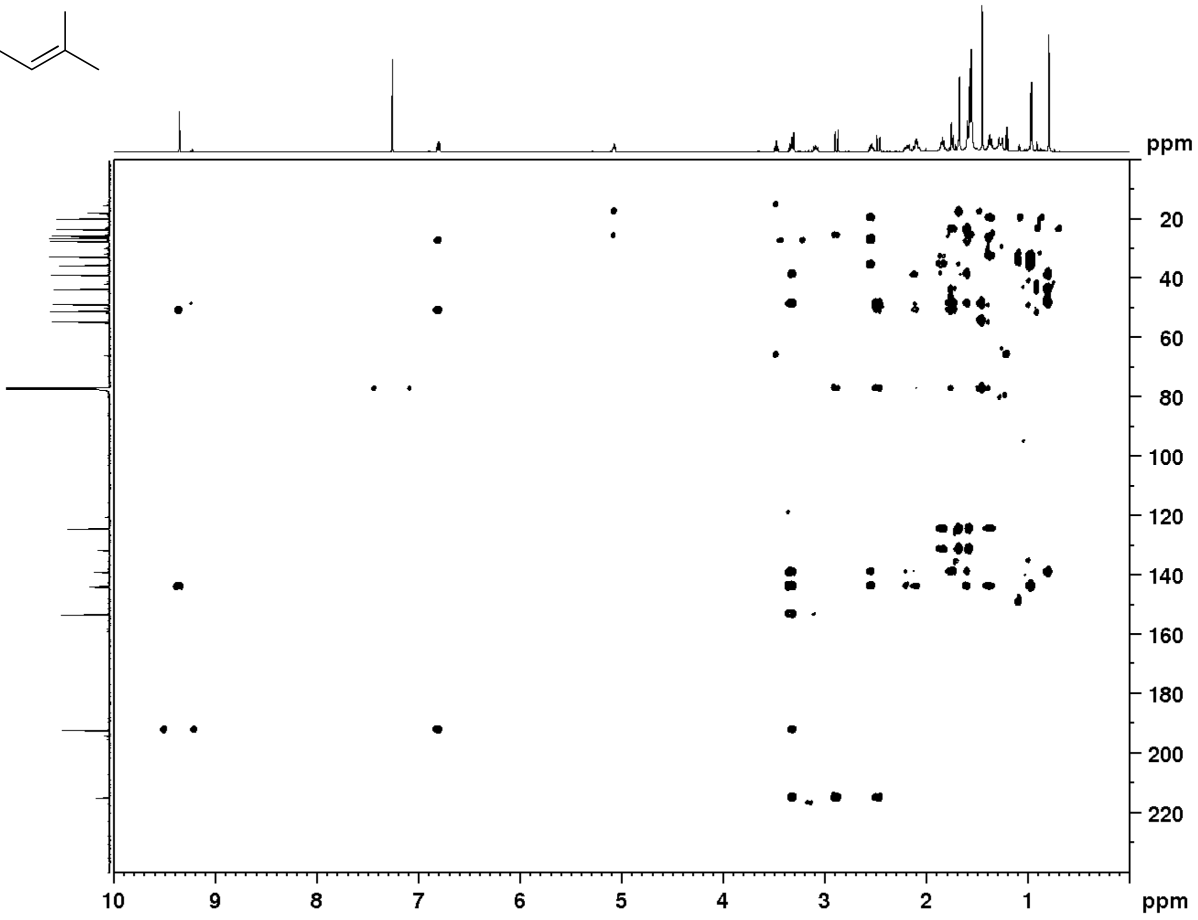

HMBC spectrum of gigobolin B (**2**) (Bruker 600 MHZ, $CDCl_3$, *J* = 7 Hz)

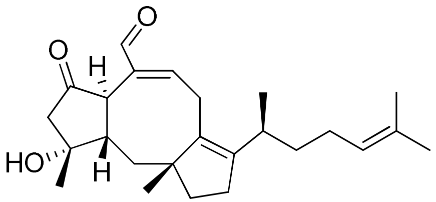

2

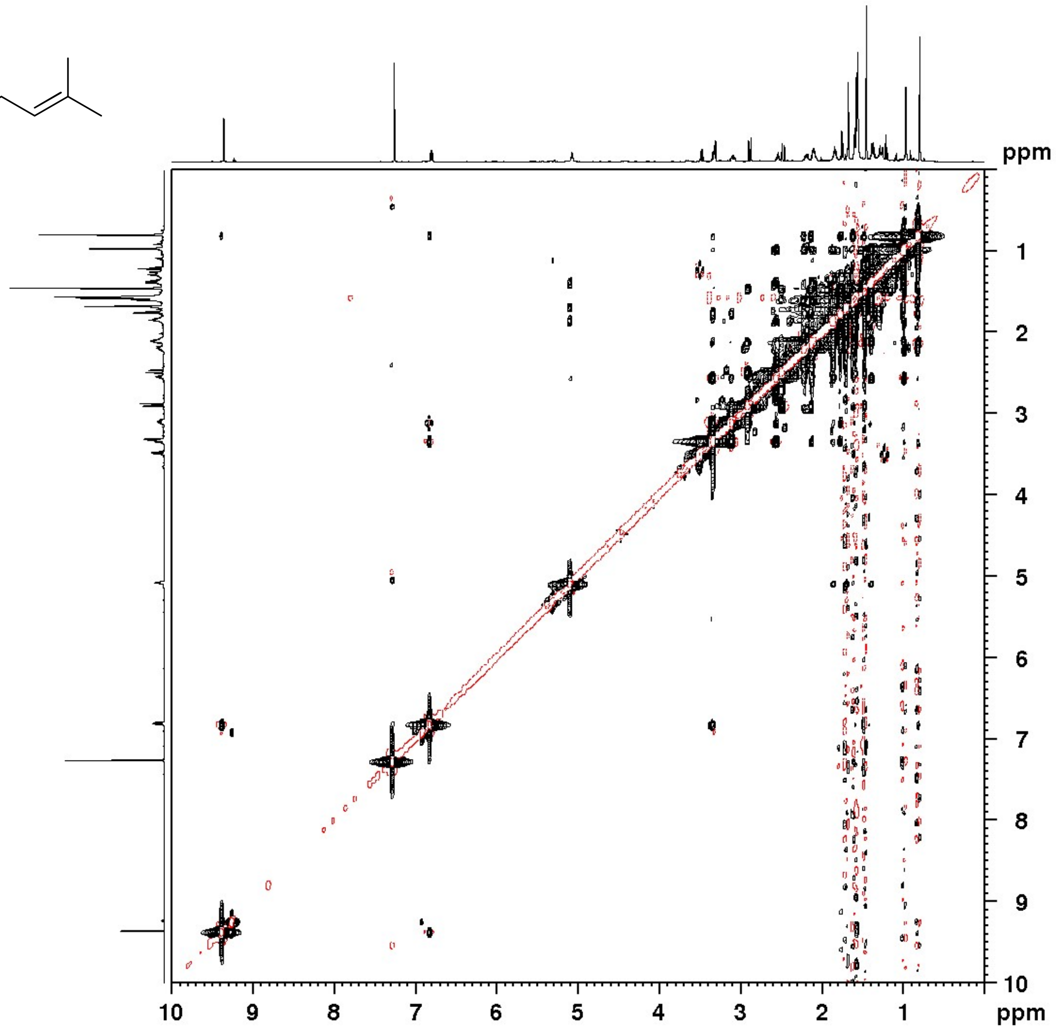

NOESY spectrum of gigobolin B (**2**) (Bruker 600 MHZ, $CDCl_3$)

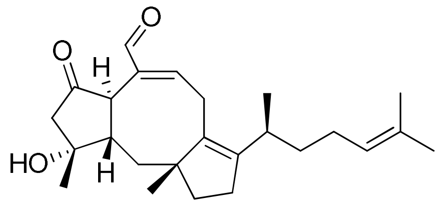

2

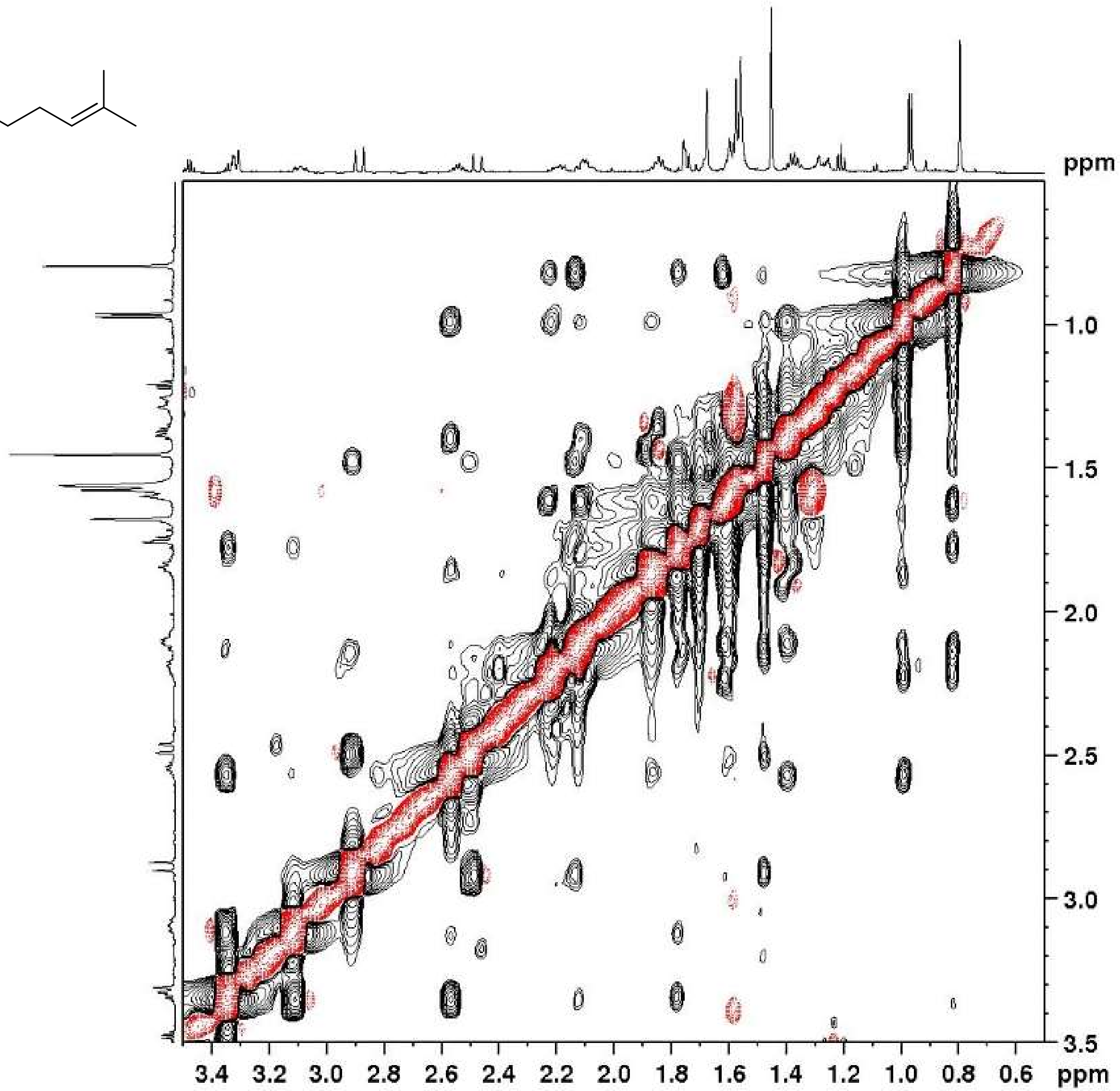

Upfield region expansion of the NOESY spectrum of gigobolin B (**2**) (Bruker 600 MHZ, $CDCl_3$)

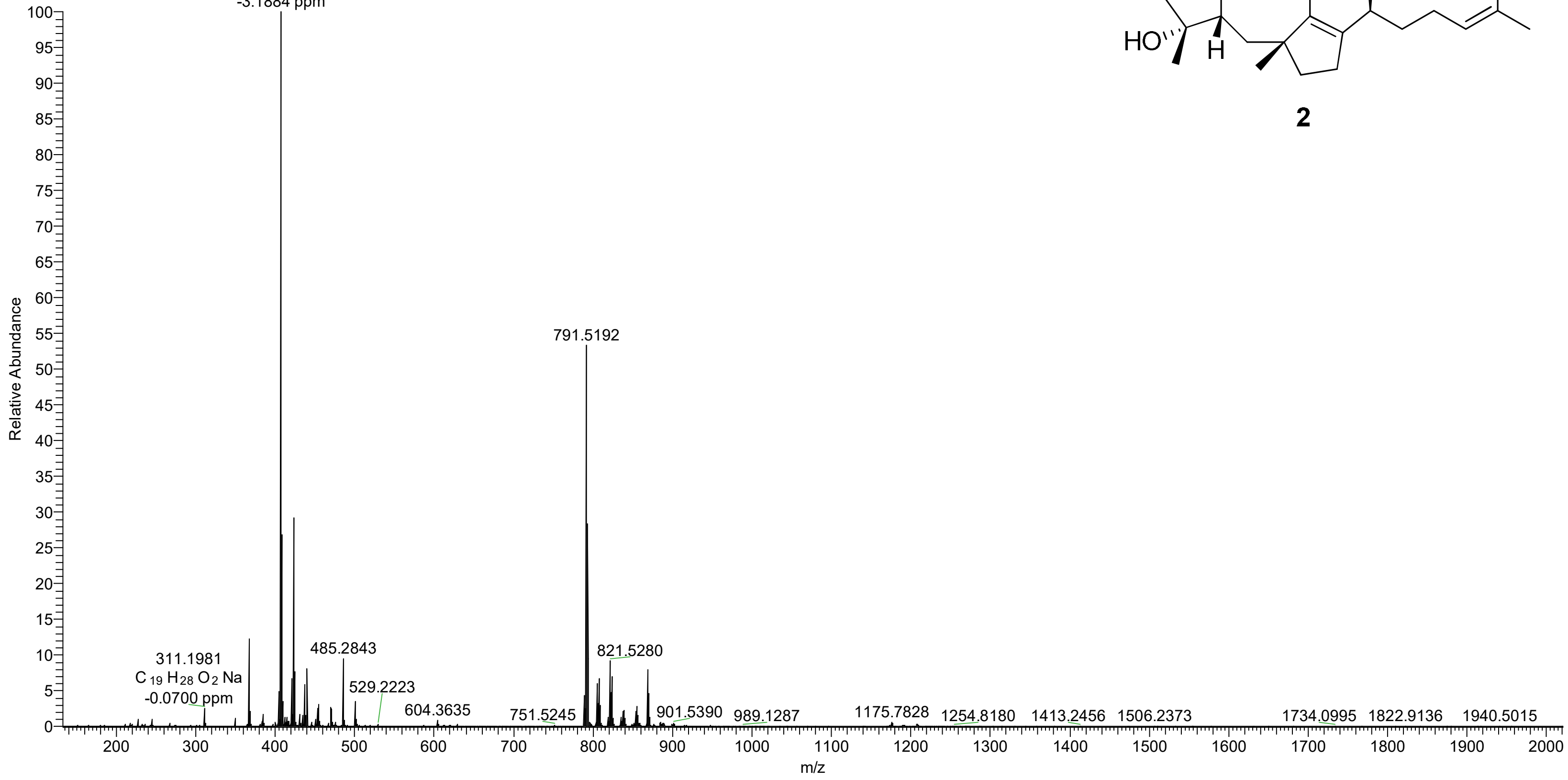

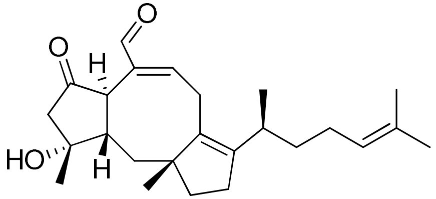

2

HRESI MS spectrum of gigobolin B (**2**)

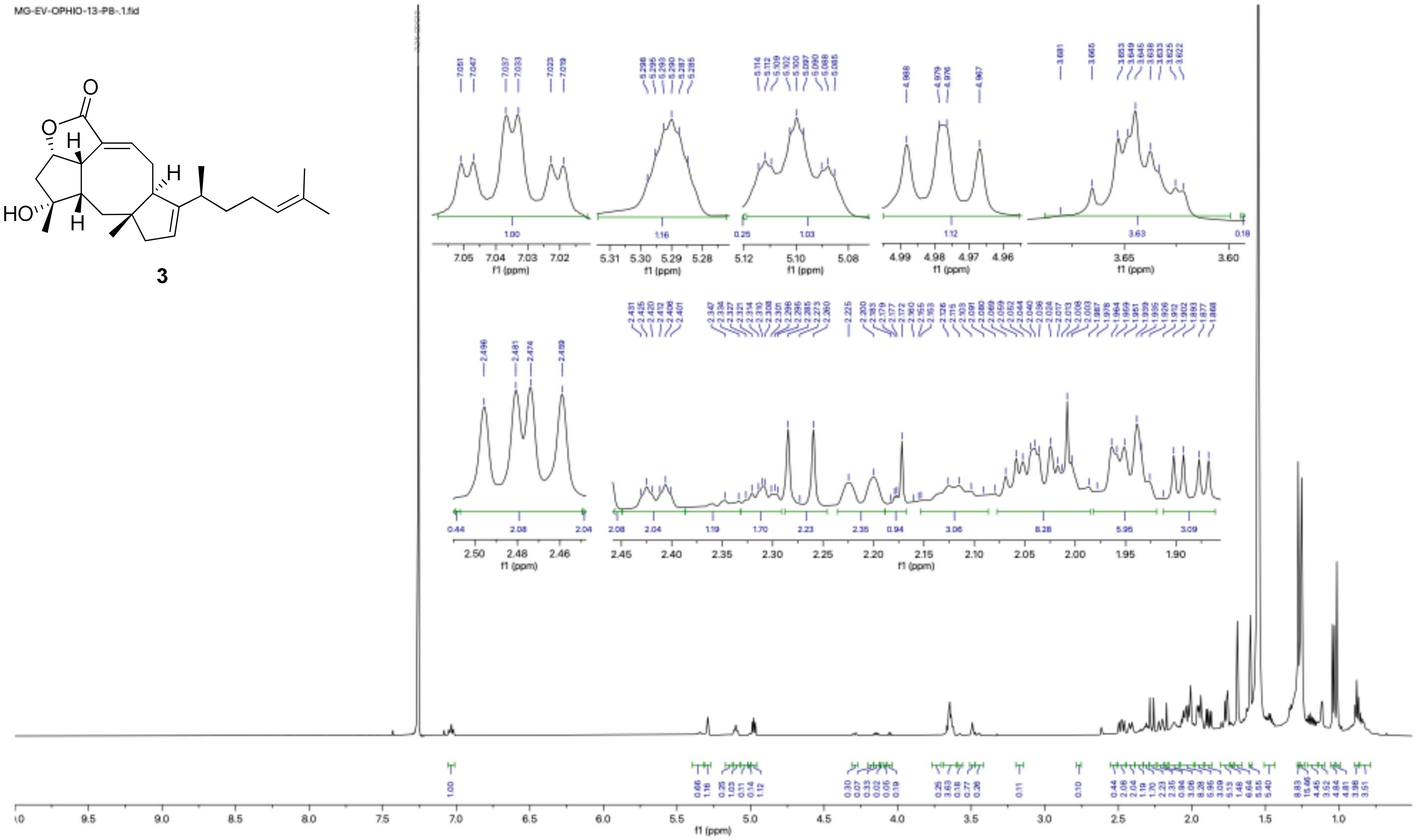

^{1}H NMR spectrum of gigobolin C (**3**) (Bruker 600 MHZ, $CDCl_3$)

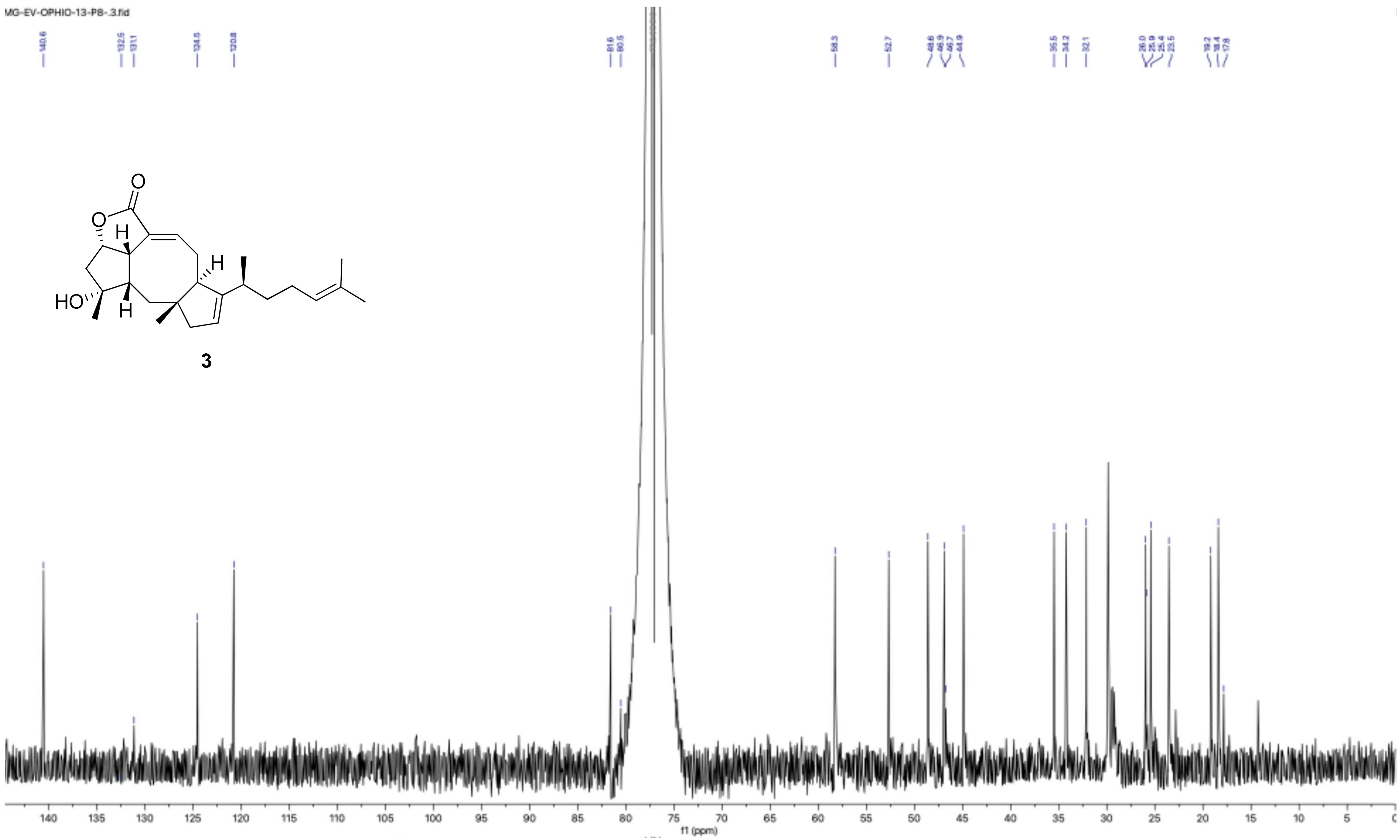

^{13}C NMR spectrum of gigobolin C (**3**) (Bruker 600 MHZ, $CDCl_3$)

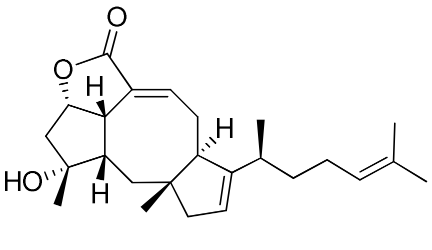

3

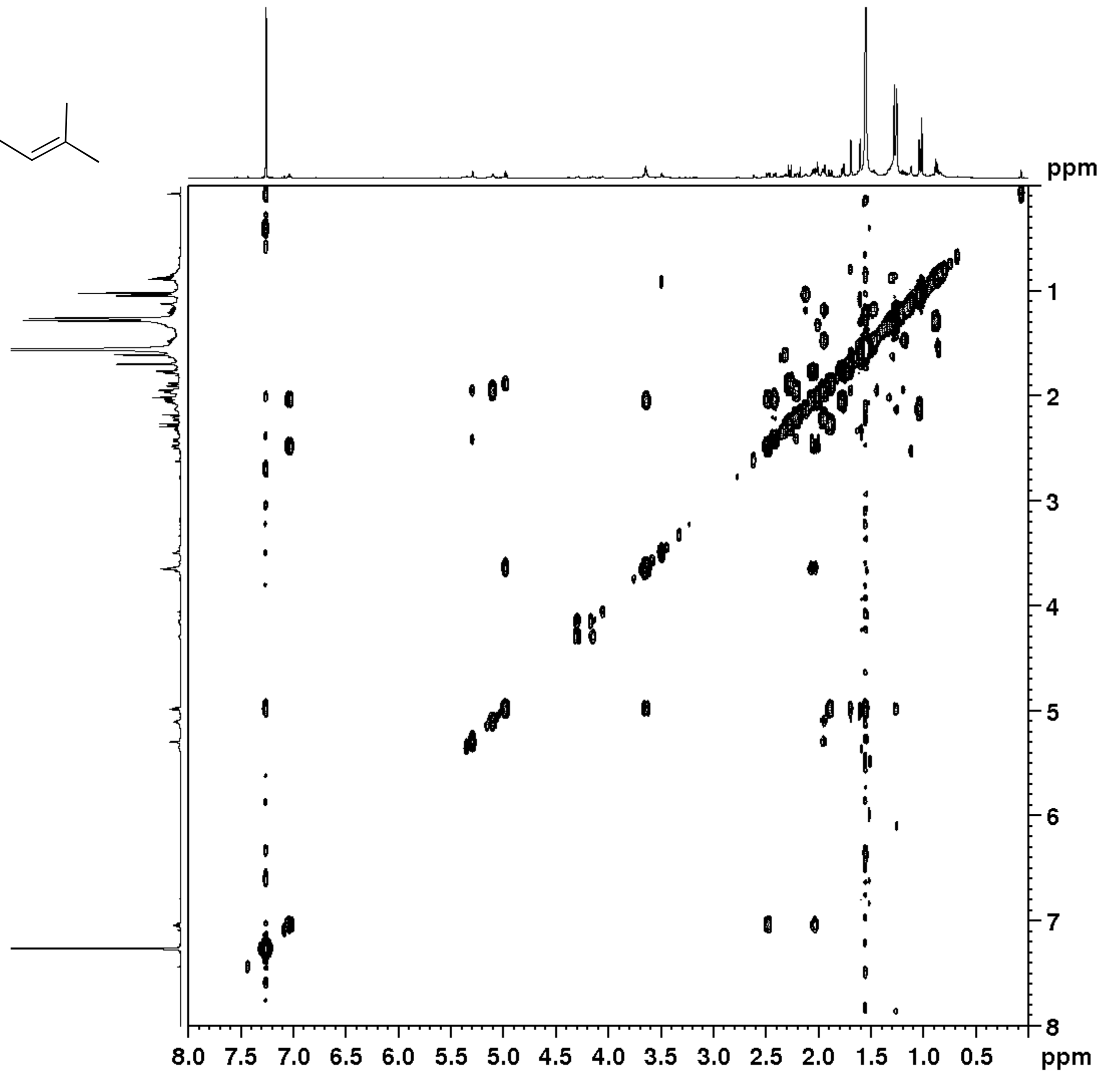

COSY spectrum of gigobolin C (**3**) (Bruker 400 MHZ, $CDCl_3$)

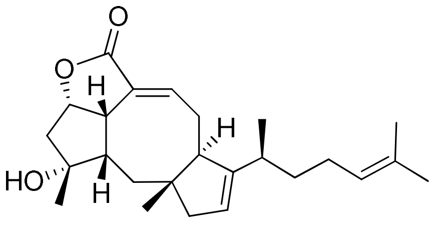

3

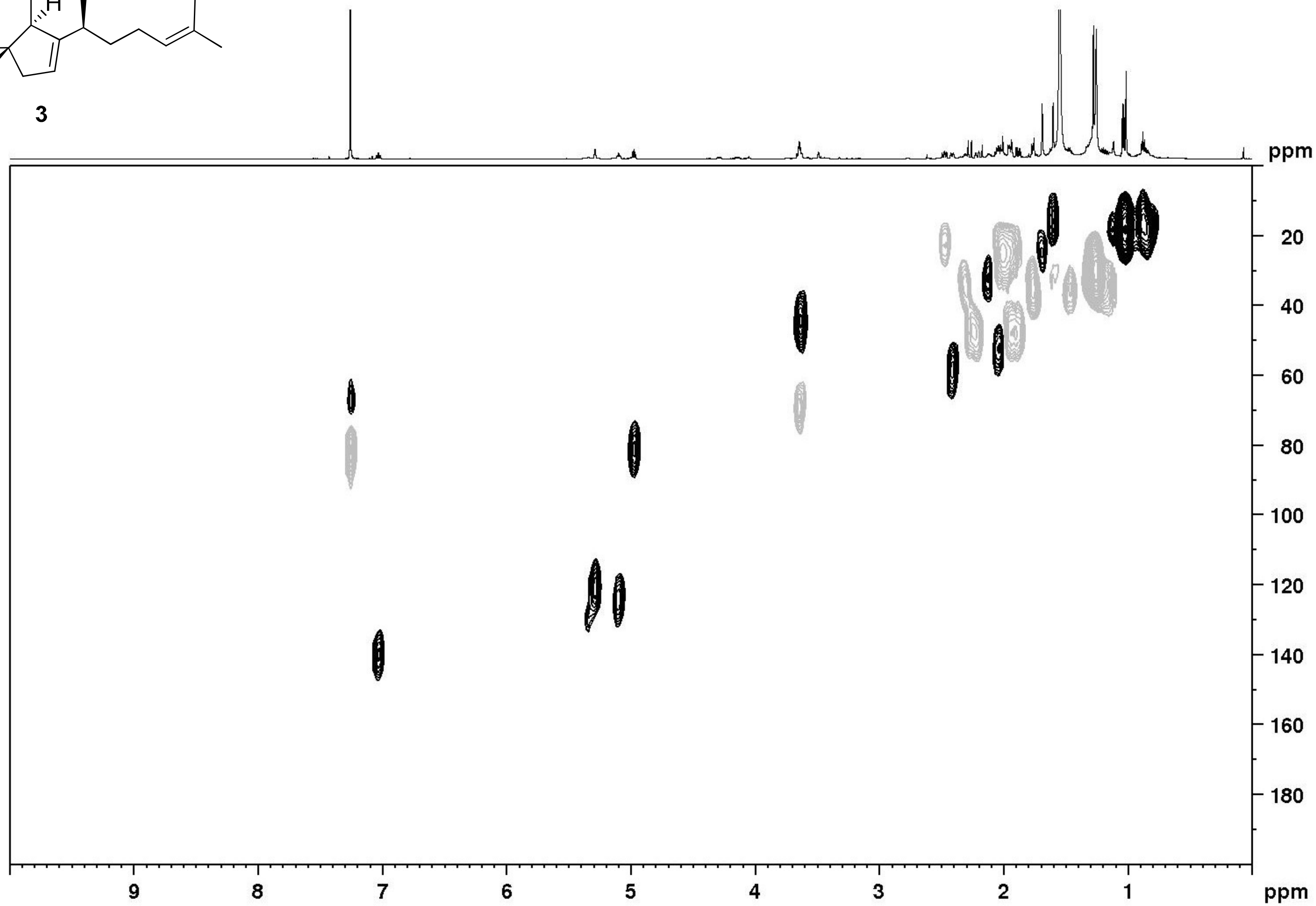

ed-HSQC spectrum of gigobolin C (**3**) (Bruker 400 MHZ, $CDCl_3$)

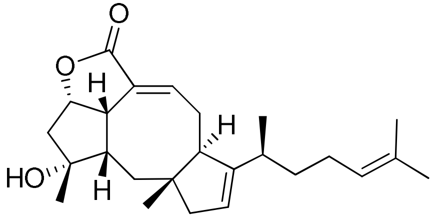

3

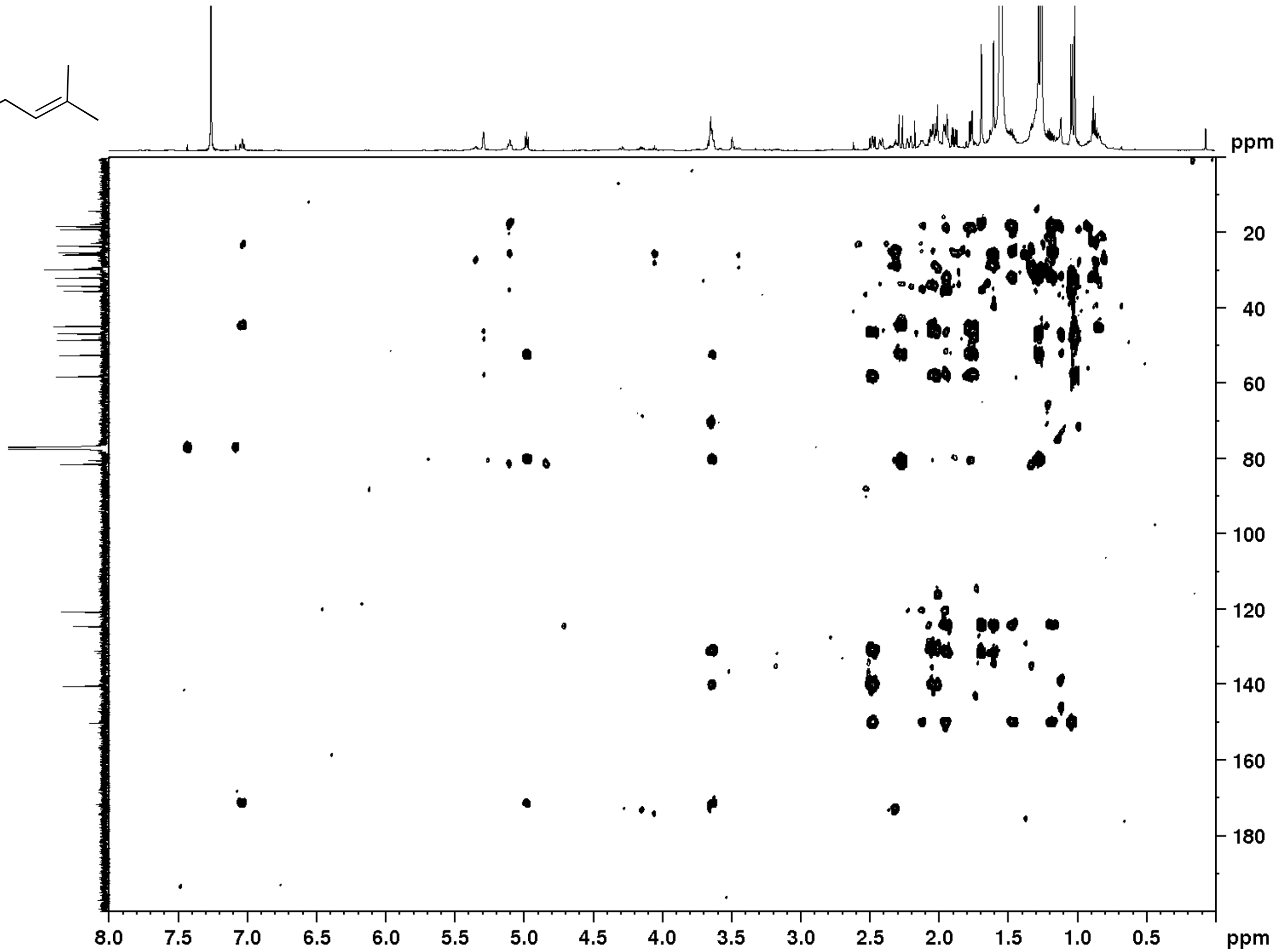

HMBC spectrum of gigobolin C (**3**) (Bruker 600 MHZ, $CDCl_3$, J = 7 Hz)

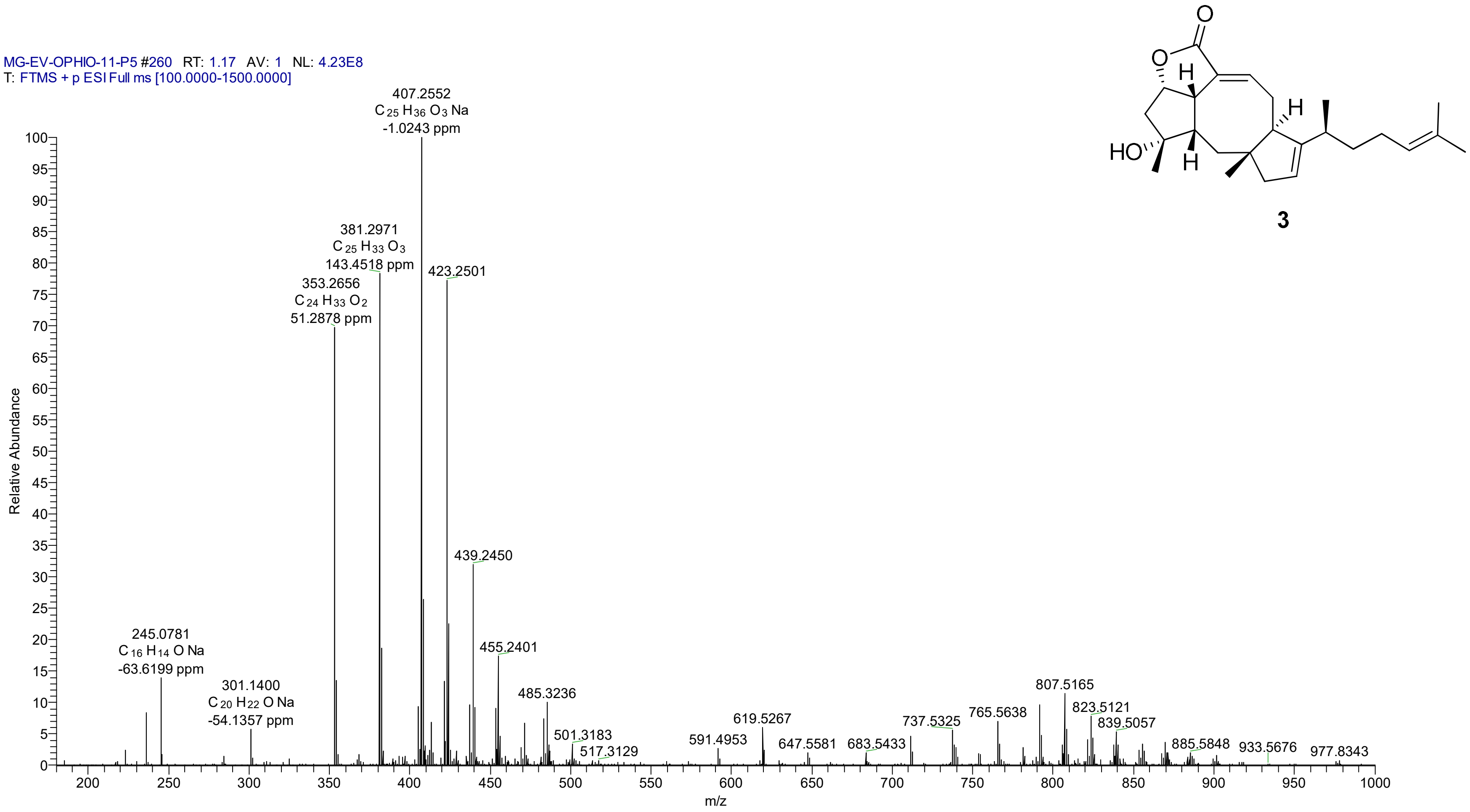

HRESI MS spectrum of gigobolin C (**3**)

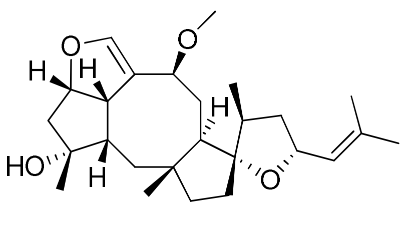

9

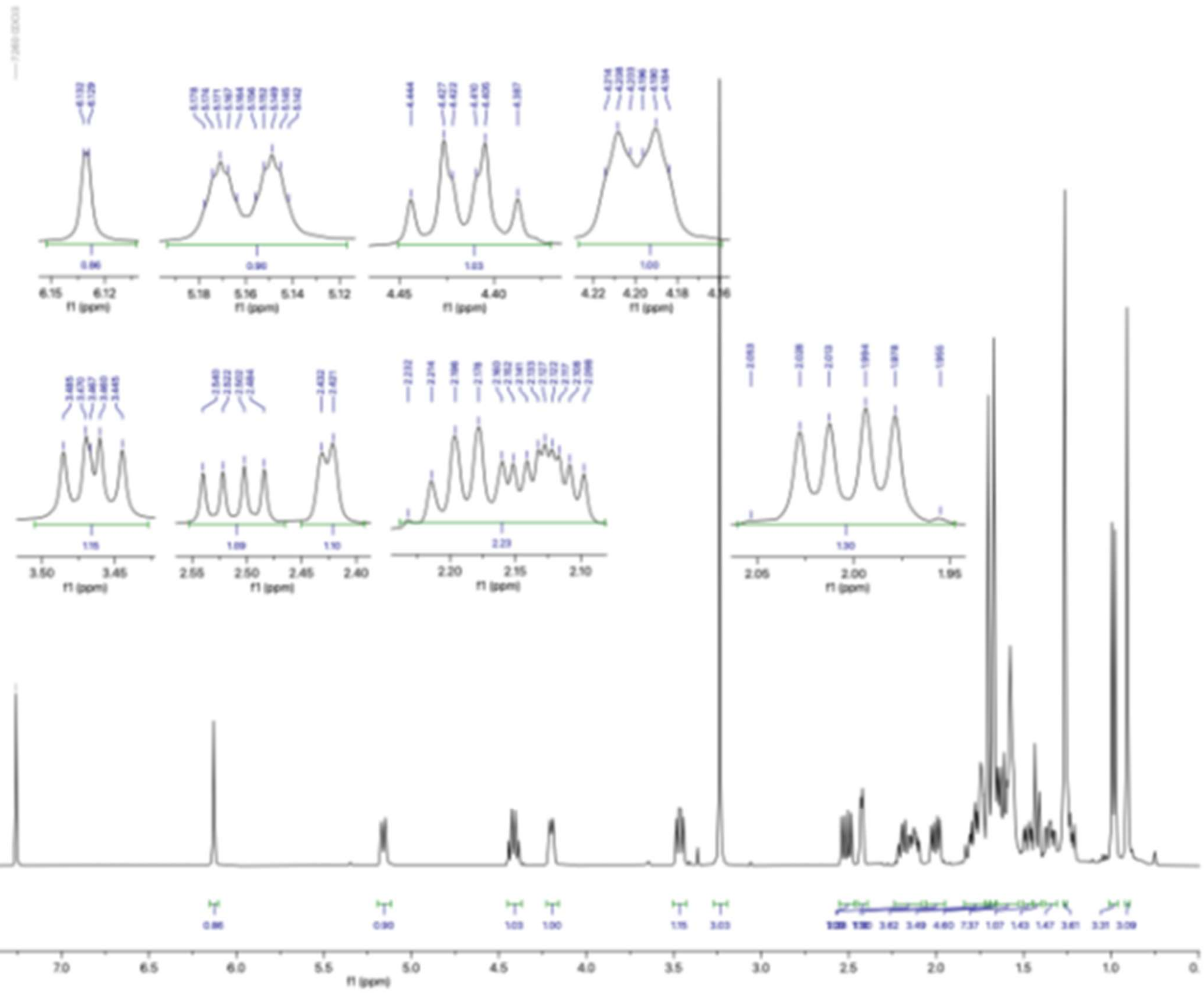

^{1}H NMR spectrum of maydispenoid A (**9**) (Bruker 600 MHZ, $CDCl_3$)

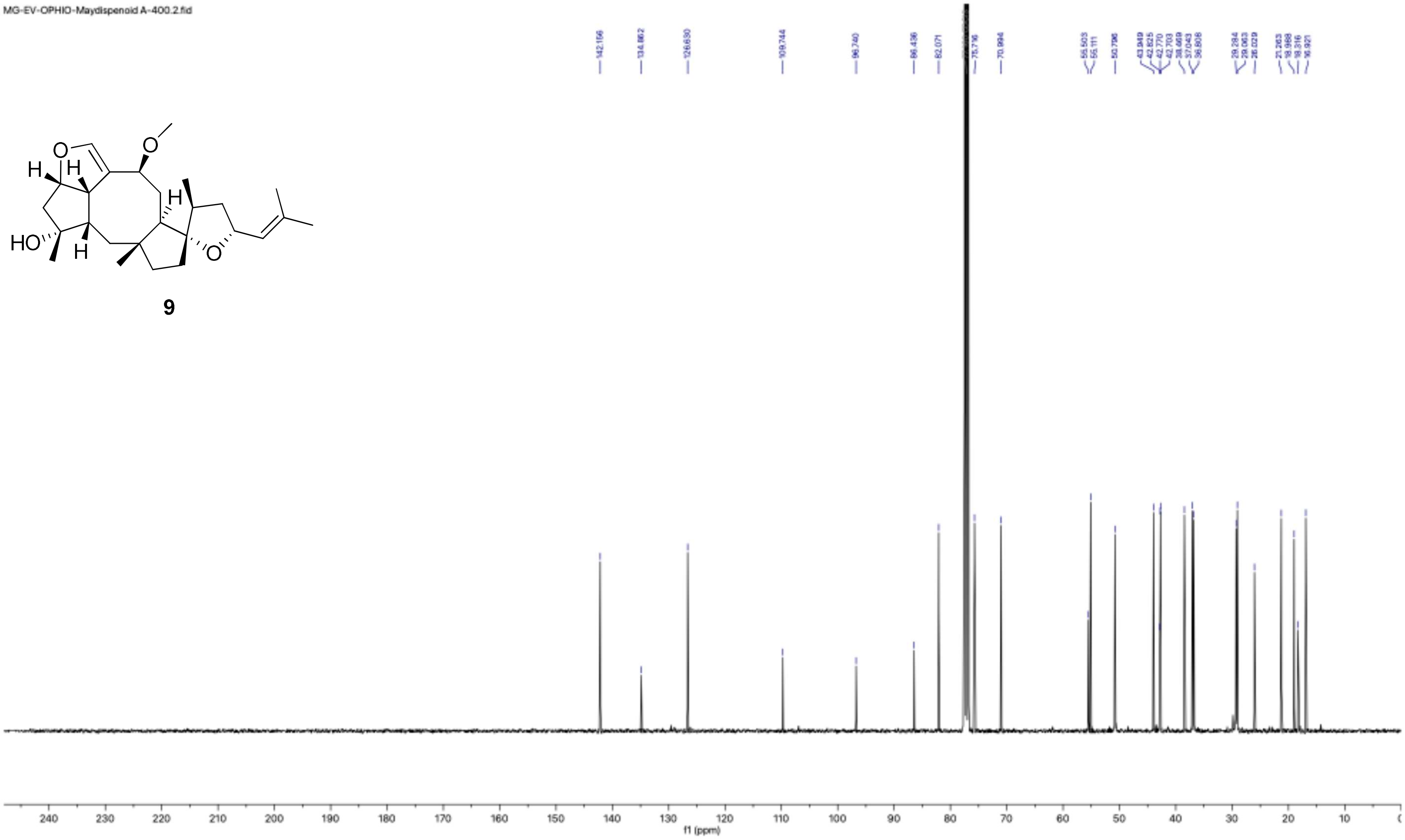

9

^{13}C NMR spectrum of maydispenoid A (**9**) (Bruker 400 MHZ, $CDCl_3$)

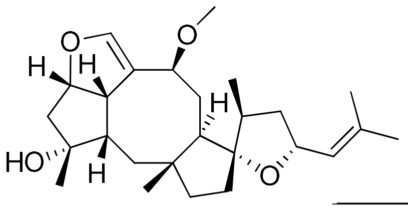

9

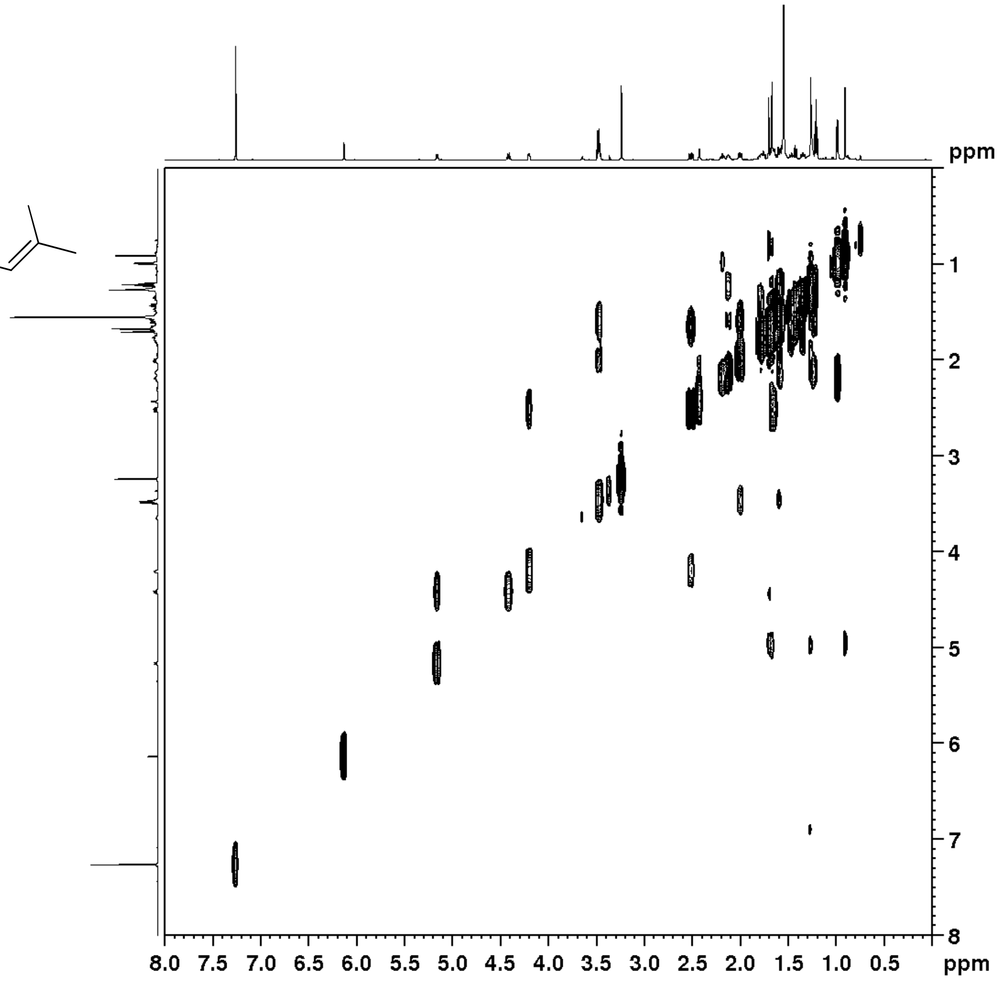

COSY spectrum of maydispenoid A (**9**) (Bruker 600 MHZ, $CDCl_3$)

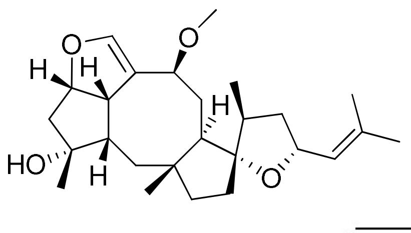

9

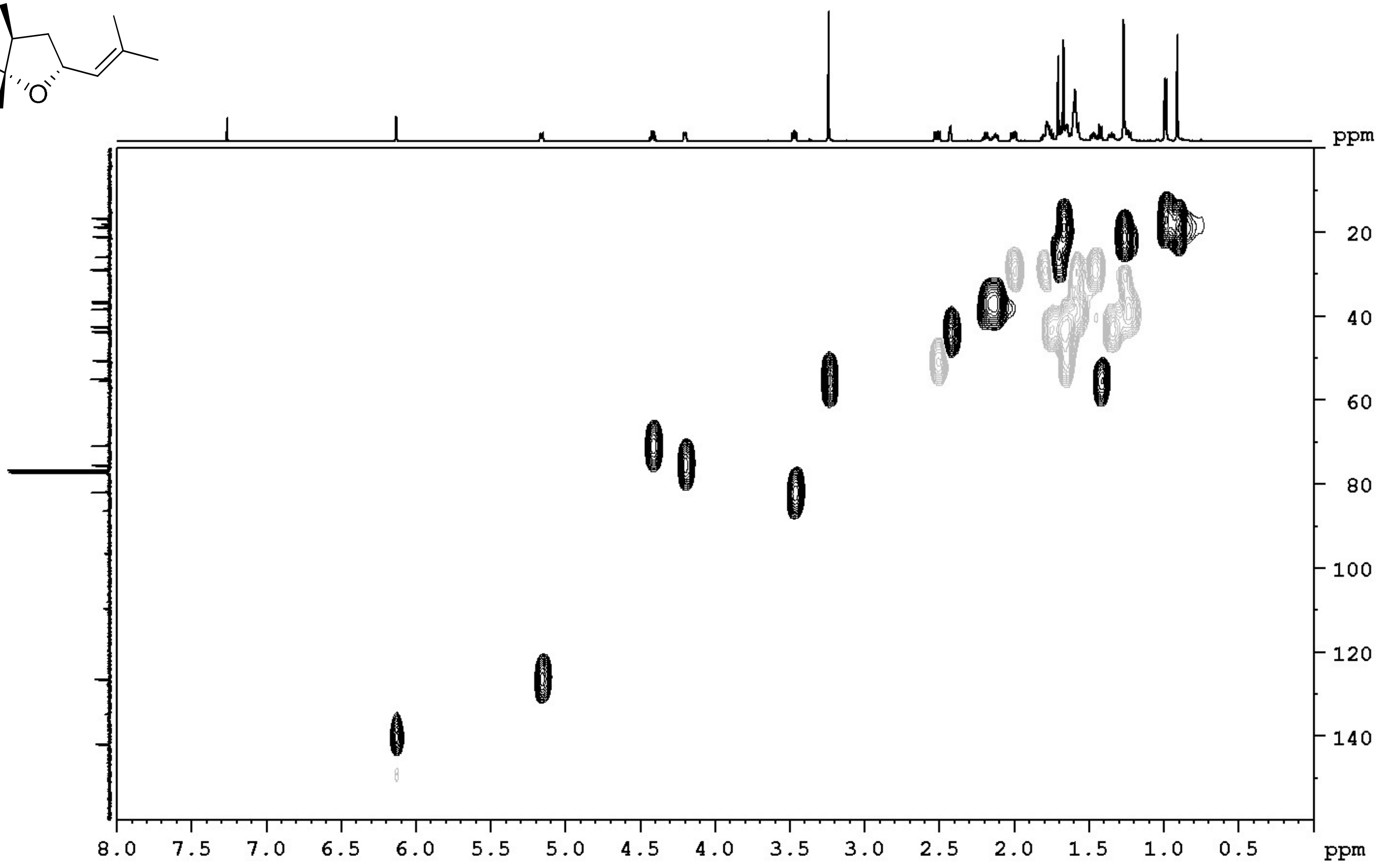

ed-HSQC spectrum of maydispenoid A (**9**) (Bruker 400 MHZ, $CDCl_3$)

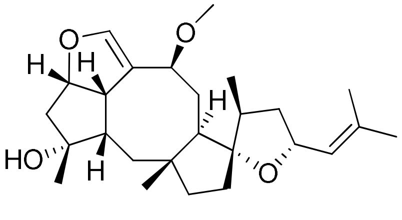

9

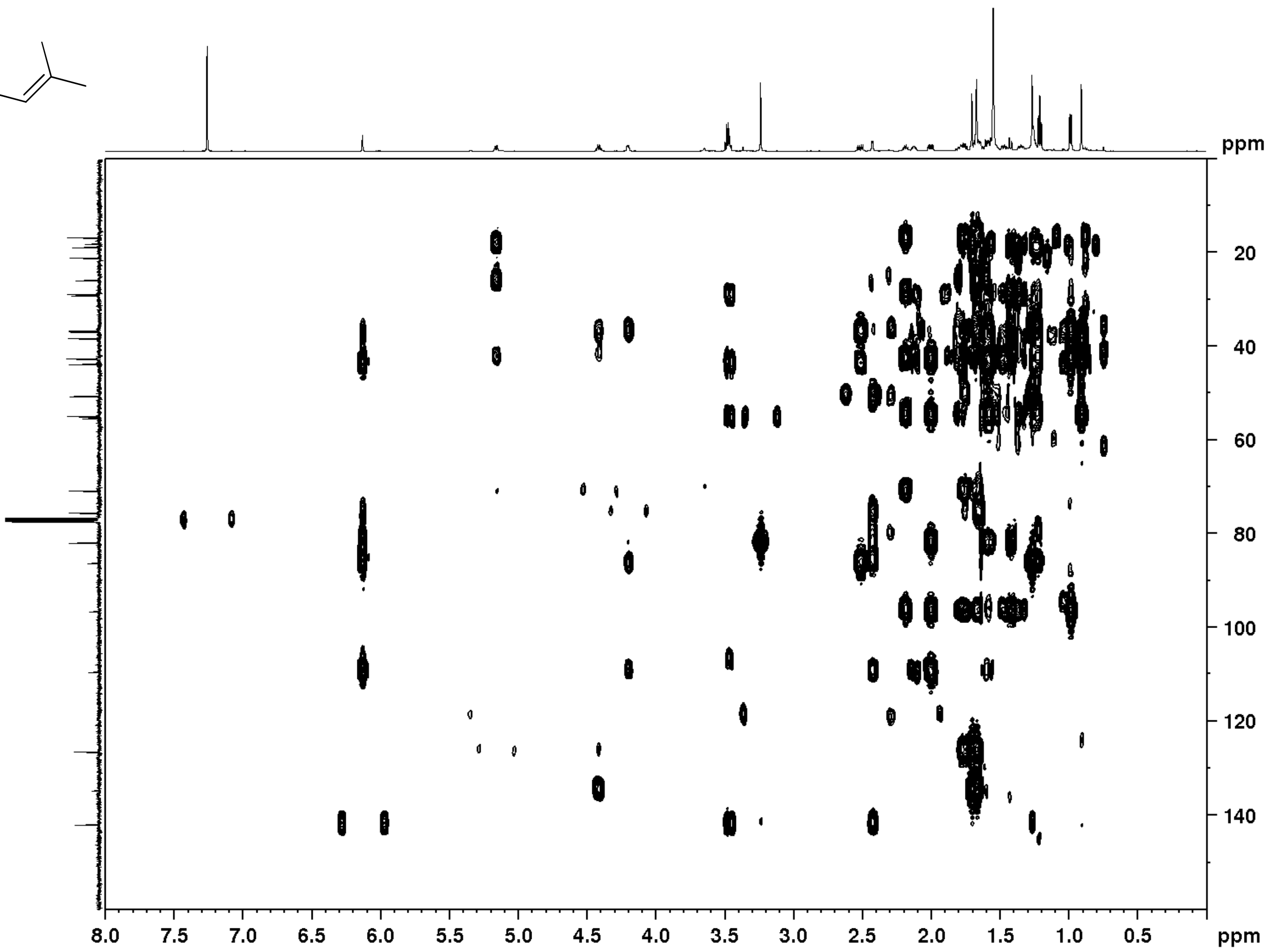

HMBC spectrum of maydispenoid A (**9**) (Bruker 600 MHZ, $CDCl_3$)

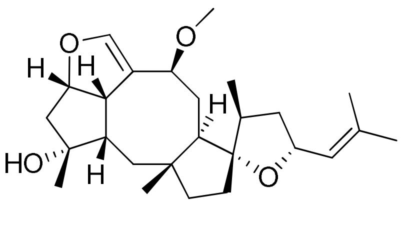

9

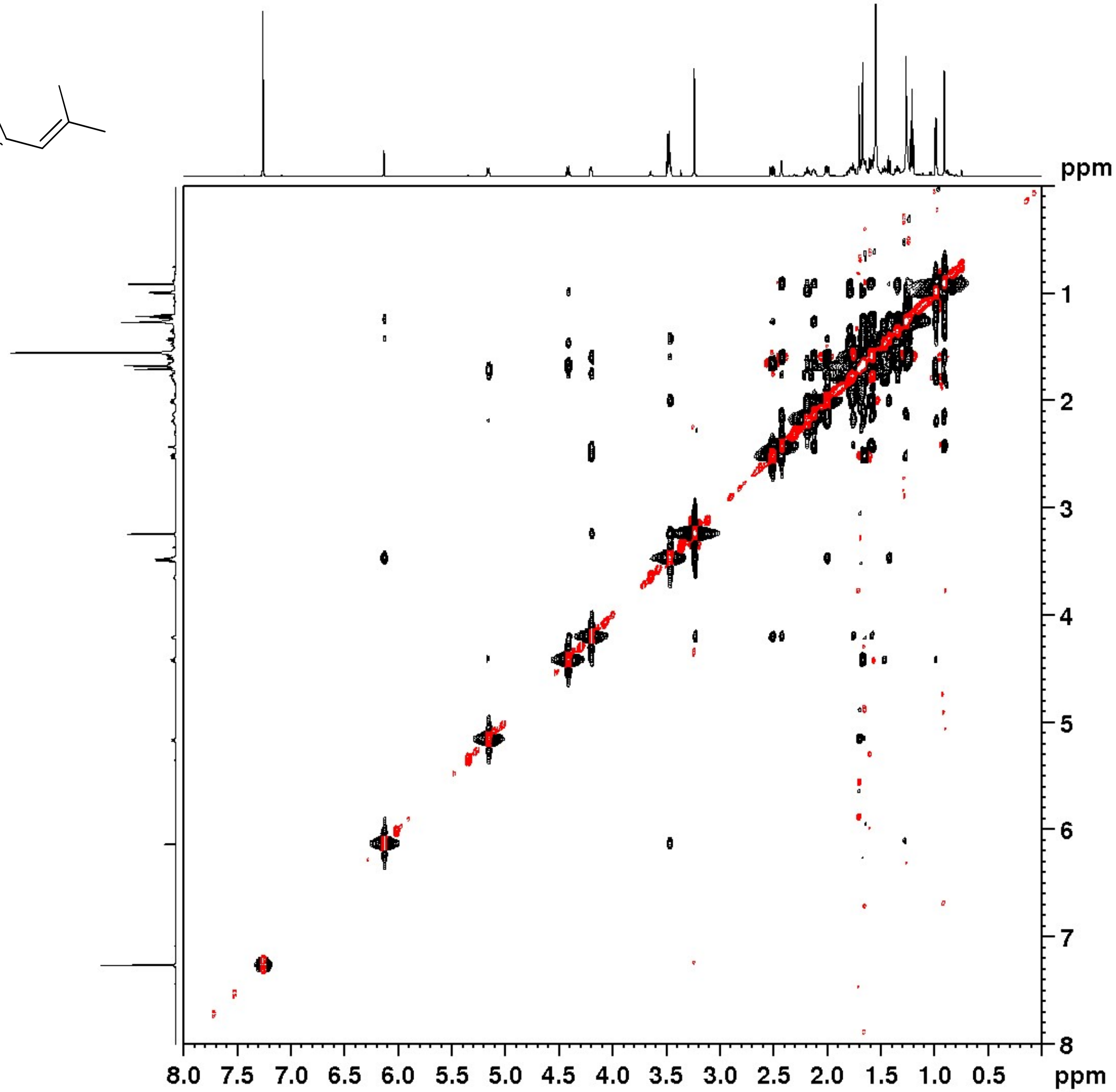

NOESY spectrum of maydispenoid A (**9**) (Bruker 600 MHZ, $CDCl_3$)

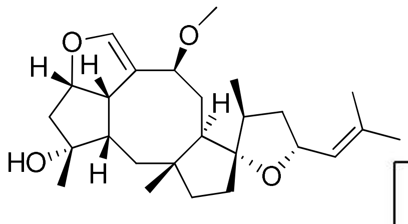

9

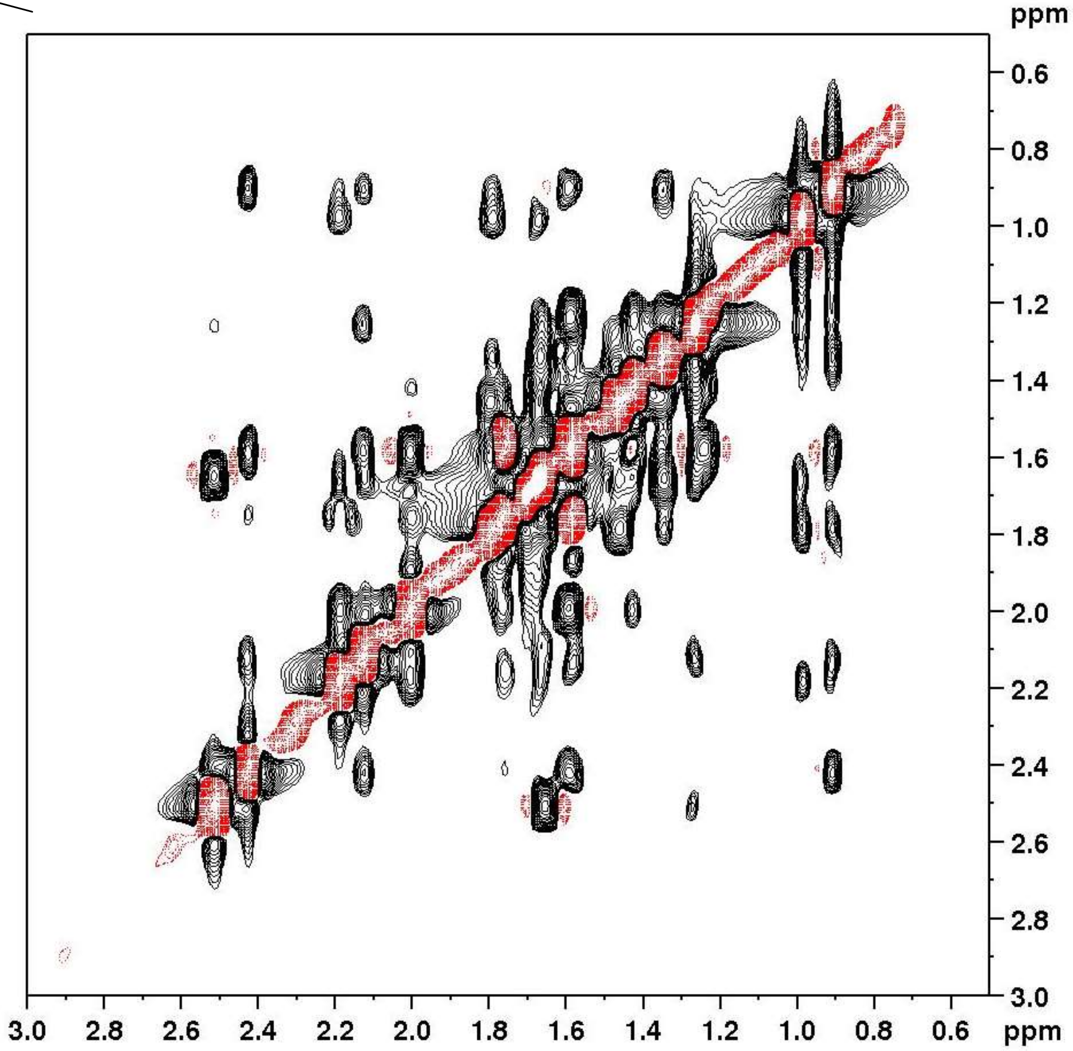

Upfield region expansion of the NOESY spectrum of maydispenoid A (**9**) (Bruker 600 MHZ, $CDCl_3$)

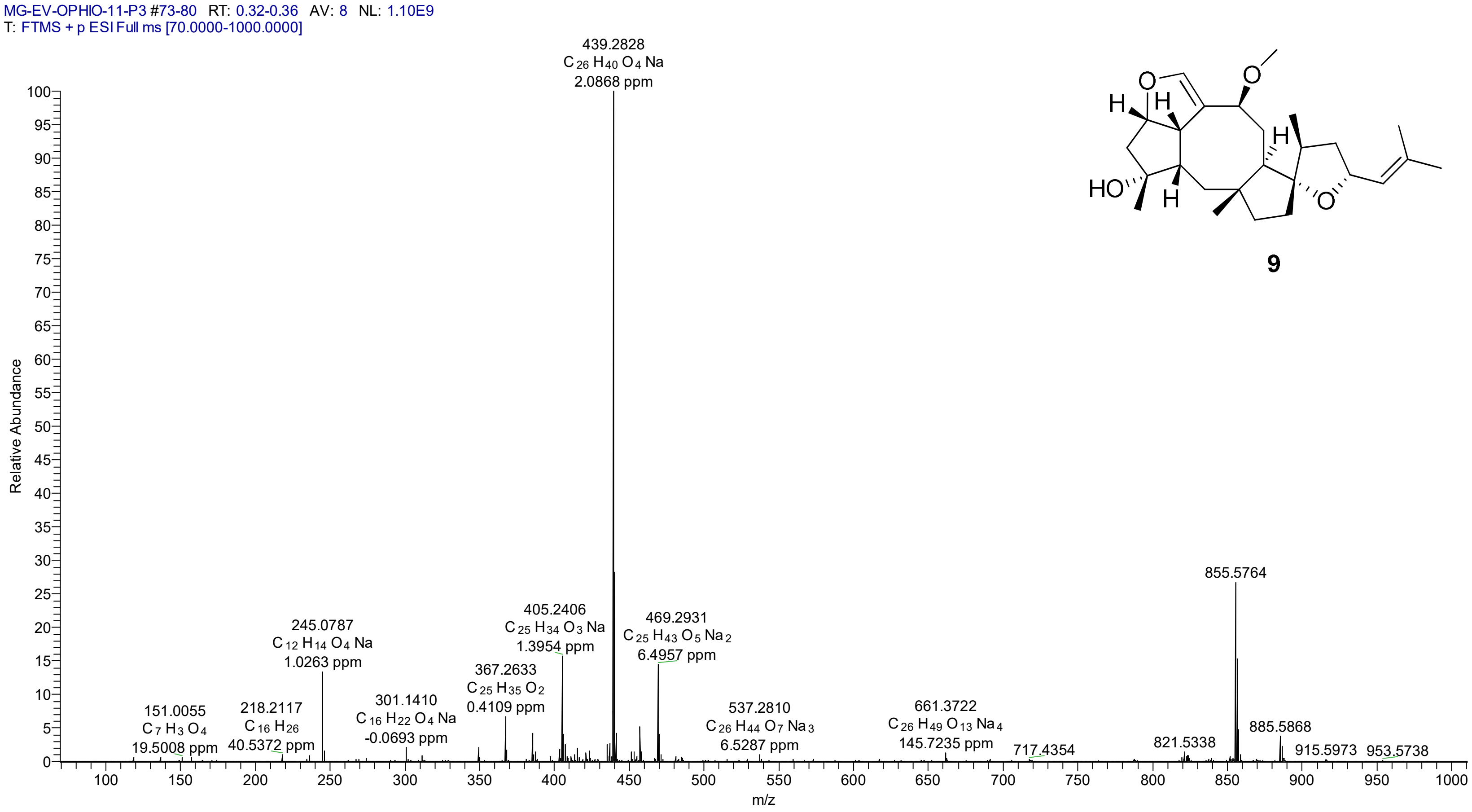

9

HRESI MS spectrum of maydispenoid A (**9**)

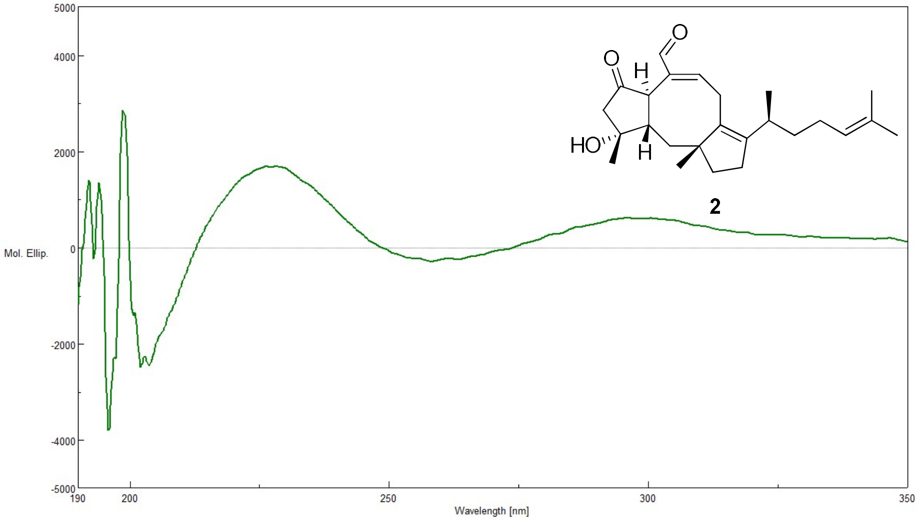

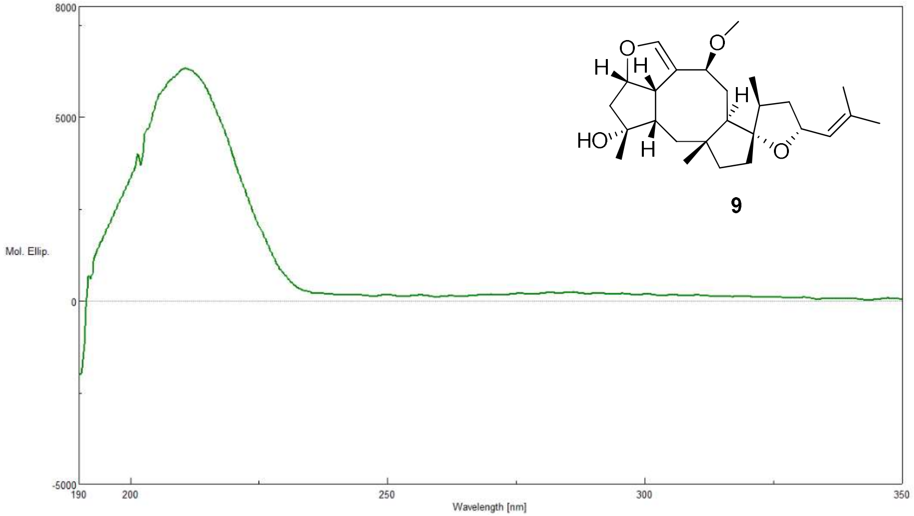

ECD spectra of gigobolin B (**2**) and maydispenoid A (**9**)
