# Supplementary material for: Gigobolins A–C, New Ophiobolins with Anticancer Activity from the Phytopathogenic Fungus Drechslera gigantea
Source: J Nat Prod. 2026 Feb 27;89(3):864–72. doi: 10.1021/acs.jnatprod.5c01414 (PMC13036769; doi:10.1021/acs.jnatprod.5c01414)
Supplement: Supplementary file 2 [file np5c01414_si_002.zip › Gigobolin A_MNR_RAW_DATA/NOESY_Gigo A/pdata/1/email_MG-EV-OPHIO-6-23-P1_7_1.pdf]

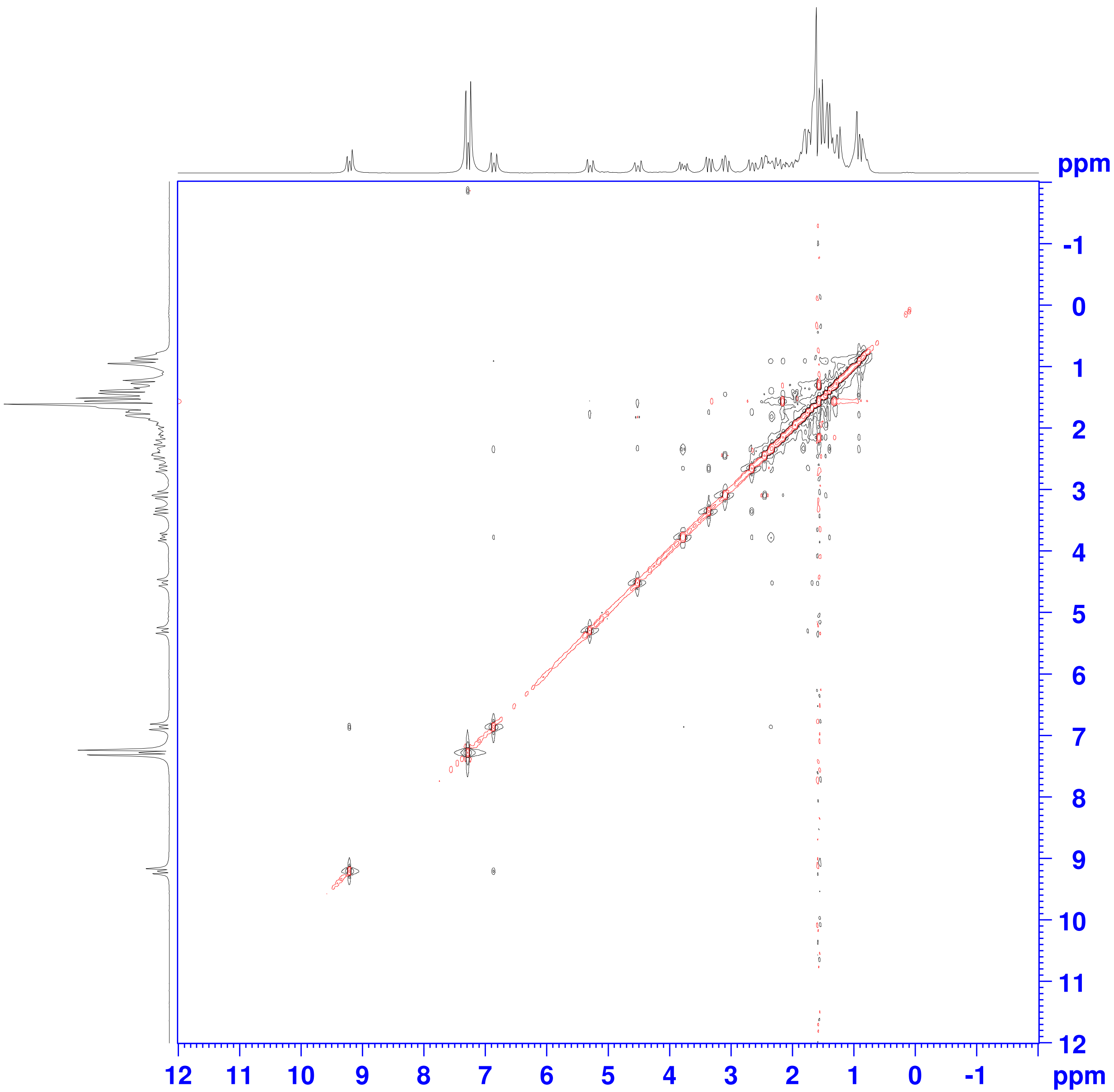

BRUKER

Current Data Parameters
NAME MG-EV-OPHIO-6-23-P1
EXPNO 7
PROCNO 1

F2 - Acquisition Parameters
Date_ 20250302
Time 18.54 h
INSTRUM spect
PROBHD Z44896_0016 (C
PULPROG noesygpph
TD 2048
SOLVENT CDCl3
NS 36
DS 4
SWH 8417.509 Hz
FIDRES 8.220223 Hz
AQ 0.1216512 sec
RG 32
DW 59.400 usec
DE 10.00 usec
TE 300.0 K
D0 0.00004991 sec
D1 1.00000000 sec
D8 0.30000001 sec
D16 0.00020000 sec
IN0 0.00011880 sec
TDav 1
SFO1 600.1330006 MHz
NUC1 1H
P1 7.45 usec
P2 14.90 usec
PLW1 8.19999981 W
GPNAM[1] SMSQ10.100
GPZ1 40.00 %
P16 1000.00 usec

F1 - Acquisition parameters
TD 256
SFO1 600.133 MHz
FIDRES 65.761787 Hz
SW 14.026 ppm
FnMODE States-TPPI

F2 - Processing parameters
SI 2048
SF 600.1300012 MHz
WDW GM
SSB 0
LB -18.00 Hz
GB 0.1
PC 1.00

F1 - Processing parameters
SI 2048
MC2 States-TPPI
SF 600.1300008 MHz
WDW GM
SSB 0
LB -18.00 Hz
GB 0.1
