# Supplementary figures and images for: Gigobolins A–C, New Ophiobolins with Anticancer Activity from the Phytopathogenic Fungus Drechslera gigantea
Source: J Nat Prod. 2026 Feb 27;89(3):864–72. doi: 10.1021/acs.jnatprod.5c01414 (PMC13036769; doi:10.1021/acs.jnatprod.5c01414)

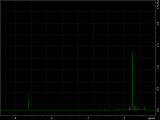

Supplement: Supplementary file 2 [file np5c01414_si_002.zip › Gigobolin A_MNR_RAW_DATA/1H_Gigo A/pdata/1/thumb.png]

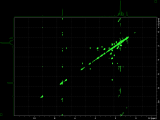

Supplement: Supplementary file 2 [file np5c01414_si_002.zip › Gigobolin A_MNR_RAW_DATA/COSY_Gigo A/pdata/1/thumb.png]

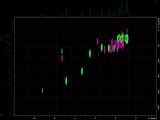

Supplement: Supplementary file 2 [file np5c01414_si_002.zip › Gigobolin A_MNR_RAW_DATA/ed_HSQC_Gigo A/pdata/1/thumb.png]

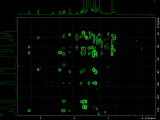

Supplement: Supplementary file 2 [file np5c01414_si_002.zip › Gigobolin A_MNR_RAW_DATA/HMBC_Gigo A/pdata/1/thumb.png]

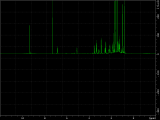

Supplement: Supplementary file 3 [file np5c01414_si_003.zip › Gigobolin B_NMR_RAW_DATA/1H_Gigo B/pdata/1/thumb.png]

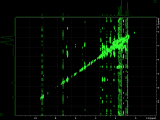

Supplement: Supplementary file 3 [file np5c01414_si_003.zip › Gigobolin B_NMR_RAW_DATA/COSY_Gigo B/pdata/1/thumb.png]

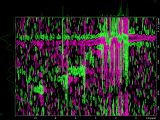

Supplement: Supplementary file 3 [file np5c01414_si_003.zip › Gigobolin B_NMR_RAW_DATA/ed_HSQC Gigo B/pdata/1/thumb.png]

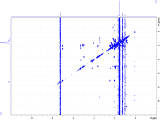

Supplement: Supplementary file 4 [file np5c01414_si_004.zip › Gigobolin C_NMR-RAW_DATA/COSY_Gigo C/pdata/1/thumb.png]

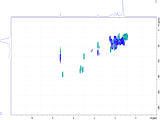

Supplement: Supplementary file 4 [file np5c01414_si_004.zip › Gigobolin C_NMR-RAW_DATA/ed_HSQC_Gigo C/pdata/1/thumb.png]

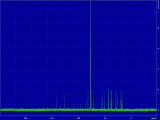

Supplement: Supplementary file 5 [file np5c01414_si_005.zip › Maydispenoid A_NMR_RAW_DATA/13C_Maydispenoid A/pdata/1/thumb.png]

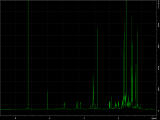

Supplement: Supplementary file 5 [file np5c01414_si_005.zip › Maydispenoid A_NMR_RAW_DATA/1H_Maydispenoid A/pdata/1/thumb.png]

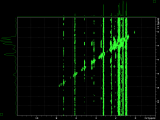

Supplement: Supplementary file 5 [file np5c01414_si_005.zip › Maydispenoid A_NMR_RAW_DATA/COSY_Maydispenoid A/pdata/1/thumb.png]

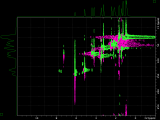

Supplement: Supplementary file 5 [file np5c01414_si_005.zip › Maydispenoid A_NMR_RAW_DATA/ed_HSQC_Maydispenoid A/pdata/1/thumb.png]

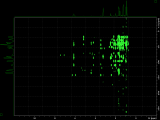

Supplement: Supplementary file 5 [file np5c01414_si_005.zip › Maydispenoid A_NMR_RAW_DATA/HMBC_Maydispenoid A/pdata/1/thumb.png]

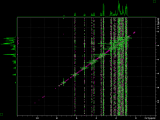

Supplement: Supplementary file 5 [file np5c01414_si_005.zip › Maydispenoid A_NMR_RAW_DATA/NOESY_Maydispenoid A/pdata/1/thumb.png]
