# Supplementary material for: Gigobolins A–C, New Ophiobolins with Anticancer Activity from the Phytopathogenic Fungus Drechslera gigantea
Source: J Nat Prod. 2026 Feb 27;89(3):864–72. doi: 10.1021/acs.jnatprod.5c01414 (PMC13036769; doi:10.1021/acs.jnatprod.5c01414)
Supplement: Supplementary file 3 [file np5c01414_si_003.zip › Gigobolin B_NMR_RAW_DATA/13C_Gigo B/pdata/1/email_MG-EV-OPHIO-13-P2_7_1.pdf]

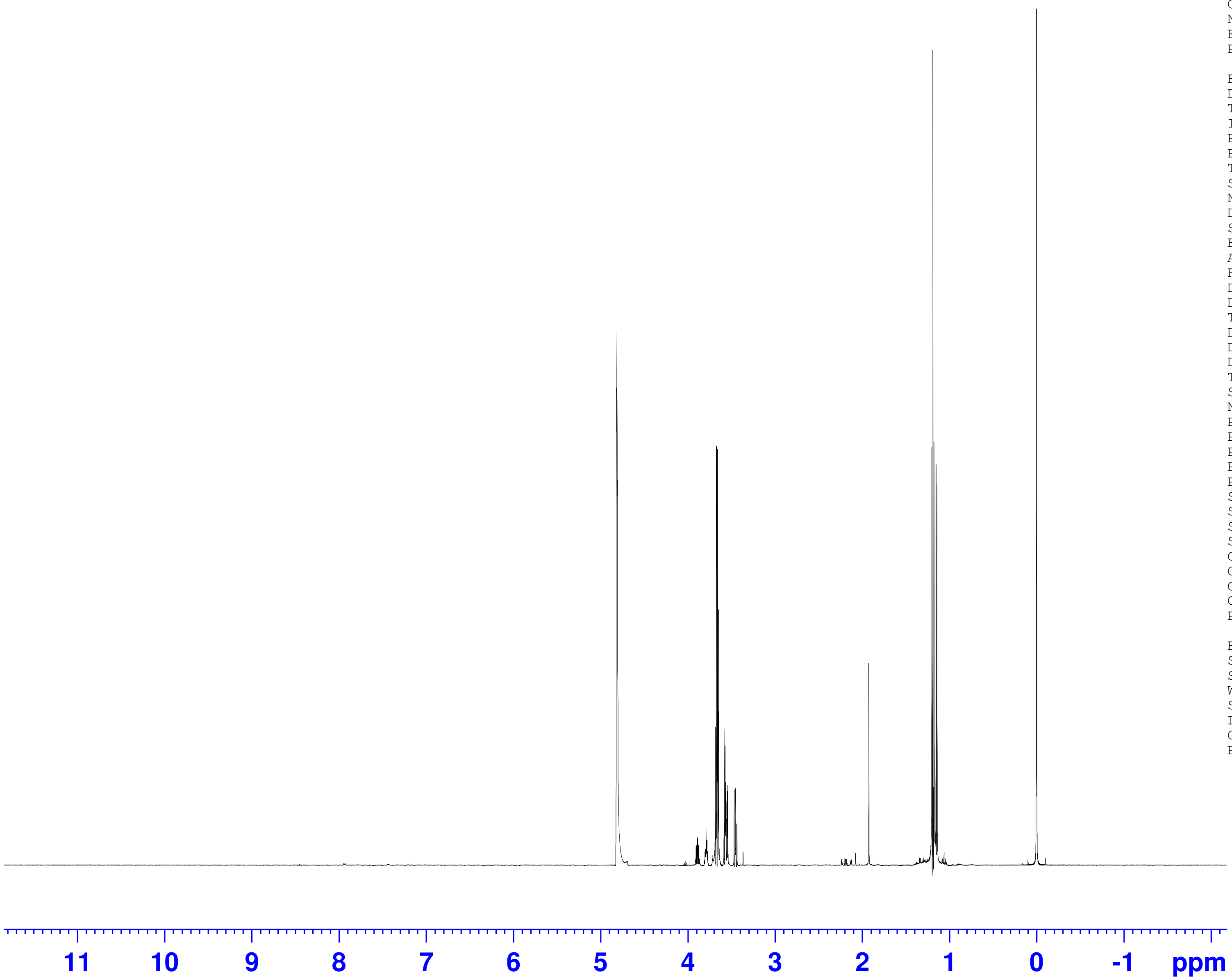

Current Data Parameters
NAME AM-A1Secr-C-H2O+D2O-19vii21
EXPNO 1
PROCNO 1

F2 - Acquisition Parameters
Date_ 20210719
Time 16.48 h
INSTRUM spect
PROBHD Z44896_0016 (C
PULPROG zgesgp
TD 16384
SOLVENT H2O+D2O
NS 512
DS 4
SWH 8417.509 Hz
FIDRES 1.027528 Hz
AQ 0.9732096 sec
RG 90.5
DW 59.400 usec
DE 10.00 usec
TE 298.0 K
D1 1.00000000 sec
D12 0.00002000 sec
D16 0.00020000 sec
TD0 1
SFO1 600.1328260 MHz
NUC1 1H
P1 8.76 usec
P2 17.52 usec
P12 2000.00 usec
PLW0 0 W
PLW1 7.44999981 W
SPNAM[1] Sinc1.1000
SPOAL1 0.500
SPOFFS1 0 Hz
SPW1 0.00165030 W
GPNAM[1] SMSQ10.100
GPZ1 31.00 %
GPNAM[2] SMSQ10.100
GPZ2 11.00 %
P16 1000.00 usec

F2 - Processing parameters
SI 32768
SF 600.1299271 MHz
WDW EM
SSB 0
LB 0.80 Hz
GB 0
PC 1.00
