# Supplementary material for: Gigobolins A–C, New Ophiobolins with Anticancer Activity from the Phytopathogenic Fungus Drechslera gigantea
Source: J Nat Prod. 2026 Feb 27;89(3):864–72. doi: 10.1021/acs.jnatprod.5c01414 (PMC13036769; doi:10.1021/acs.jnatprod.5c01414)
Supplement: Supplementary file 3 [file np5c01414_si_003.zip › Gigobolin B_NMR_RAW_DATA/HMBC_Gigo B/pdata/1/email_MG-EV-OPHIO-13-P2_4_1.pdf]

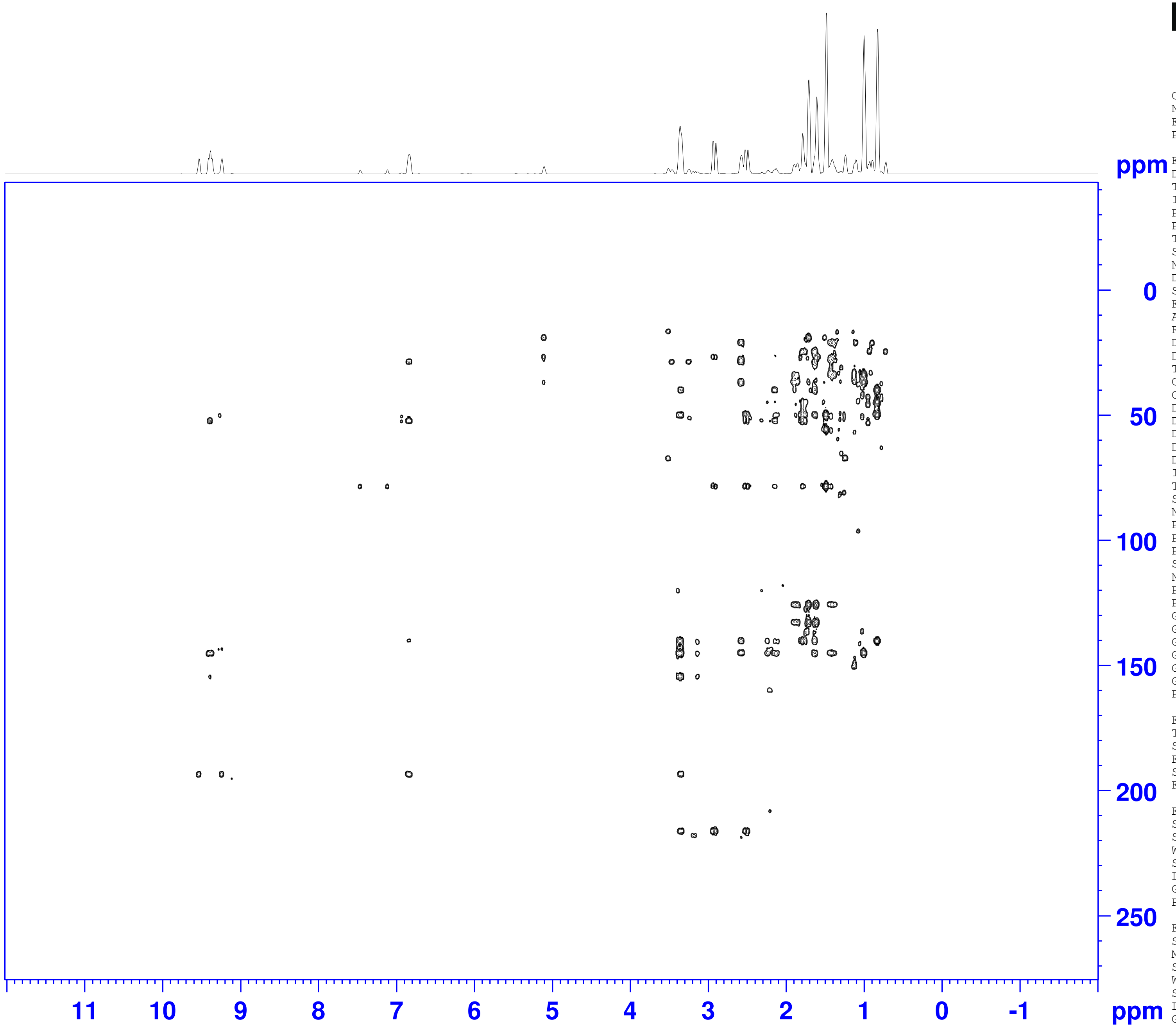

Current Data Parameters
NAME MG-EV-OPHIO-13-P2
EXPNO 4
PROCNO 1

F2 - Acquisition Parameters
Date_ 20250218
Time 17.09 h
INSTRUM spect
PROBHD Z44896_0016 (C
PULPROG hmbcgplpndqf
TD 2048
SOLVENT CDCl3
NS 64
DS 64
SWH 8417.509 Hz
FIDRES 8.220223 Hz
AQ 0.1216512 sec
RG 203
DW 59.400 usec
DE 10.00 usec
TE 300.0 K
CNST2 145.0000000
CNST13 7.0000000
D0 0.00000300 sec
D1 1.00000000 sec
D2 0.00344828 sec
D6 0.07142857 sec
D16 0.00020000 sec
IN0 0.00001040 sec
TDav 1
SFO1 600.1330006 MHz
NUC1 1H
P1 7.63 usec
P2 15.26 usec
PLW1 8.19999981 W
SFO2 150.9201628 MHz
NUC2 13C
P3 16.50 usec
PLW2 85.00000000 W
GPNAM[1] SMSQ10.100
GPZ1 50.00 %
GPNAM[2] SMSQ10.100
GPZ2 30.00 %
GPNAM[3] SMSQ10.100
GPZ3 40.10 %
P16 1000.00 usec

F1 - Acquisition parameters
TD 256
SFO1 150.9202 MHz
FIDRES 375.600952 Hz
SW 318.559 ppm
FnMODE QF

F2 - Processing parameters
SI 2048
SF 600.1299931 MHz
WDW QSINE
SSB 0
LB 0 Hz
GB 0
PC 1.40

F1 - Processing parameters
SI 2048
MC2 QF
SF 150.9026224 MHz
WDW QSINE
SSB 0
LB 0 Hz
GB 0
