# Supplementary material for: Gigobolins A–C, New Ophiobolins with Anticancer Activity from the Phytopathogenic Fungus Drechslera gigantea
Source: J Nat Prod. 2026 Feb 27;89(3):864–72. doi: 10.1021/acs.jnatprod.5c01414 (PMC13036769; doi:10.1021/acs.jnatprod.5c01414)
Supplement: Supplementary file 4 [file np5c01414_si_004.zip › Gigobolin C_NMR-RAW_DATA/13C_Gigo C/pdata/1/email_MG-EV-OPHIO-13-P8_3_1.pdf]

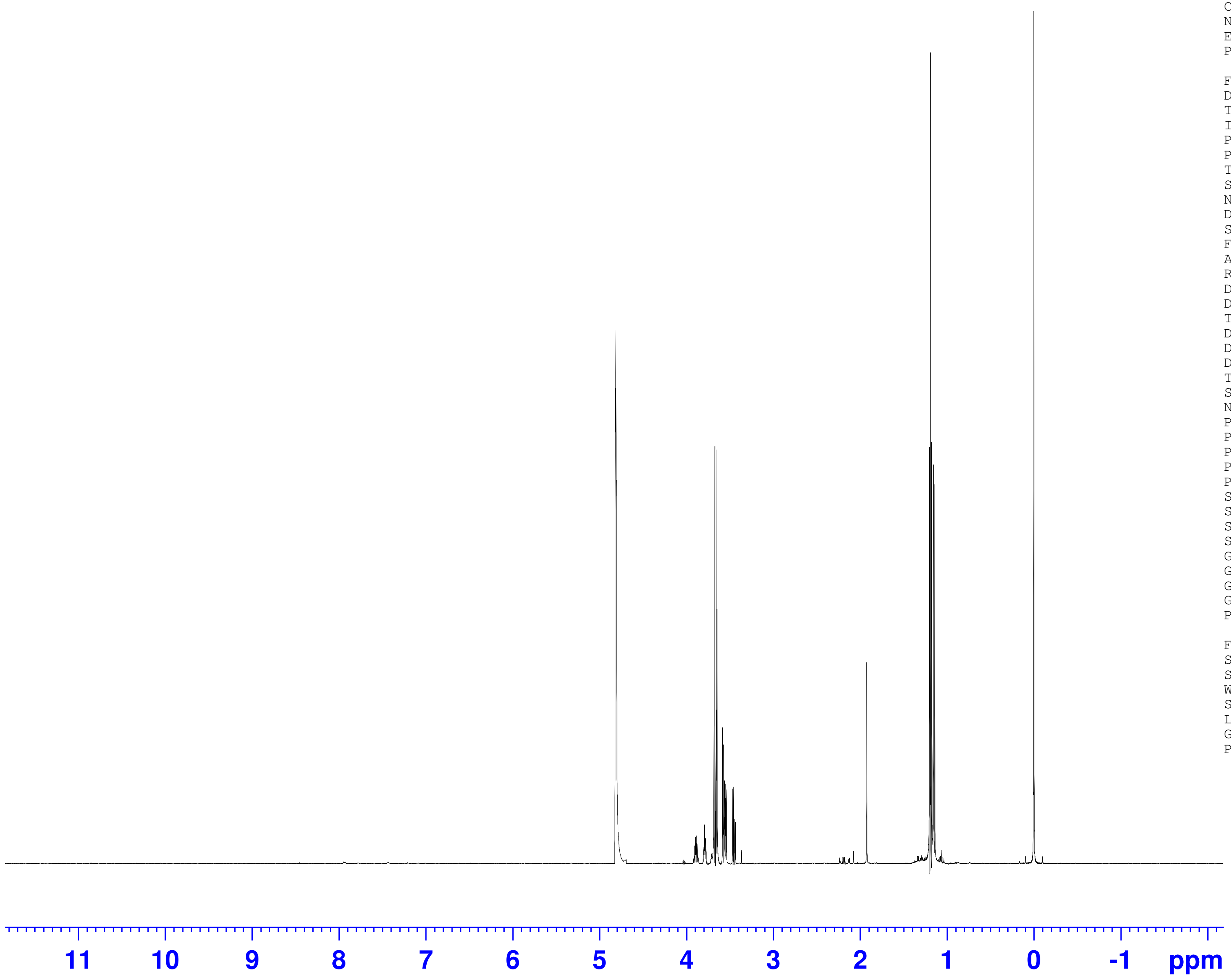

Current Data Parameters

NAME	AM-A1Secr-C-H2O+D2O-19vii21
EXPNO	1
PROCNO	1

F2 - Acquisition Parameters

Date_	20210719
Time	16.48 h
INSTRUM	spect
PROBHD	Z44896_0016 (C
PULPROG	zgesgp
TD	16384
SOLVENT	H2O+D2O
NS	512
DS	4
SWH	8417.509 Hz
FIDRES	1.027528 Hz
AQ	0.9732096 sec
RG	90.5
DW	59.400 usec
DE	10.00 usec
TE	298.0 K
D1	1.00000000 sec
D12	0.00002000 sec
D16	0.00020000 sec
TD0	1
SFO1	600.1328260 MHz
NUC1	1H
P1	8.76 usec
P2	17.52 usec
P12	2000.00 usec
PLW0	0 W
PLW1	7.44999981 W
SPNAM[1]	Sinc1.1000
SPOAL1	0.500
SPOFFS1	0 Hz
SPW1	0.00165030 W
GPNAM[1]	SMSQ10.100
GPZ1	31.00 %
GPNAM[2]	SMSQ10.100
GPZ2	11.00 %
P16	1000.00 usec

F2 - Processing parameters

SI	32768
SF	600.1299271 MHz
WDW	EM
SSB	0
LB	0.80 Hz
GB	0
PC	1.00
