# Supplementary material for: Gigobolins A–C, New Ophiobolins with Anticancer Activity from the Phytopathogenic Fungus Drechslera gigantea
Source: J Nat Prod. 2026 Feb 27;89(3):864–72. doi: 10.1021/acs.jnatprod.5c01414 (PMC13036769; doi:10.1021/acs.jnatprod.5c01414)
Supplement: Supplementary file 4 [file np5c01414_si_004.zip › Gigobolin C_NMR-RAW_DATA/HMBC_Gigo C/pdata/1/email_MG-EV-OPHIO-13-P8_2_1.pdf]

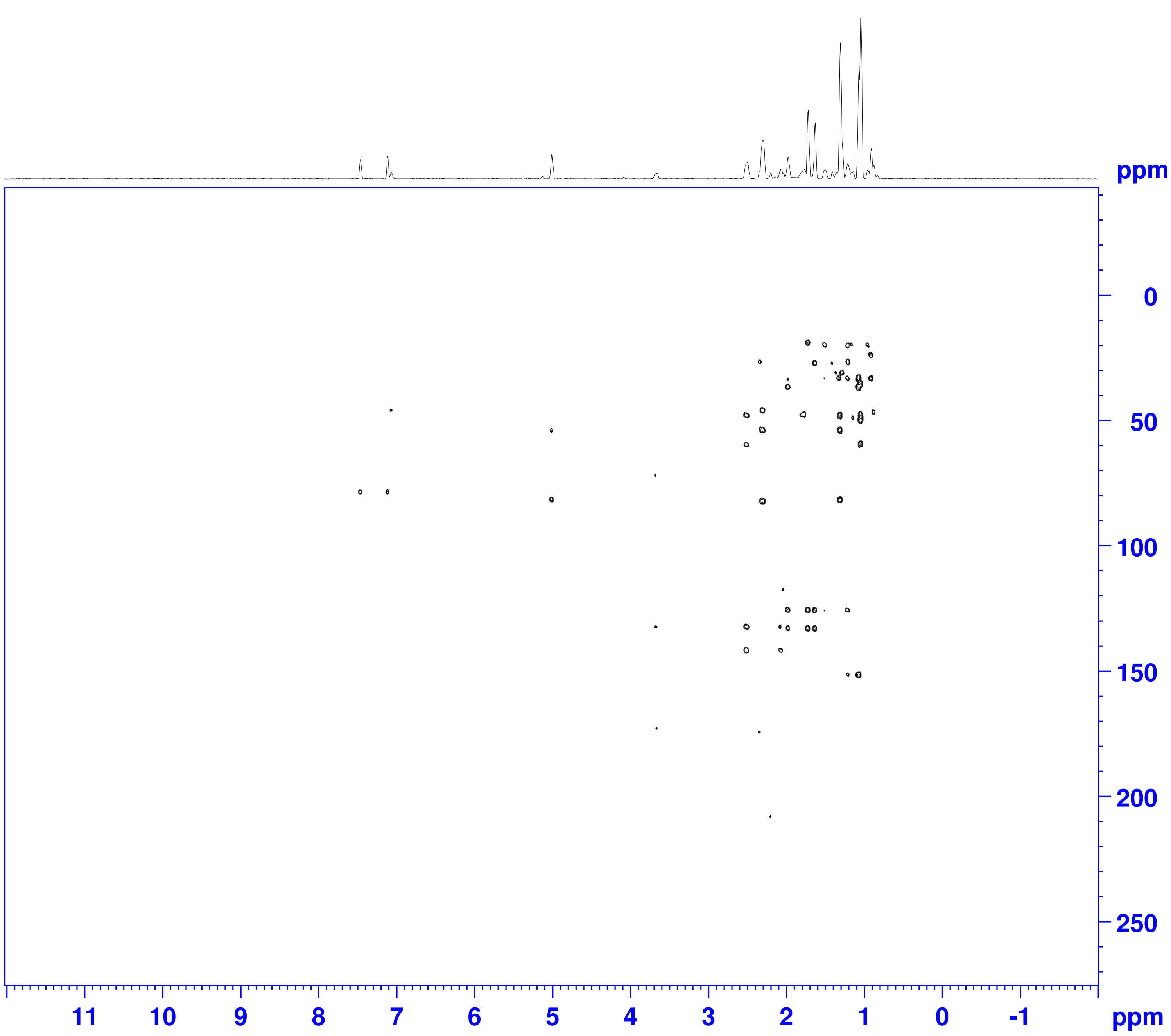

Current Data Parameters
NAME MG-EV-OPHIO-13-P8
EXPNO 2
PROCNO 1

F2 - Acquisition Parameters

Date_	20250220	
Time	0.06	h
INSTRUM	spect	
PROBHD	Z44896_0016 (C	
PULPROG	hmbcgplpndqf	
TD	2048	
SOLVENT	CDCl3	
NS	100	
DS	64	
SWH	8417.509	Hz
FIDRES	8.220223	Hz
AQ	0.1216512	sec
RG	203	
DW	59.400	usec
DE	10.00	usec
TE	300.0	K
CNST2	145.0000000	
CNST13	7.0000000	
D0	0.00000300	sec
D1	1.00000000	sec
D2	0.00344828	sec
D6	0.07142857	sec
D16	0.00020000	sec
IN0	0.00001040	sec
TDav	1	
SFO1	600.1330006	MHz
NUC1	1H	
P1	7.83	usec
P2	15.66	usec
PLW1	8.19999981	W
SFO2	150.9201628	MHz
NUC2	13C	
P3	16.50	usec
PLW2	85.00000000	W
GPNAM[1]	SMSQ10.100	
GPZ1	50.00	%
GPNAM[2]	SMSQ10.100	
GPZ2	30.00	%
GPNAM[3]	SMSQ10.100	
GPZ3	40.10	%
P16	1000.00	usec

F1 - Acquisition parameters

TD	256	
SFO1	150.9202	MHz
FIDRES	375.600952	Hz
SW	318.559	ppm
FnMODE	QF	

F2 - Processing parameters

SI	2048	
SF	600.1299931	MHz
WDW	QSINE	
SSB	0	
LB	0	Hz
GB	0	
PC	1.40	

F1 - Processing parameters

SI	2048	
MC2	QF	
SF	150.9026224	MHz
WDW	QSINE	
SSB	0	
LB	0	Hz
GB	0	
