# Supplementary material for: Gigobolins A–C, New Ophiobolins with Anticancer Activity from the Phytopathogenic Fungus Drechslera gigantea
Source: J Nat Prod. 2026 Feb 27;89(3):864–72. doi: 10.1021/acs.jnatprod.5c01414 (PMC13036769; doi:10.1021/acs.jnatprod.5c01414)
Supplement: Supplementary file 5 [file np5c01414_si_005.zip › Maydispenoid A_NMR_RAW_DATA/NOESY_Maydispenoid A/pdata/1/email_MG-EV-OPHIO-11-P3_7_1.pdf]

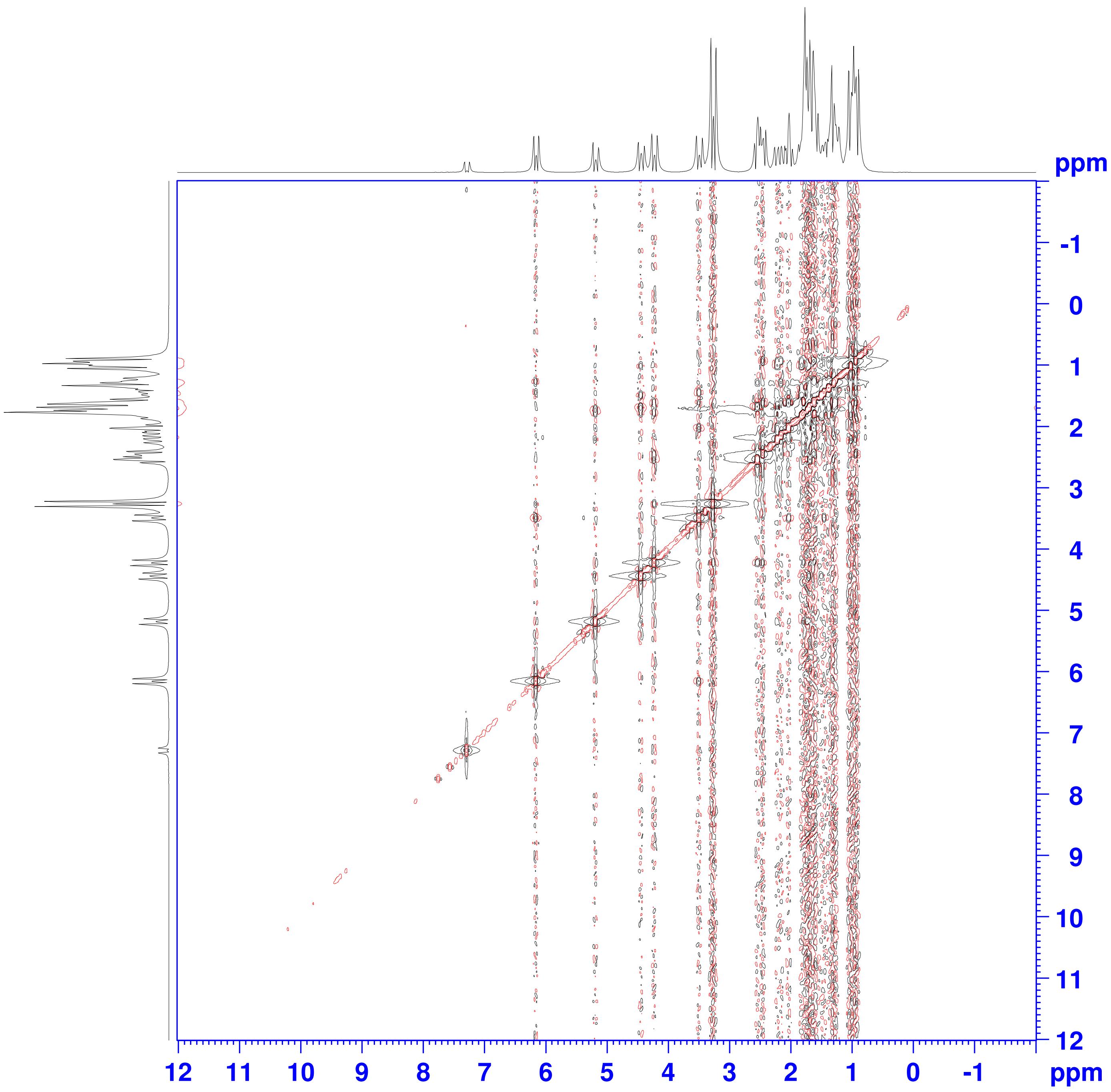

Current Data Parameters

NAME	MG-EV-OPHIO-11-P3
EXPNO	7
PROCNO	1

F2 - Acquisition Parameters

Date_	20250226
Time	5.51 h
INSTRUM	spect
PROBHD	Z44896_0016 (C
PULPROG	noesygpph
TD	2048
SOLVENT	CDCl3
NS	28
DS	4
SWH	8417.509 Hz
FIDRES	8.220223 Hz
AQ	0.1216512 sec
RG	32
DW	59.400 usec
DE	10.00 usec
TE	300.0 K
D0	0.00004960 sec
D1	1.00000000 sec
D8	0.50000000 sec
D16	0.00020000 sec
IN0	0.00011880 sec
TDav	1
SFO1	600.1330006 MHz
NUC1	1H
P1	7.70 usec
P2	15.40 usec
PLW1	8.19999981 W
GPNAM[1]	SMSQ10.100
GPZ1	40.00 %
P16	1000.00 usec

F1 - Acquisition parameters

TD	256
SFO1	600.133 MHz
FIDRES	65.761787 Hz
SW	14.026 ppm
FnMODE	States-TPPI

F2 - Processing parameters

SI	2048
SF	600.1299958 MHz
WDW	GM
SSB	0
LB	-18.00 Hz
GB	0.1
PC	1.00

F1 - Processing parameters

SI	2048
MC2	States-TPPI
SF	600.1299967 MHz
WDW	GM
SSB	0
LB	-18.00 Hz
GB	0.1
